# Transcriptional outcomes and kinetic patterning of gene expression in response to NF-κB activation

**DOI:** 10.1371/journal.pbio.2006347

**Published:** 2018-09-10

**Authors:** Mingming Zhao, Jaimy Joy, Weiqiang Zhou, Supriyo De, William H. Wood, Kevin G. Becker, Hongkai Ji, Ranjan Sen

**Affiliations:** 1 Gene Regulation Section, Laboratory of Molecular Biology and Immunology, National Institute on Aging, Baltimore, Maryland, United States of America; 2 Department of Biostatistics, Johns Hopkins University Bloomberg School of Public Health, Baltimore, Maryland, United States of America; 3 Gene Expression and Genomics Unit, Laboratory of Genetics and Genomics, National Institute on Aging, Baltimore, Maryland, United States of America; University of California San Diego, United States of America

## Abstract

Transcription factor nuclear factor kappa B (NF-κB) regulates cellular responses to environmental cues. Many stimuli induce NF-κB transiently, making time-dependent transcriptional outputs a fundamental feature of NF-κB activation. Here we show that NF-κB target genes have distinct kinetic patterns in activated B lymphoma cells. By combining RELA binding, RNA polymerase II (Pol II) recruitment, and perturbation of NF-κB activation, we demonstrate that kinetic differences amongst early- and late-activated RELA target genes can be understood based on chromatin configuration prior to cell activation and RELA-dependent priming, respectively. We also identified genes that were repressed by RELA activation and others that responded to RELA-activated transcription factors. Cumulatively, our studies define an NF-κB-responsive inducible gene cascade in activated B cells.

## Author summary

The nuclear factor kappa B (NF-κB) family of transcription factors regulates cellular responses to a wide variety of environmental cues. These could be extracellular stimuli that activate cell surface receptors, such as pathogens, or intracellular stress signals such as DNA damage or oxidative stress. In response to these triggers, NF-κB proteins accumulate in the cell nucleus, bind to specific DNA sequences in the genome, and thereby modulate gene transcription. Because of the diversity of signals that activate NF-κB and the ubiquity of this pathway in most cell types, cellular outcomes via NF-κB activation must be finely tuned to respond to the initiating stimulus. One mechanism by which NF-κB-dependent gene expression is regulated is by varying the duration of nuclear NF-κB; some signals lead to persistent nuclear NF-κB, while others lead to transient nuclear NF-κB. Consequently, time dependency of transcriptional responses is a unique signature of the initiating stimulus. Here we probed mechanisms that generate kinetic patterns of NF-κB-dependent gene expression in B lymphoma cells responding to a transient NF-κB-activating stimulus. By genetically manipulating NF-κB induction, we identified direct targets of RELA, a member of the NF-κB family, and provide evidence that kinetic patterns are established by a combination of factors that include the chromatin state of genes prior to cell activation and cofactors that work with RELA.

## Introduction

The family of nuclear factor kappa B (NF-κB) transcription factors regulates inducible gene transcription in response to diverse stimuli. Signals from receptors ultimately activate inhibitor of NF-κB kinases (IKKs) that phosphorylate a variety NFKB inhibitors (IκBs), targeting them for degradation and leading to accumulation of NF-κB family members in the nucleus. The “classical” pathway, via IKK2 activation, results in nuclear translocation of RelA- or Rel-containing NF-κB proteins, whereas the nonclassical pathway, via IKK1 activation, results in nuclear accumulation of RelB-containing NF-κB proteins [1,2]. Some signals only activate IKK1 (such as B cell activating factor [BAFF]/BAFF receptor [BAFF-R]), others only activate IKK2 (such as tumor necrosis factor alpha [TNFα] and interleukin 1 beta [IL-1β]), and yet others activate both IKK1 and IKK2 (such as CD40/CD40L interaction). The cellular response to NF-κB activation therefore depends on the nature of the stimulus and the associated pattern of NF-κB proteins that are driven to the nucleus. Despite identification of a handful of well-accepted NF-κB target genes (such as *NFKBIA*, *TNFAIP3*, and *MYC*), genome-wide transcriptional responses mediated by NF-κB remain poorly defined.

There are several reasons for this. First, the time course of NF-κB induction varies greatly depending on stimulus. For example, classical NF-κB activation by TNFα or IL-1β is rapid and transient, whereas activation via Toll-like receptor 4 (TLR4) is slower and more sustained [1,3]. Second, NF-κB proteins consist of several family members. Nuclear factor kappa B subunit 1 (NFKB1), RELA, and REL proteins respond primarily by the classical pathway, whereas nuclear factor kappa B subunit 2 (NFKB2) and RELB respond to nonclassical activation. Thus, a comprehensive analysis must include gene targets of each family member. This inherent complexity is compounded by observations that some genes that encode Rel family members, such as *NFKB1*, *NFKB2*, and *RELB*, are themselves targets of NF-κB. Third, NF-κB responses vary depending on the cell type as well as the initiating stimulus. Cell type specificity of NF-κB targets in monocytes/macrophages has been proposed to be conferred by regulated access of induced NF-κB to a subset of genomic sites by tissue-specific transcription factors [4–6]. Stimulus specificity has been explored largely in the context of TLR signaling and attributed to differences in dynamic patterns of NF-κB induction [7–9]. Yet, connections between NF-κB dynamics and transcriptional output are not well understood.

For genes to be classified as NF-κB targets, they must change in expression as a consequence of NF-κB binding in cells that have received 1 or more NF-κB-inducing stimuli. This requires integrating at least 3 variables: transcriptional outcomes, NF-κB binding, and the consequences of abolishing NF-κB binding. The first 2 criteria have been explored by microarray or RNA sequencing (RNA-Seq) and by chromatin immunoprecipitation and sequencing (ChIP-Seq) to assay transcription factor binding genome-wide. For NF-κB, the majority of ChIP-Seq studies used TNFα as the NF-κB-inducing stimulus in HeLa cells or endothelial cells, with stimulus times ranging from 1 to 6 h. In response to TNFα, RELA bound to approximately 1,200–12,500 sites genome-wide in different studies, with the majority of binding occurring at sites other than gene promoters [10–19]. One of the earliest RELA ChIP-Seq studies also noted that the factor bound at many genes whose expression was unaffected by TNFα treatment [20]. Sites of RELA binding were enriched not only for the κB motif (GGGRNYYYCC) but also for recognition sites of other transcription factors, especially activator protein 1 (AP1). The latter observation corroborated previously reported interactions between NF-κB and AP1 [21,22]. Additionally, E2F and forkhead box M1 (FoxM1) transcription factor motifs have been identified within RELA binding regions [20,23]. The RelA response has also been characterized in murine macrophages treated with lipopolysaccharide (LPS) for 1–3 h [4,5,24,25]. A substantial proportion of sites to which RelA was recruited in these cells were found to have prebound PU.1, a macrophage-enriched transcription factor. In addition, recognition motifs of interferon regulatory factors (IRFs) and AP1 were enriched at RelA-bound sites in macrophages. Such differences have been proposed to underlie selectivity of NF-κB responses.

The third criterion, that of establishing that transcriptional outcome is a consequence of NF-κB binding, remains largely underexplored. The most precise way to causally connect binding events to gene expression requires mutating these sites in genomic DNA followed by transcriptional analyses. This is virtually impossible to do on a genome-wide scale. A more feasible, yet meaningful, alternative is to monitor transcriptional consequences of depleting the transcription factor, such as the use of RelA-deficient macrophages to validate the role of RelA in the LPS response [24]. Additionally, most studies do not account for the impact of dynamic patterns of RELA induction on inducible gene transcription. For this, both NF-κB binding and RNA levels must be interrogated at multiple time points in response to a stimulus. This is especially true for stimuli that activate NF-κB transiently.

Here we probed transcriptional responses to a transient NF-κB-inducing stimulus by combining data from kinetic analyses of RELA binding, RNA polymerase recruitment, transcriptional output, and perturbation of classical NF-κB activation. Using BJAB B lymphoma cells, we demonstrate that NF-κB/RELA is transiently recruited to nearly 3,000 sites genome-wide in response to pharmacological mimics of B cell antigen receptor activation. From these sites, we identified several hundred genes that were direct transcriptional targets of NF-κB. Most of these genes were not found in databases of putative NF-κB target genes. Though the majority of functional NF-κB target genes were up-regulated by RELA, we also identified genes whose expression was suppressed by RELA binding. RELA target genes displayed different transcriptional kinetics, and most recruited RNA polymerase II (Pol II) in response to cell activation. In querying the basis for kinetic differences, we found that late-activated NF-κB target genes required extracellular signal–regulated kinase (ERK) activity, whereas rapidly induced NF-κB target genes were marked by Pol II–containing loops in unactivated cells. Despite relatively short activation times used in our experiments, we also identified many “indirect” targets of NF-κB. These genes appeared to be regulated by NF-κB-induced transcription factors and thereby represented downstream effects of NF-κB activation. Consequently, transcriptional responses of such genes were delayed compared to direct NF-κB target genes. Taken together, our studies define the first steps of an NF-κB-responsive inducible gene cascade in activated B cells and highlight mechanisms by which kinetic patterns of NF-κB-dependent gene induction are established.

## Results

The availability of well-curated lists of NF-κB responsive genes and their time-dependent expression in response to activating stimuli is an essential prerequisite to elucidate transcriptional consequences of NF-κB activation. Because functional characterization of NF-κB responses in lymphocytes is especially scarce, we initiated studies to understand the kinetics of NF-κB-dependent inducible gene transcription in B lymphoid cells activated via the pharmacological equivalent of B cell antigen receptor signaling.

### Inducible transcriptional response in B lymphoid cells

**Fig 1:**
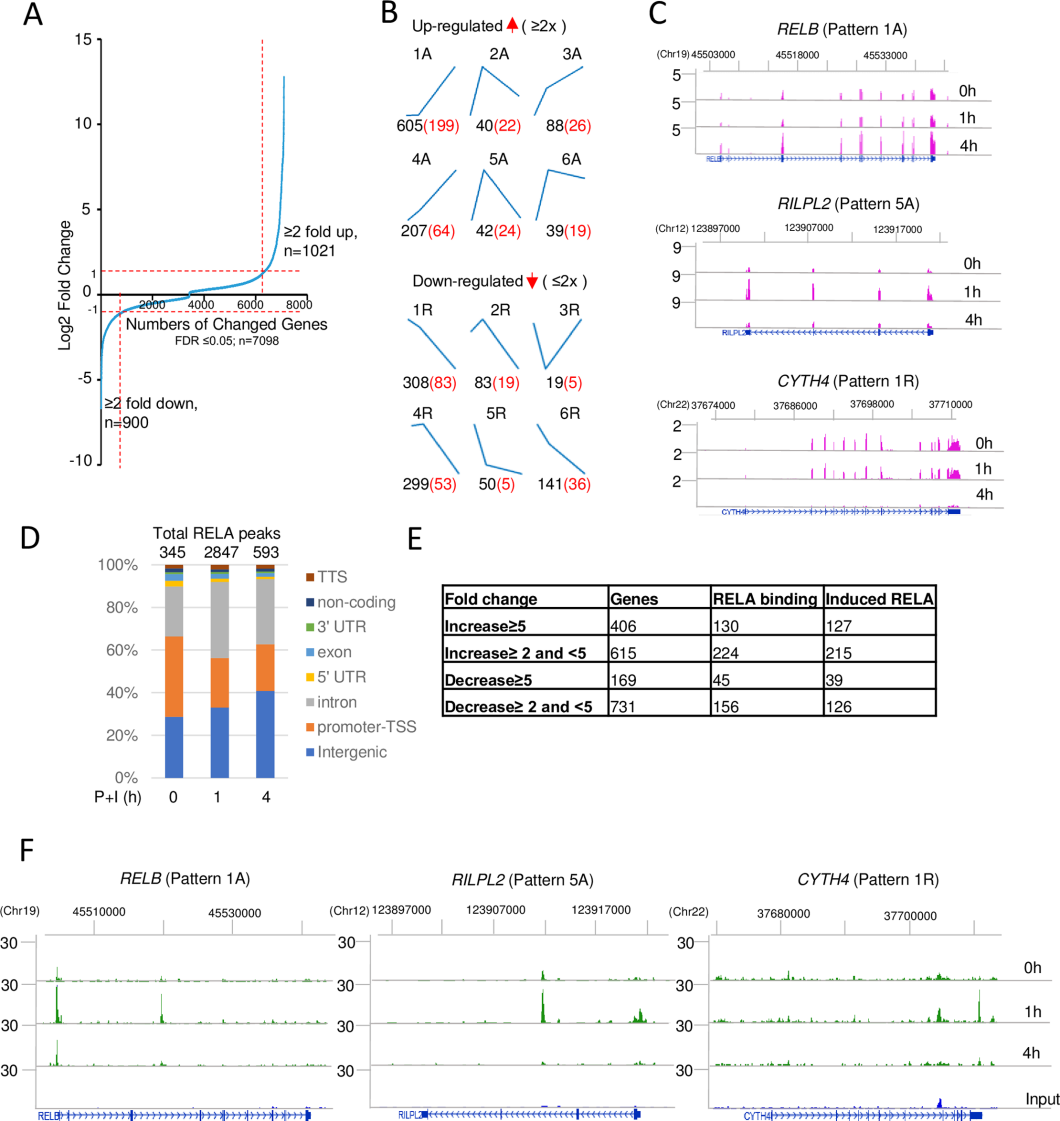
Inducible transcription and RELA binding in activated B lymphoma cells. BJAB human B lymphoma cells were treated with P+I for 1 or 4 h, followed by analyses of inducible transcription and RELA binding by RNA-Seq and ChIP-Seq, respectively. (A) Gene expression changes at either 1 or 4 h post activation compared to unactivated cells. Two biological replicate experiments were carried out, and differential gene expression was analyzed using EBSeq. (B) Inducibly up- or down-regulated transcripts were partitioned into 6 patterns (middle panel, top, or bottom, respectively) by *k*-means clustering (see also S1B–S1E Fig). Each graph represents the gene expression pattern at 0, 1, and 4 h. Numbers of genes in each pattern are indicated below in black. The letter “A” after a pattern number indicates genes that are activated by P+I, and the letter “R” indicates genes that are repressed by P+I. Red numbers identify putative RELA target genes according to criteria discussed in the text and summarized in parts D and E below. (C) Representative examples of RNA-Seq profile of genes corresponding to patterns 1A and 5A of up-regulated genes (right panel, top, and middle) and from pattern 1R of down-regulated genes (right panel, bottom). The *y* axis represents normalized reads per million. Chromosomal locations of genes in hg19 are shown above RNA-Seq tracks. (D) Two replicate RELA ChIP-Seq experiments were carried out as described in the Methods section. Peaks were called using MACS2 with input DNA as control at FDR ≤ 0.05. Peaks with peak score ≥ 100 that were common to both biological replicates were used for further analysis. ChIP-Seq experiments were annotated with respect to genomic location using HOMER. Total numbers of RELA peaks are noted above the bars, and activation times are indicated below. (E) Relationship of inducible transcription and RELA binding. Genes were categorized based on the level of inducible activation (top 2 rows) or repression (bottom 2 rows). Genes in each category (second column) that bound RELA (third column) were assigned based on criteria noted in the text. The majority of RELA binding was induced upon cell activation (fourth column). Newly identified RELA-bound up- and down-regulated genes are listed in S1 Table. (F) Representative browser tracks of RELA binding to genes whose transcriptional responses are shown in part C. The *y* axis represents normalized reads per 10 million. Chromosomal locations of each gene in hg19 are shown above ChIP-Seq tracks. RNA-Seq and ChIP-Seq data are available on the GEO website (http://www.ncbi.nlm.nih.gov/geo/) (Accession number GSE117259). ChIP-Seq, chromatin immunoprecipitation and sequencing; FDR, false discovery rate; GEO, Gene Expression Omnibus; P+I, phorbol 12-myristate 13-acetate and ionomycin; RNA-Seq, RNA sequencing; TSS, transcription start site; TTS, transcription termination site; UTR, untranslated region.

NF-κB inducibility in BJAB human B lymphoma cells closely paralleled that seen in primary B cells activated via the B cell receptor (BCR) (S1A Fig) [26]. Hallmarks of this response were rapid nuclear translocation of RELA followed by exit from the nucleus within 4–6 h after stimulation. Over a 4 h time course of phorbol 12-myristate 13-acetate (PMA) and ionomycin (P+I) treatment, approximately 1,000 genes were up-regulated and 1,000 genes were down-regulated more than 2-fold in BJAB cells (Fig 1A). We used *k*-means clustering based on correlation as the distance metric to partition up- and down-regulated genes into 6 distinguishable patterns (Fig 1B, S1B Fig). Hierarchical clustering of RNA expression profiles also revealed categories similar to those determined by *k*-means analysis (S1C and S1D Fig). Additionally, Gene Ontology (GO) analyses revealed distinct biological functions of genes in each category of up-regulated genes (S1E Fig). Expression of the largest subset of genes increased over the 4 h time course (Fig 1B, patterns 1A, 3A, and 4A). Smaller subsets of genes were rapidly up-regulated at 1 h and then either leveled off at 4 h or were subsequently down-regulated at 4 h (Fig 1B, patterns 2A, 5A, and 6A). Analogously, most genes that were down-regulated by P+I decreased continuously from 0 to 4 h. Examples from these categories are shown in Fig 1C. Amongst these diverse patterns of altered expression, our goal was to identify genes that were responding directly to NF-κB activation.

### NF-κB-dependent transcriptional responses

To identify genes that bound inducible NF-κB, we carried out ChIP-Seq using anti-RelA antibodies with unactivated cells or cells activated with P+I for 1 or 4 h. We focused only on those ChIP-Seq peaks that exceeded a threshold peak score of 100 (after peak annotation in HOMER) and were replicated in 2 independent ChIP-Seq experiments (S1F Fig). We reasoned that these stringent criteria would increase focus on robust RELA interactions genome-wide, despite decreasing the total numbers of peaks being studied. We identified 345 RELA binding sites prior to cell activation. The number of RELA-bound sites increased to nearly 3,000 after 1 h of activation and thereafter fell back to approximately 600 sites at 4 h (Fig 1D), demonstrating that genome-bound RELA closely paralleled total nuclear RELA levels. Sequences related to the κB motif (recognition site of NF-κB) were enriched at sites of RELA binding in all conditions (S1G Fig). Most inducible RELA binding at 1 h occurred in parts of the genome annotated as introns and intergenic regions (Fig 1D), a tendency that was noted in previous studies. We identified approximately 500 gene promoters that were newly targeted by RELA in activated cells.

As a first step towards identifying functional targets of RELA, we used HOMER to associate RELA peaks with genes. Peaks that were located outside annotated gene promoters and introns usually fell within 50 kb of the transcriptional start sites (TSSs) of assigned genes [20]. Applying these criteria to the approximately 1,000 genes whose RNA levels increased ≥2-fold with activation, we found inducible RELA bound to 354 genes; conversely, RELA was associated with 201 (out of 900) genes whose expression decreased ≥2-fold upon activation (Fig 1E). The majority of RELA-binding up-regulated genes increased in expression between 1 and 4 h of activation (Fig 1B, indicated in red). Conversely, expression of most RELA-binding down-regulated genes also decreased between 1 and 4 h, the period during which nuclear RELA levels were falling. Within the group of RELA-binding up-regulated genes were recognizable NF-κB target genes (such as *NFKBIA*, *TNFAIP3*, and *RELB)*, as well as others that had not been previously associated with NF-κB (such as *RILPL2*) (Fig 1F).

The 354 up-regulated and 201 down-regulated genes (such as *CYTH4*, Fig 1F) with inducible RELA binding constituted a working list of putative NF-κB target genes in activated B cells. Only 106 out of 354 up-regulated genes and 36 of 201 down-regulated RELA-binding genes identified in our analysis were present in a list of 1,992 putative NF-κB-responsive genes compiled from the “NF-κB Target Genes” list maintained by Thomas Gilmore’s lab (http://www.bu.edu/nf-kb/gene-resources/target-genes/), NF-κB target gene sets (https://www.yumpu.com/en/document/view/8327926/the-nfkb-target-gene-sets-are-listed-below-broad-institute), and other recent publications [24,27]. The newly identified 248 up-regulated and 165 down-regulated putative NF-κB targets are shown in S1 Table.

**Fig 2:**
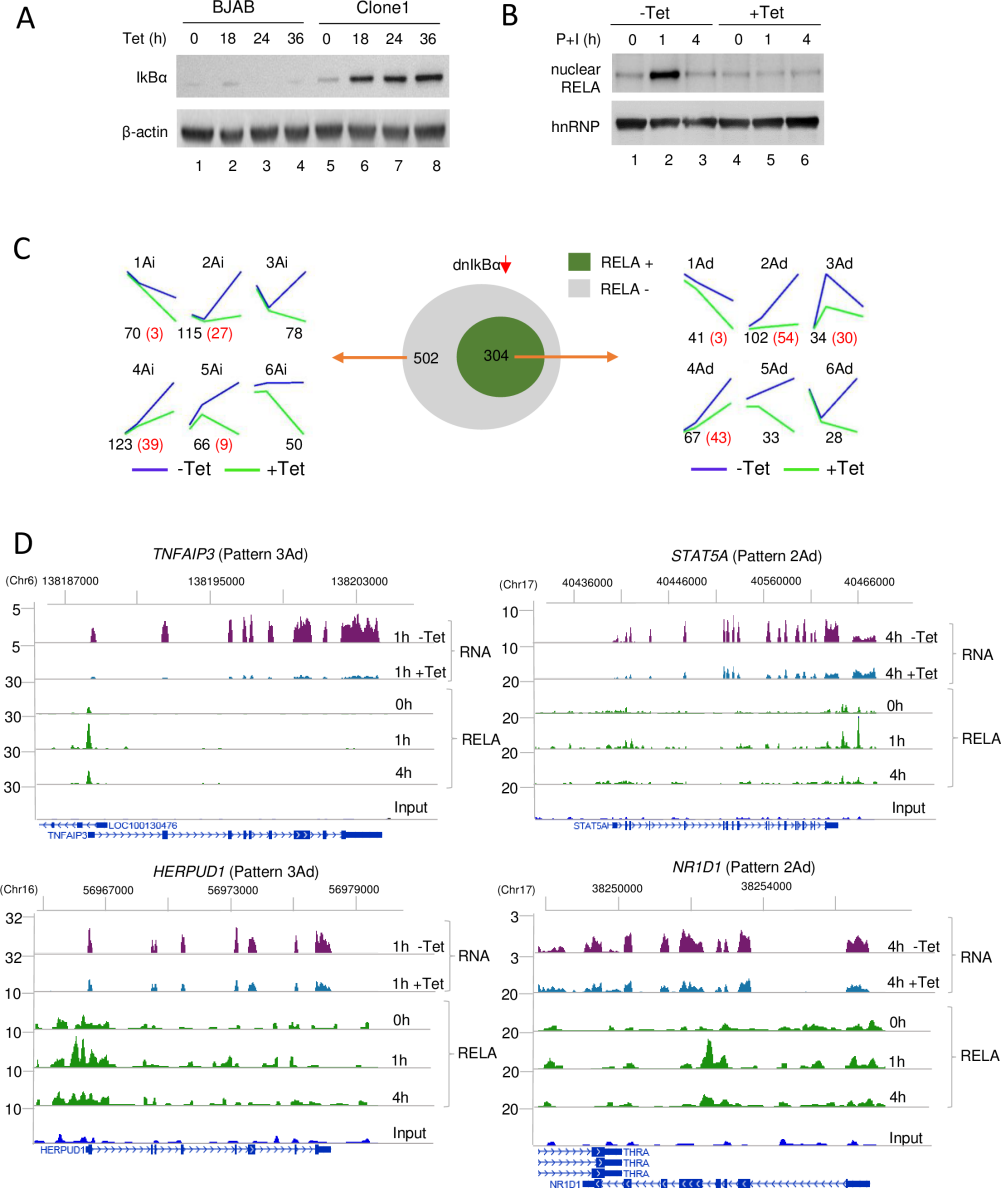
Attenuation of classical NF-κB-dependent transcription by dnIκBα expression. Two clones of BJAB cells were generated to perturb canonical NF-κB signaling by Tet-inducible dnIκBα expression. (A) Western blot analysis of parent BJAB cells and 1 clone treated with Tet for the indicated times (h); whole-cell extracts were fractionated by SDS-PAGE and transferred to NC membranes, which were probed with anti-IκBα or anti-β-actin (loading control) antibodies. (B) RELA induction in dnIκBα-expressing BJAB cells. Clone 1 cells were activated with P+I for 1 or 4 h either in the absence of Tet (-Tet) or after Tet pretreatment for 24 h (+Tet). Nuclear extracts were fractionated with SDS-PAGE, transferred to membranes, and probed with anti-RELA or anti-hnRNP antibodies (loading control). Results with 1 representative clone are shown. (C) Relationship of dnIκBα-responsive transcripts to RELA binding. Total RNA isolated from each BJAB clone activated in the presence or absence of Tet was subject to RNA-Seq (2 biological replicates from each clone). Genes that were differentially expressed in each clone because of dnIκBα expression were identified based on FDR ≤ 0.05 in EBSeq. Inducible activation of 806 genes was reduced by dnIκBα expression in both clones; of these, 304 bound RELA (middle panel, green), and 502 (middle panel, gray) did not bind RELA. Time-dependent expression patterns of RELA-binding genes in the absence (blue lines) or presence (green lines) of Tet-induced dnIκBα were determined by *k*-means clustering (right). The letters “Ad” after the pattern number indicate direct target genes that were activated by RELA, and the letters “Ai” indicate indirect target genes that were RELA dependent. Numbers of genes in each category are shown in black below the graphs. Numbers in red identify genes that were changed more than 2-fold in the absence of Tet. Time-dependent expression patterns of genes that were down-regulated by IκBα but did not bind RELA are shown to the left. Numbers and color coding are as described above. (D) Representative RNA-Seq and ChIP-Seq tracks for RELA target genes identified in C (green). Two previously known NF-κB target genes (*TNFAIP3* and *STAT5*) with different expression kinetics are shown on the top line, and 2 newly identified RELA target genes (*HERPUD1* and *NR1D1*) are shown on the bottom line. RNA-Seq tracks show the effects of dnIκBα expression (+Tet) at the time of maximum expression (1 h for pattern 3Ad and 4 h for pattern 2Ad). RNA-Seq tracks for all time points +/− Tet are provided in S2G Fig. All time points are shown for RELA ChIP-Seq tracks to visualize the dynamics of RELA recruitment and loss from the genome over the experimental time course. Numbers above the tracks refer to gene location in hg19. A complete list of all 304 RELA target genes identified in this study is provided in S2 Table. Genome scale datasets are available on the GEO website (http://www.ncbi.nlm.nih.gov/geo/) (Accession number GSE117259). ChIP-Seq, chromatin immunoprecipitation and sequencing; dnIκBα, dominant negative mutated IκBα; FDR, false discovery rate; GEO, Gene Expression Omnibus; hnRNP, heterogeneous nuclear ribonucleoprotein; IκBα, NFKB inhibitor alpha; NC, nitrocellulose; NF-κB, nuclear factor kappa B; P+I, phorbol 12-myristate 13-acetate and ionomycin; RNA-Seq, RNA sequencing; Tet, tetracycline.

To directly identify functional targets of inducible RELA (that is, genes whose transcriptional changes depended on RELA binding), we attenuated classical NF-κB activation by expressing a degradation-resistant, mutated dominant negative IκBα (dnIκBα) [28,29]. This form of IκBα is expected to quench the release of NF-κB proteins from all cytosolic IκBs via the posttranslational pathway. For this, we generated 2 clones of BJAB cells in which dnIκBα could be induced by tetracycline (Tet) treatment (Fig 2A). In these clones, nuclear RELA induction in response to P+I was similar to that of control BJAB cells in the absence of Tet but was abolished in cells that had been pretreated with Tet for 24 h (Fig 2B). To determine the effects of dnIκBα expression on inducible gene expression, each clone was either pretreated with Tet for 24 h (to induce dnIκBα) or not, followed by activation with P+I for 0, 1, or 4 h. Replicate experiments were carried out with each clone, and total RNA was prepared for RNA-Seq. Basal gene expression was not affected substantially in the presence or absence of Tet (S2A and S2B Fig). We compared inducible gene expression in each clone in the presence or absence of Tet and focused only on those inducible genes whose response to dnIκBα was replicated in both clones.

We identified 806 genes whose inducible expression was significantly reduced (false discovery rate [FDR] ≤ 0.05) by dnIκBα at either 1 or 4 h (Fig 2C, middle) in both Tet-inducible clones. Of these, 304 genes inducibly bound RELA in our ChIP-Seq experiments and were therefore considered to be direct transcriptional targets of RELA. These genes varied in their kinetic responses to cell activation (Fig 2C, right and S2C Fig) and were enriched for NF-κB binding motifs at sites of RELA binding as well as in their promoters (S2D Fig). We found that NF-κB target genes with different kinetic expression patterns enriched for different biological functions (S2F Fig). GO analyses showed that transiently activated genes (pattern 3Ad) were associated with processes such as “negative regulation of cellular processes,” “leukocyte activation,” and “inflammatory response.” By contrast, processes that scored high among genes whose expression continued to increase between 1 and 4 h activation (patterns 2Ad and 4Ad) included “ribosome biogenesis,” “immune response,” regulation of “Type 1 interferon production,” and “cellular response to cytokines.” These observations indicate that kinetic patterns were associated with distinct functional categories of NF-κB target genes. Additionally, de novo motif analysis in HOMER revealed distinct transcription factor motifs associated with RELA peaks in different kinetic patterns. RELA peaks in patterns 2Ad and 4Ad genes were enriched for the κB motif as well as the motif for transcription factor AP1, whereas the latter motif was not evident in RELA peaks of pattern 3Ad genes (S2E Fig). This list of 304 NF-κB target genes included many that had not been previously identified as being NF-κB responsive (S2 Table).

### Features of the NF-κB response

To probe the NF-κB response, we focused on 130 of the 304 direct target genes that were induced more than 2-fold in the absence of Tet (S2 Table). Out of these 130 genes, 74 have not been previously categorized as NF-κB targets. We found that virtually all transiently induced genes were amongst these 130 most robustly induced, RELA-binding, and dnIκBα-sensitive genes (Fig 2C, right, red numbers). This category included genes such as *TNFAIP3* and *HERPUD1* (Fig 2D, left). The prevalence of genes with this expression profile was consistent with reports showing that many NF-κB target genes have short-lived mRNAs [30]. For the majority of these genes, RELA binding occurred close to TSSs (S4D Fig). Second, most (95 out of 130) of these genes were contained in the set of 354 putative target genes identified in Fig 1. The remaining 259 (out of 354) genes that were not substantially affected by dnIκBα, despite robust inducible RELA binding and altered gene transcription, reaffirmed the idea that inducible RELA binding and inducible transcription are insufficient criteria to identify functional targets of NF-κB. Third, mRNA levels of many genes continued to increase within the time frame of our studies (Fig 2C, right, patterns 2Ad and 4Ad). These included genes such as *STAT5A* and *NR1D1* (Fig 2D, right). We found that RELA was recruited to these genes at 1 h, but most of it was lost by 4 h. These observations indicated that continued increase in mRNA was RELA-independent, suggesting that RELA was required to initiate but not maintain transcription of these genes.

**Fig 3:**
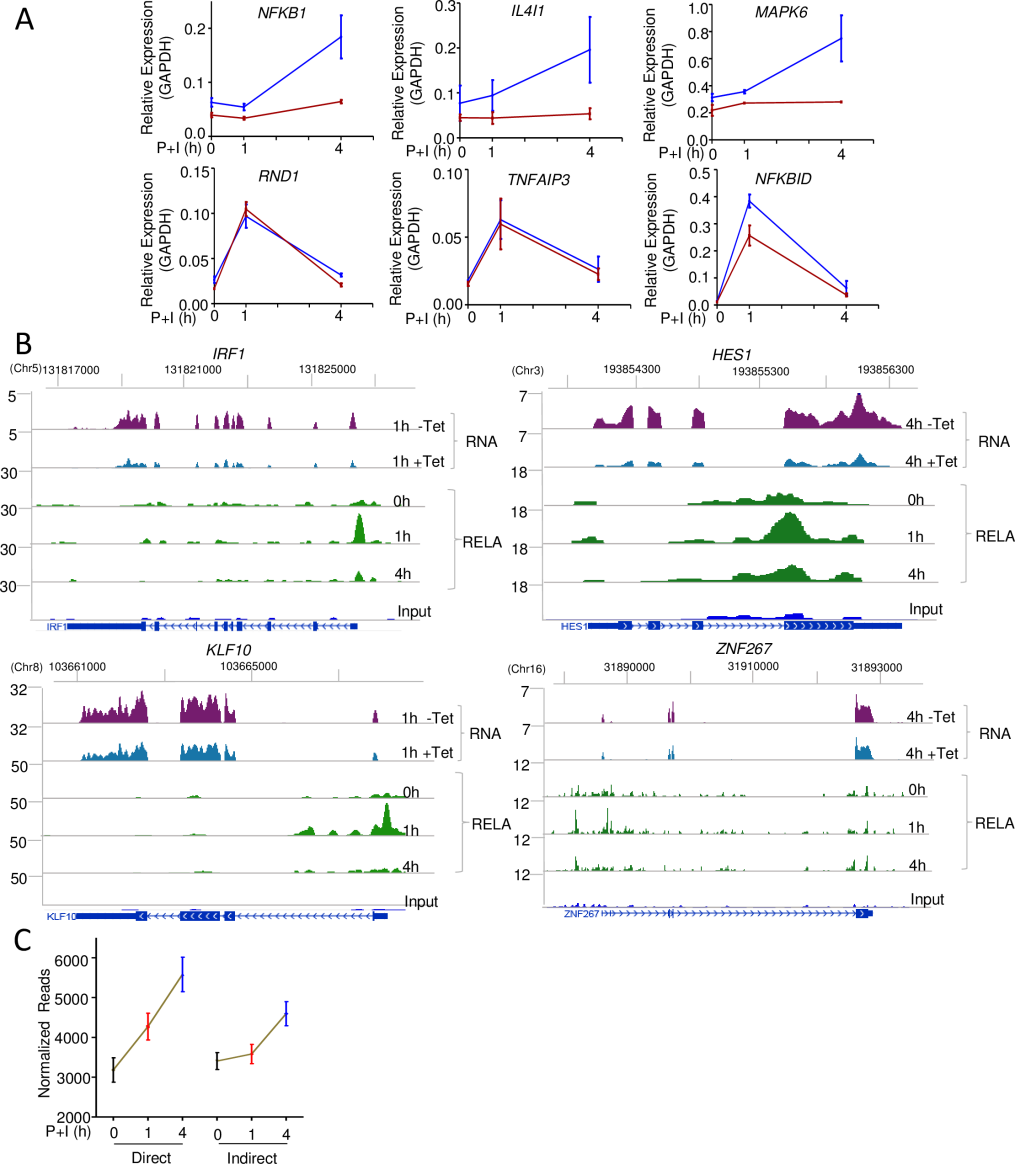
Modes of transcriptional activation by RELA. (A) Selected RELA target genes were assayed for effects of ERK inhibition on inducible transcription. qRT-PCR was carried out using RNA extracted from BJAB cells activated with P+I for indicated times in the absence (blue lines) or presence (red lines) of PD0325901. The top line shows representative genes whose maximal activation occurs between 1 and 4 h, during the period when nuclear RELA levels diminish. The bottom line shows representative genes that are transiently induced by P+I; data shown are the average of 2 independent experiments with qRT-PCR carried out in duplicate; error bars represent the standard error of the mean between experiments. (B) Representative examples of transcription factors identified as direct RELA targets in activated BJAB cells. Two lines at the top of each panel show RNA-Seq tracks obtained from cells that were pretreated with Tet (+Tet) or not (-Tet) for 24 h, followed by activation with P+I. Only time points at which RNA expression changed maximally are shown; complete time courses for each gene are provided in S3H Fig. *Y* axis numbers denote normalized reads per million. The next 4 lines show representative RELA ChIP-Seq tracks over the entire time course. Peak calling was carried out with MACS2, using input DNA as the control. Transcription orientation (arrows) and gene organization in hg19 are noted below each set. The *y* axis denotes normalized reads per 10 million. ChIP followed by quantitative PCR validation and NF-κB dependence of these genes is shown in S3E Fig. *IRF* and *KLF* family members have been previously proposed as NF-κB target genes. *HES1* and *ZNF267* were identified in this study. (C) Kinetic patterns of gene induction of direct (304) and indirect (502) RELA target genes in activated BJAB cells. Cells were activated with P+I for the indicated times, followed by RNA-Seq. Mean normalized reads for direct and indirect target genes from 2 independent experiments are plotted for each time point. Error bars represent the standard error of the mean. Genome scale datasets are available on the GEO website (http://www.ncbi.nlm.nih.gov/geo/) (Accession number GSE117259). Underlying data for Fig 3A and C are provided in S1A and S1B Data. ChIP-Seq, chromatin immunoprecipitation and sequencing; ERK, extracellular signal–regulated kinase; GAPDH, glyceraldehyde 3-phosphate dehydrogenase; GEO, Gene Expression Omnibus; NF-κB, nuclear factor kappa B; P+I, phorbol 12-myristate 13-acetate and ionomycin; qRT-PCR, quantitative real-time PCR; RNA-Seq, RNA sequencing; Tet, tetracycline.

To further explore the basis for continued transcription of NF-κB target genes after chromatin-bound RELA was depleted, we drew upon the observation that the AP1 motif was enriched in RELA peaks associated with genes whose expression levels continued to increase between 1 and 4 h (patterns 2Ad and 4Ad, S2E Fig). We tested the possible involvement of this transcription factor family by pharmacologically inhibiting ERK, a mitogen-activated protein kinase (MAPK) required for activation of AP1-like factors. RNA isolated from BJAB cells treated with P+I in the presence or absence of the ERK inhibitor PD0325901 was assayed by quantitative real-time PCR (qRT-PCR) for expression of genes from patterns 2Ad and 4Ad. We found that inducible expression of 3 genes from these categories was suppressed by ERK inhibition (Fig 3A, top line). To rule out that PD0325901 affected NF-κB activation by some unanticipated pathway, we also assayed genes whose expression kinetics coincided with RELA induction by P+I (pattern 3Ad in Fig 2C). These genes were not substantially affected by PD0325901 (Fig 3A, lower line). We conclude that ERK-dependent transcription factors confer continued transcriptional activity to a subset of NF-κB target genes after induced nuclear RELA levels dissipate. The kinetically delayed response of these genes is consistent with a “priming” role for RELA, followed by an activator role for AP1-like factors. Such priming may involve recruitment or stabilization of additional transcription factors or coactivators by transiently bound RELA.

### Secondary effects of NF-κB activation

RELA did not bind the remaining 502 genes whose expression was reduced by dnIκBα in both clones (Fig 2C, middle, gray). Transcriptional responses of these genes in the presence or absence of dnIκBα also clustered into patterns similar to those seen for direct target genes, including 2 in which gene expression was inducibly down-regulated upon cell activation (Fig 2C left, S3A Fig). A pattern that was prominently missing in this gene set compared to direct RELA targets was one in which RNA levels increased transiently in response to activation. To understand the basis for these genes being affected by dnIκBα in the absence of RELA binding, we looked for shared transcription regulatory features in this set. HOMER analysis of promoter regions of these genes revealed an enrichment for the binding motif of transcription factor MYC (S3B Fig). The MYC motif was found in the promoters of 247 of these 502 genes, including *BRIXI*, *DDX18*, and *AKAP1*, and the majority of these genes (226 out of 247) were previously shown to bind MYC in ChIP-Seq assays [31]. Because *MYC* is a known target of NF-κB (S3C Fig), we hypothesized that this set of genes was induced by NF-κB-activated transcription factors. Among 304 direct RELA target genes, we found 37 that encoded DNA-binding transcriptional regulators (S3D Fig, left). These included genes for *KLF10* and *IRF1* that were previously linked to NF-κB. We also found many other genes encoding factors such as *HES1* and *ZNF267* that had not been previously associated with NF-κB (Fig 3B). We confirmed dnIκBα-sensitive RELA recruitment to promoters of these genes by chromatin immunoprecipitation (ChIP) followed by quantitative PCR (qPCR) (S3E Fig). Thus, many transcription factor genes were induced in activated cells via NF-κB. To further substantiate the hypothesis that NF-κB-induced transcription factors contributed to dnIκBα-sensitive gene expression, we evaluated whether promoters of indirect NF-κB target genes contained recognition motifs for NF-κB-regulated transcription factors.

For the promoter analysis, we focused on 78 (out of 502) genes (S3 Table) that were dnIκBα-sensitive, did not bind RELA, and were changed more than 2-fold by P+I treatment in the absence of Tet. We searched for transcription factor motifs that were present in more than 20% of these promoters (S3F Fig). This analysis revealed recognition sites for kruppel-like factor (KLF), zinc finger protein (ZNF), and ETS-domain transcription factors. The correspondence between transcription factor genes induced by NF-κB and motifs enriched in promoters of indirect NF-κB target genes supports the notion that the set of dnIκBα-sensitive genes that did not bind RELA were targets of transcription factors activated by NF-κB. However, in the present study, we did not directly evaluate binding of such NF-κB-induced transcription factors genome-wide. We will refer to such genes as indirect targets of RELA. In keeping with their proposed dependence on NF-κB-induced transcription factors, RNA levels of such indirect targets increased at later times compared to direct targets (Fig 3C).

GO analyses of the dominant patterns (2Ai and 4Ai) of indirect NF-κB target genes showed that they were enriched for genes involved in RNA processing, ribosome biogenesis, and RNA-associated metabolic processes (S3G Fig). These processes were largely distinct from those associated with direct NF-κB target genes, thereby identifying a hierarchy of biological consequences associated with NF-κB activation. Because MYC has been implicated in activating ribosomal genes [32], we surmise that many of the identified processes are the consequence of NF-κB-directed MYC expression in activated BJAB cells. Taken together, our kinetic analyses identified gene targets at which inducible RELA binding activated transcription (direct targets) and, additionally, revealed secondary transcriptional consequences of NF-κB activation in B lymphoblastoid cells via NF-κB-induced transcription factors (indirect targets). We also uncovered a mechanism by which RELA induced persistent transcriptional activity despite its transient nuclear induction.

### Transcripts up-regulated by dnIκBα

**Fig 4:**
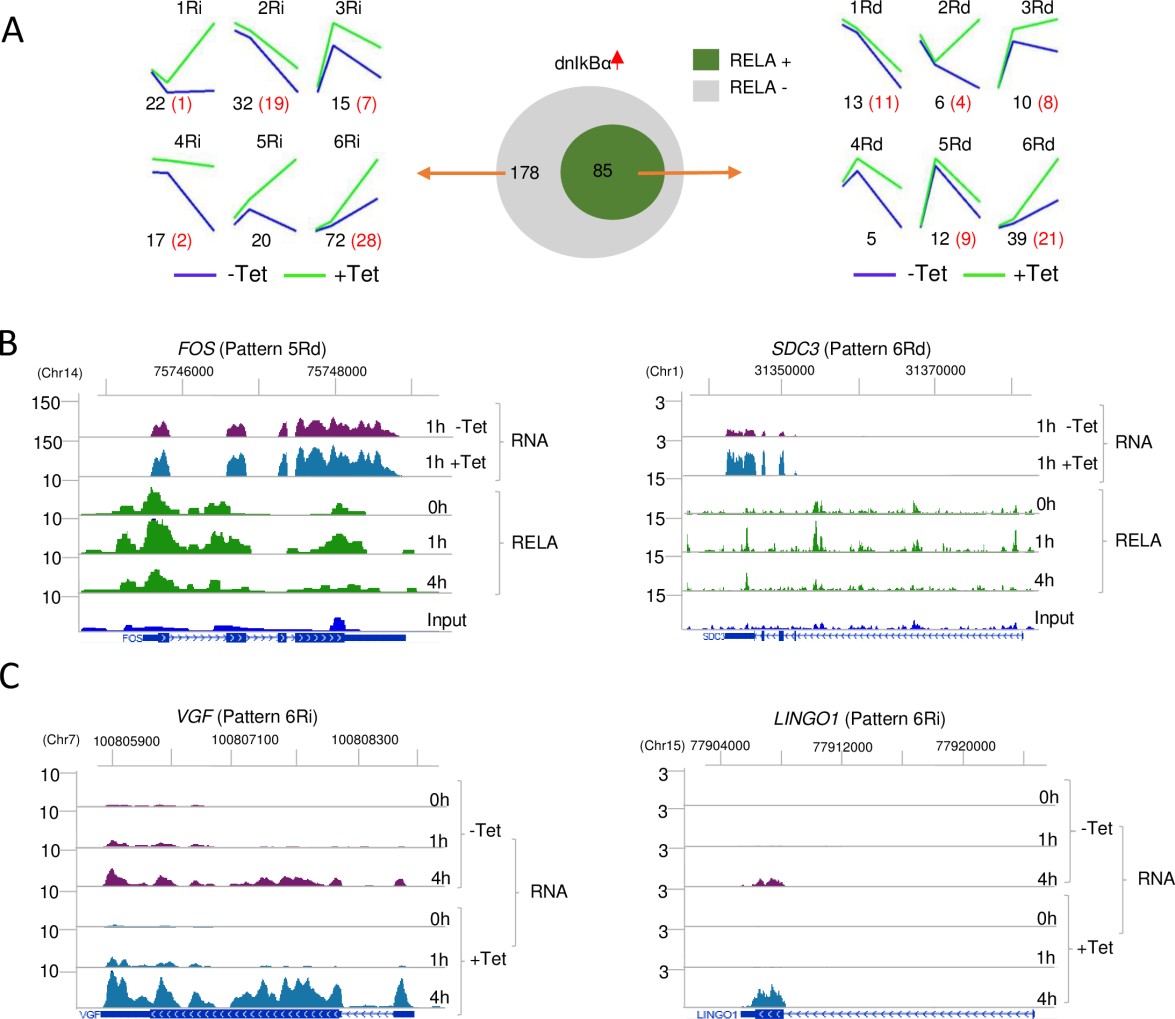
Repression of gene expression by NF-κB. Genes whose expression was increased by dnIκBα in both Tet-responsive clones were identified by RNA-Seq. (A) Genes that bound RELA (middle, green) and their time-dependent expression patterns in the absence (blue) or presence (green) of Tet-induced dnIκBα are shown on the right. See also S4A Fig. The letters “Rd” after a pattern number indicate genes that were down-regulated by RELA binding, and the letters “Ri” indicate genes whose down-regulation was RELA dependent but did not bind RELA. Numbers of genes in each category are shown in black below the graphs. Red numbers correspond to genes whose expression changed more than 2-fold in the absence of Tet. Expression patterns of 178 dnIκBα-up-regulated genes that did not bind RELA (middle, gray) are shown on the left, with numbers and color coding as described above. (B) Representative examples of dnIκBα-up-regulated genes that bind RELA. Top 2 tracks show RNA-Seq tracks in the presence or absence of Tet-induced dnIκBα at the time of maximal RNA expression; the *y* axis denotes normalized reads per million. Complete time courses for each gene are provided in S4B Fig. The bottom part shows RELA ChIP-Seq tracks over the entire time course in activated BJAB cells. Genomic organization and transcription orientation (arrows) are shown below the input DNA track. Numbers above the tracks refer to gene location in hg19. The *y* axis denotes normalized reads per 10 million. (C) Representative examples of dnIκBα-up-regulated genes that do not bind RELA. These genes are proposed to be modulated by RELA-responsive factors (see text). RNA-Seq tracks over the complete activation time course in the absence (-Tet) or presence (+Tet) of dnIκBα are shown. Genome scale datasets are available on the GEO website (http://www.ncbi.nlm.nih.gov/geo/) (Accession number GSE117259). ChIP-Seq, chromatin immunoprecipitation and sequencing; dnIκBα, dominant negative NFKB inhibitor alpha; GEO, Gene Expression Omnibus; NF-κB, nuclear factor kappa B; RNA-Seq, RNA sequencing; Tet, tetracycline.

We identified 263 gene transcripts that were up-regulated by dnIκBα expression in both Tet-inducible BJAB clones. Eighty-five of these genes bound RELA in activated cells (Fig 4A, green circle); our interpretation is that RELA binding reduced expression of this subset of genes. Such RELA-repressed genes had diverse expression profiles, including genes that were up- or down-regulated in response to activation (Fig 4A right, patterns 6Rd and 1Rd, respectively, S4A Fig left). Some examples are shown in Fig 4B (see also S4B Fig for complete RNA time courses). Of the 53 (out of 85) RELA-repressed genes whose expression changed more than 2-fold in response to activation in the absence of Tet, 36 were not found in NF-κB-related databases and thus represent novel targets of NF-κB activity (S4 Table). In contrast to RELA-activated genes that contained canonical κB motifs within RELA peaks, sequence motifs underlying RELA peaks of RELA-repressed genes were enriched for AP1 binding sites (S4C Fig). Additionally, RELA binding was scattered throughout these genes rather than being enriched in promoter regions (S4D Fig). Our interpretation is that RELA was recruited to these regions primarily by association with DNA-bound AP1 factors rather than direct DNA binding by RELA itself. Such interactions may attenuate transcriptional activation by AP1, thereby resulting in gene repression.

In total, 178 genes up-regulated by dnIκBα expression did not bind RELA (Fig 4A, left; Fig 4C). We surmised that up-regulation of these genes by dnIκBα was also a secondary consequence of NF-κB activation. That is, such genes were either activated by transcription factors that were up-regulated by dnIκBα expression or attenuated by factors that were direct or indirect RELA targets. We found examples of each category in our RNA-Seq database. Among the 85 RELA-repressed genes, we found 23 that encoded transcriptional regulators (S3D Fig) whose increased expression in the presence of dnIκBa could be responsible for activating a subset of up-regulated genes that did not bind RELA. To further identify factors that indirectly activated gene transcription by dnIκBa, we screened promoter motifs present in 57 (out of 178) genes whose expression changed more than 2-fold in the absence of Tet. Most of these overlapped with motifs present in genes that were indirectly activated by NF-κB (S5 Table, S4E and S4F Fig). However, a few motifs were selectively associated with dnIκBα-activated genes, such as those for signal transducer and activator of transcription (STAT) and SRY-box (SOX) factors, and those for nuclear hormone receptors (S4F Fig). As shown above, *STAT5* is a direct target of NF-κB in these cells and may negatively regulate a subset of these indirect RELA-repressed genes. We also found *SOX8* and *PPARG* mRNAs to be up-regulated by P+I in dnIκBα-expressing cells (S4G Fig); however, the associated mechanism(s) have not been further addressed in this study. We conclude that NF-κB activation also initiates a cascade of transcriptional down-regulation, both by directly interacting with a subset of genes and by modulating expression of other transcription factors.

GO analysis of RELA-repressed genes revealed some interesting features. As noted for RELA-activated genes, there was relatively little overlap between the 6 patterns for the top 10 biological processes (S4H Fig). Among genes that were directly repressed by RELA, we found 1 pattern (6Rd) to enrich for genes involved in transcription termination by Pol II. One interpretation is that NF-κB proteins elevate gene expression by both activating transcription initiation and inhibiting transcription termination. The latter mechanism may apply to genes for which NF-κB has been proposed to push prebound RNA polymerase from abortive initiation state to productive elongation mode. Other pathways that featured in this gene set included modulation of biosynthetic and metabolic processes and negative regulation of cellular processes. Prominent among genes that were indirectly repressed by RELA were those involved in autophagosome organization and assembly and posttranslational protein modifications (S4I Fig). By up-regulating essential autophagy genes such as ATG5 and 7 [33] and suppressing others involved in autophagosome assembly, NF-κB may fine-tune the autophagic response. The emerging patterns reveal synergistic use of RELA-dependent activation and suppression of gene expression to optimize cellular responses.

### RELA recruitment to nonfunctional sites

The preceding analysis started by identifying genes whose inducible expression changed significantly in the presence of dnIκBα. While focusing on functional targets of RELA, this approach did not fully utilize our time-dependent RNA analyses. In particular, we missed out on the broader genomic landscape of NF-κB recruitment in response to B cell activation, especially where RELA binding did not affect mRNA levels after dnIκBα induction. Such sites were of potential interest because they vastly outnumbered those where RELA binding had functional consequences and may therefore contribute to B cell biology in unanticipated ways. We did this in 2 steps. First, we identified genes whose expression did not change in both dnIκBα-inducible clones across all time points of activation with or without Tet. Approximately 600 out of 8,000 such genes inducibly bound RELA (S5A Fig). These binding sites were associated with canonical κB and AP1 motifs (S5B and S5C Fig). Second, we used *k*-means clustering with correlation parameter to visualize gene expression patterns of the remaining genes in each clone in the absence or presence of dnIκBα (S5D Fig). Patterns with similar expression characteristics in both clones were combined into 4 patterns of inducible gene expression (S5E Fig).

Patterns I and II corresponded to genes whose expression decreased or increased, respectively, in response to dnIκBα expression. Analysis of these gene sets largely recapitulated the conclusions from Figs 2–4. Patterns III and IV provided insights into patterns of inducible expression that were not identified in the preceding analysis. These groups contained genes that were either up- (Pattern III) or down-regulated (Pattern IV) with activation but whose expression was not affected by dnIκBα (S5E and S5F Fig). Numerous genes in each set bound RELA (green circles), and NF-κB and AP1 binding sites were again enriched in sequences underlying RELA peaks (middle column). These observations reinforced the idea from Figs 1 and 2 that inducible binding coupled with transcriptional changes was an insufficient criterion to identify functional targets of transcription factors. Differential regulation of these gene sets was also evident from their promoter architecture. Binding motifs for ETS-domain proteins and the transcription factor Yin Yang 1 (YY1) were enriched in gene promoters that were up-regulated with activation (S5E Fig, right column), whereas the motif for IRFs was enriched weakly amongst genes that were down-regulated with activation.

### Preformed loops at RELA-dependent genes

Rapid gene induction in response to cell stimulation is programmed in different ways. In the classic example of *c-Fos* induction, phosphorylation of a promoter-bound transcription factor in unactivated cells triggers RNA synthesis after cell activation [34,35]. In other instances, RNA polymerases bound at promoters in unactivated cells can be pushed into elongation mode by phosphorylation of their C-terminal domain in response to stimuli [36,37]. This mode of activation has been implicated at some NF-κB-dependent target genes [38–40]. To gain more insight into inducible gene expression by RELA, we carried out ChIP-Seq with antibodies directed against Pol II. We used a threshold peak score of ≥100 in HOMER and reproducibility in replicate experiments to assign Pol II occupancy with confidence (S6A Fig). Using these criteria, we found that 50 out of 130 direct NF-κB target genes contained prebound Pol II at their promoters prior to activation (S6B Fig, S6A Table). However, these genes recruited additional Pol II after cell activation, which was evident in the average profile across all direct target genes (S6C and S6D Fig). Proportionally fewer indirect target genes (13 out of 78 genes) had prebound Pol II prior to P+I treatment, and inducible Pol II recruitment was clearly evident at these promoters in response to activation (S6E Fig). We conclude that Pol II recruitment is a major mechanism of inducible gene transcription by NF-κB.

**Fig 5:**
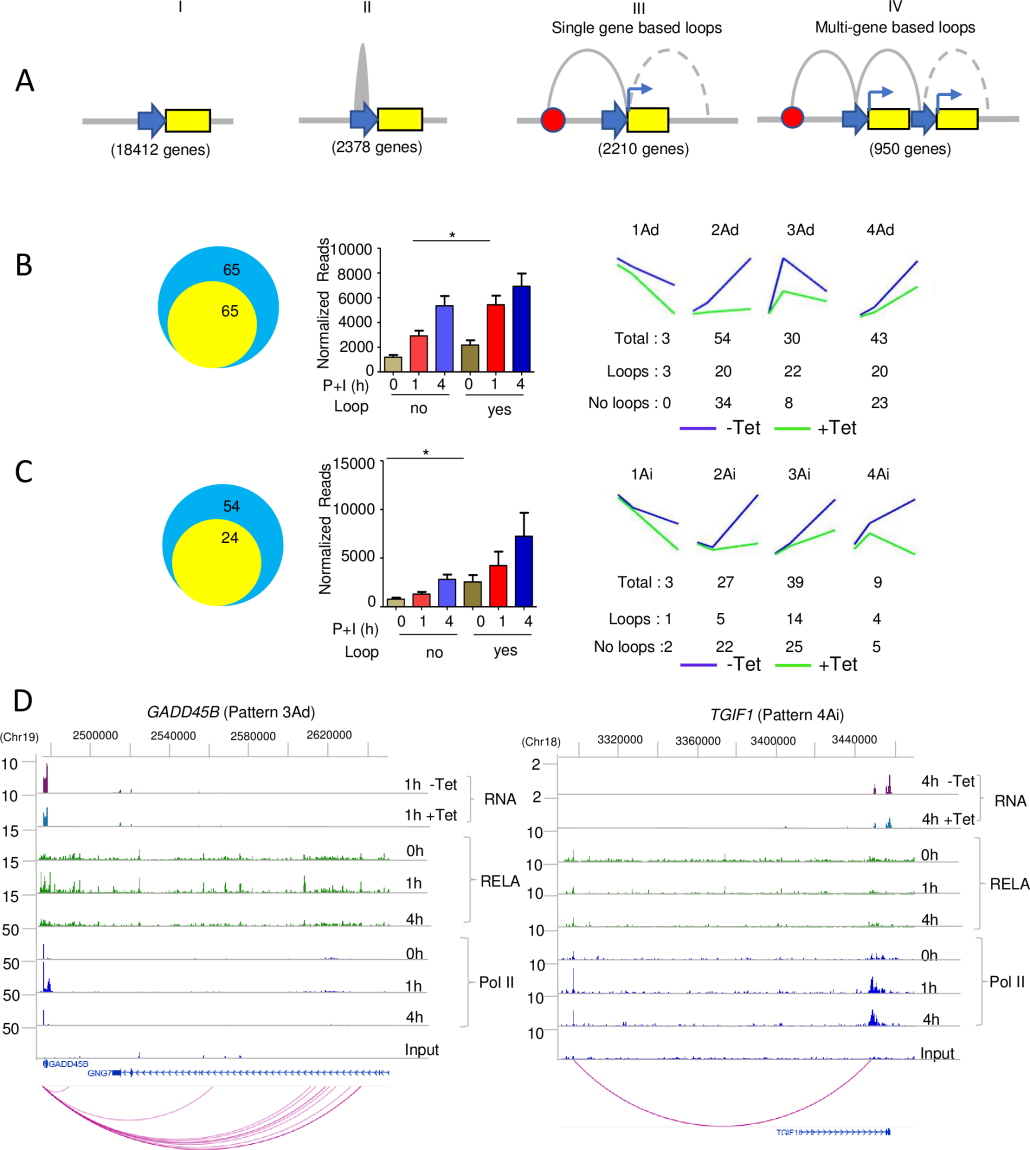
Kinetic patterning of NF-κB-dependent gene expression. (A) Relationship of Pol II–associated chromatin looping to NF-κB-dependent gene expression. Unactivated BJAB cells were used to carry out ChIA-PET after immunoprecipitation of cross-linked chromatin with anti-Pol II antibodies. Resulting sequence data were analyzed and categorized according to the type of promoter status. Category I = genes with no Pol II (no loops); II = genes with Pol II at promoter (no loops); III = genes with single-gene-based loops, in which only 1 gene was involved; IV = genes with multi-gene-based loops that included promoter–promoter interactions in which at least 2 genes were involved. (B) Analysis of 130 direct RELA target genes and (C) 78 “indirect” RELA target genes that are induced ≥2-fold by P+I. Yellow and blue circles denote genes that have or do not have Pol II–associated loops, respectively, in unactivated BJAB cells. Average expression profiles of genes within each group were obtained from RNA-Seq analysis of Tet-inducible BJAB clones (right). The letters “Ad” after a pattern number indicate target genes that were activated by RELA binding, and the letters “Ai” indicate target genes that were indirectly activated by RELA. Genes in each category are listed in S6B Table. (D) RELA and Pol II binding over the activation time course on representative direct and indirect RELA target genes. The effect of dnIκBα expression on RNA expression is shown on the top 2 lines, followed by RELA ChIP-Seq and Pol II ChIP-Seq tracks. The bottom line summarizes loops identified by ChIA-PET. Complete RNA time courses for each gene are shown in (S6J Fig). ChIA-PET data are available on the GEO website (http://www.ncbi.nlm.nih.gov/geo/) (Accession number GSE117259). Underlying data for Fig 5B and 5C are provided in S1C and S1D Data. ChIA-PET, chromatin interaction analysis by paired-end tag sequencing; ChIP-Seq, chromatin immunoprecipitation and sequencing; dnIκBα, dominant negative NFKB inhibitor alpha; GEO, Gene Expression Omnibus; NF-κB, nuclear factor kappa B; P+I, phorbol 12-myristate 13-acetate and ionomycin; Pol II, RNA polymerase II; RNA-Seq, RNA sequencing; Tet, tetracycline.

Presence or absence of Pol II at the basal state did not readily explain kinetic differences in patterns of NF-κB target gene induction. To further probe for a possible mechanism, we performed chromatin interaction analysis by paired-end tag sequencing (ChIA-PET) in cells prior to stimulation. This assay scores for interaction of Pol II–bound sequences with other parts of the genome [41,42]. Biological replicates were processed using ChIA-PET tool software [43], and the data were divided into 4 groups (Fig 5A). Over the entire dataset, genes that lacked Pol II had the lowest RNA levels at baseline, genes with Pol II–bound promoters with no loops had intermediate RNA levels, and genes whose Pol II–bound promoters engaged in looping interactions had the highest levels of RNA (S6F Fig). Similar trends were observed in previous ChIA-PET studies [42]. Additionally, we confirmed several looping interactions that had been identified in earlier studies, indicating that our assay scored for functionally relevant Pol II–associated interactions (S6G Fig).

We then examined chromatin interactions in the context of NF-κB target genes. Approximately half of the 130 robustly induced (≥2-fold) direct RELA target genes had Pol II loops in unactivated cells (Fig 5B, left; S6B Table). On average, RELA target genes with preformed loops reached close to maximum levels of inducible expression rapidly compared to those without loops (Fig 5B, middle), and most of the transiently induced target genes identified in Fig 2 (22 out of 30) fell in this category (Fig 5B, right, pattern 3Ad). Conversely, RELA target genes that did not have preformed loops were enriched for genes that were induced more slowly and whose expression increased continuously over the time course of activation (Fig 5B, right, 34 out of 54 genes in pattern 2Ad and 23 out of 43 genes in pattern 4Ad). One example of a prelooped RELA target gene is shown in Fig 5D (left).

For indirect target genes, the trends were reversed (Fig 5C). Approximately 30% of genes in this category (24 out of 78 robustly induced genes) had preformed Pol II loops (S6B Table). Looped genes in this set had higher basal RNA levels but did not show kinetic differences in RNA induction compared to nonlooped genes (Fig 5C, middle). Instead, indirect target genes that had preformed loops in unactivated cells achieved higher levels of induced RNA compared to genes with no loops. Many indirect target genes with preexisting loops tended to be induced early in the presence of dnIκBα but crashed thereafter (18 prelooped genes in patterns 3Ai, 4Ai in Fig 5C, right). One example of a prelooped indirect RELA target gene is shown in Fig 5D (right). We propose that preformed loops regulate kinetic patterns of direct RELA target genes, whereas they determine the maximal RNA output for indirect target genes.

While RELA target genes were typically involved in single promoter interactions (Fig 5A, pattern III), we also found approximately 1,000 genes that were involved in multiple promoter interactions (ChIA-PET category 4). These genes yielded GO terms such as “purine triphosphate metabolic process,” “pyrimidine nucleotide biosynthetic process,” and other comparable metabolic pathways (S6H Fig). Amongst these, we found interactions involving *PPP4C*, *ALDOA*, and histone genes (S6I Fig). It is likely that linking gene promoters via Pol II interactions provides a mechanism to coregulate genes that are involved in a common biological pathway [42]. In contrast, the need for greater flexibility in output of RELA-responsive genes depending on the stimulus and cell type may preclude their connection in a preformed network.

## Discussion

By combining time-dependent transcriptional responses with genome-wide recruitment of RELA and Pol II and perturbation of classical NF-κB activation, we sought to identify mechanisms by which kinetic patterns of NF-κB-dependent gene expression are established. In these studies, a pharmacologic equivalent of antigen receptor signaling was used to activate human B lymphoblastoid BJAB cells over a time course during which nuclear NF-κB was transiently induced. Three interesting features emerged from a consideration of RELA target genes. First, close to 60% of the 130 most robustly induced target genes identified here had not been previously connected with NF-κB responses. Our list also contained well-established NF-κB targets such as *MYC*, *TNFAIP3*, and *NFKBIA*, attesting to the validity of our analyses. The incompleteness of current lists of NF-κB target genes was further accentuated when we included the additional 174 targets identified here that were induced less robustly. Of these 174 genes, 80% were not present in NF-κB-related databases, while the remaining 20% included genes such as *TP53*, *TNIP1*, and *TAP1* that were previously linked to NF-κB. We surmise that NF-κB target genes identified here that are also present in earlier lists may represent more “universal” targets that respond regardless of cell type or stimulus. In contrast, genes uniquely identified in our study may represent cell type–or stimulus-specific responses. While use of a lymphoma cell line for these studies makes it difficult to draw direct connections to transcriptional responses of primary human B cells, the hundreds of new functionally curated NF-κB target genes identified here constitute a unique pool of possible mediators of NF-κB activity in B lymphoid cells.

We also identified many genes that we refer to as indirect NF-κB targets. Such genes were sensitive to dnIκBa expression but did not bind RELA. We hypothesize that such genes were activated (or repressed) by NF-κB-induced transcriptional regulators. Even within the relatively short time course of our kinetic studies, we identified 37 genes among the 304 direct targets that encoded transcriptional regulators. In addition to previously identified targets such as *MYC* and *IRF1*, this list included many new NF-κB-regulated factors such as hes family bHLH transcription factor 1 (HES1) and ZNF267. Additional studies are needed to directly evaluate the contribution of such factors in regulating indirect target gene transcription. It is possible that a subset of genes that we classified as indirect RELA targets are controlled by RELA bound to sites that did not score in the program used to connect binding sites to genes.

From the studies presented here, we cannot specify the subunit composition of RELA-containing homo- or heterodimers that activate transcription of the identified NF-κB target genes. Because most of the RELA genome binding occurred at 1 h after cell activation, our working hypothesis is that functional NF-κB measured in these assays was generated from cytosolic pools by the classical posttranslational pathway. By electrophoretic mobility shift assays, this form consists largely of p50/RELA heterodimers; however, sequential ChIP is required to verify this model. Additionally, the contribution of REL to dnIκBa-sensitive gene transcription was not experimentally evaluated by depleting REL in BJAB cells. Studies to address this question are in progress using *Rel*-deficient murine B cells.

Second, 2 dominant kinetic patterns of inducible expression emerged for the 130 genes that were most strongly induced. A small number (30 out of 130) were transiently induced and included genes such as *TNFAIP3* and *NFKBIA*. The expression pattern of such genes can be easily explained by transcriptional activation when bulk RELA is nuclear (at 1 h), followed by transcriptional inactivation when RELA moves back into the cytoplasm (at 4 h), together with rapid degradation of the encoded mRNAs. The short half-life of many of these transcripts has been previously highlighted [30]. More surprising was the observation that inducible expression of the majority of these genes (97 out of 130) continued to increase between 1 and 4 h of activation. This time period coincided with down-regulation of RELA from the nucleus, and indeed, we found that RELA was lost from gene promoters over this period. For a subset of genes that we tested, ERK activity was required for sustained RNA synthesis after loss of nuclear RELA, pointing to involvement of the AP1 family of transcription factors. Our working hypothesis is that RELA binding “primes” the promoter for subsequent binding and transcriptional activation by ERK-dependent transcription factors. However, continued RELA binding is not required for transcriptional activity, thus distinguishing this mode of gene regulation from synergistic promoter activity by cobound factors.

Our observations also provide a novel perspective on the phenomenon of assisted loading, a term used to describe cooperative recruitment of transcription factors that co-occupy gene regulatory sequences [44]. Regarding NF-κB/RelA, it has previously been shown that binding of IRF5 or STAT3 to a subset of genomic sites in activated hepatocytes or macrophages, respectively, requires simultaneous RELA activation [5,45]. In the examples presented here, we show instead that RELA does a hit-and-run on gene promoters that have a characteristic kinetic transcription profile. Although RELA is lost from these promoters, our experiments do not distinguish whether the κB site remains empty or is occupied by other factors. For example, p50 homodimers associated with IκBξ, which have been previously proposed to confer transcriptional activity [46], may substitute RELA-containing complexes at such sites. Alternatively, other proteins that recognize κB motifs may provide transcriptional activity in the absence of RELA. Nuclear factor of activated T cell (NFAT) proteins are a distinct possibility because they are induced in P+I-activated B cells [47], have been shown to bind to κB elements [48], and function in collaboration with AP1 factors.

Earlier studies have analyzed the connection between AP1/ERK and kinetics of gene induction by NF-κB. Natoli and colleagues showed that a subset of inflammatory gene promoters recruited RELA in response to LPS only after being marked by serine phosphorylated histone H3 (H3S10P) via p38 MAPK activity [49]. The 2-step process delayed RELA recruitment and transcriptional induction of these genes compared to other inflammatory genes to which RELA bound without requiring H3S10P. By contrast, RELA recruitment was not delayed even at late-induced genes in the studies described here, thereby invoking a novel mode of intersection between NF-κB and MAPK pathways. We note several differences that may underlie mechanistic variations observed for NF-κB-inducible transcription in the 2 studies. These include the different cell types used (dendritic cells versus B cells) that could differentially mark RELA recruitment sites, different initiating stimuli (LPS versus P+I) that induce distinct cytosolic signaling milieus, and the nature of NF-κB activation (sustained versus transient) that could influence gene expression outcomes. Further studies are needed to understand rules by which NF-κB tunes cellular responses to diverse stimuli.

In a more recent study, Brasier and colleagues identified AP1 and SP1 motifs in regions surrounding RELA peaks in TNFα-induced A549 (human pulmonary epithelial) cells [14]. Genes with SP1 motifs reached maximal inducible expression 30 min after activation, whereas those with AP1 motifs reached maximal levels 60 min after activation. Two interesting features emerged from a comparison of our data with those of Yang and colleagues [14]. First, de novo motif analysis did not reveal SP1 sites near RELA peaks of our most rapidly induced genes (Fig 2, pattern 3Ad). Thus, rapid gene induction by NF-κB comes in different flavors. One possibility is that SP1 and NF-κB cooperate at promoters where inducibility via NF-κB is coupled with relatively high basal-level expression via SP1. Second, in TNFα-treated A549 cells, RELA levels at a prototypical NF-κB/AP1 promoter continued to rise even when RNA levels were falling. By contrast, we found that at ERK-sensitive genes in activated BJAB cells, RELA levels fell, while RNA levels continued to rise. These distinctions yet again emphasize the variety of ways in which kinetic patterns of NF-κB-dependent transcription are achieved.

Third, half of the strongly induced direct target genes had preformed Pol II–containing loops in unactivated cells. Genes that contained such loops were induced more rapidly on average than genes without loops and reached close to maximal expression levels at 1 h post activation. In contrast, RNA levels of unlooped genes continued to increase in the interval between 1 and 4 h. We propose that kinetic patterns of NF-κB-dependent transcription are determined in part by a poised state reflected in such preformed loops. Interestingly, most of the transiently induced targets (22 out of 30) fell in the looped category, likely reflecting the need for these genes to reach maximum expression as soon as possible while RELA is still in the nucleus. These genes were also more evolutionarily conserved than prelooped genes that were induced more slowly (S6K Fig)[50]. However, several transiently induced genes (such as *TNFAIP3* and *NFKBIA*) did not have looped configurations in unactivated cells. One possibility is that these genes have “simple” NF-κB-dependent promoters that do not require interactions with distal regulatory sequences to modulate expression levels. Hao and Baltimore recently demonstrated that genes that are rapidly transcriptionally induced undergo rapid splicing to produce cytoplasmic mRNA [30]. Such mRNAs are also relatively unstable, resulting in transient gene induction. Five out of 7 genes that were shared between our dataset of transiently induced NF-κB target genes and that of Hao and Baltimore were found to have looped configurations in unactivated BJAB cells. Thus, a prelooped configuration may also assist in increasing splicing efficiency of rapidly induced genes. We note the caveat that the transformed state of BJAB cells may affect the distribution of genes with preformed loops.

We also identified many genes whose inducible expression increased in the presence of dnIκBα. A subset of these genes bound RELA in our ChIP-Seq analyses, suggesting that RELA binding attenuated transcription of these genes. Identification of AP1 motif as the prominent sequence at sites of RELA binding leads us to hypothesize that RELA is recruited by protein–protein interactions with transcription factors bound at these sites rather than by DNA binding. In doing so, RELA may reduce transcription activation function of the DNA-bound factor. Interestingly, genes encoding several AP1 motif-binding factors, such as *FOS*, *FOSB*, *MEF2B*, and *MEF2C*, were in this set of RELA-repressed genes, possibly indicating some form of regulatory feedback. Dual-specificity phosphatase 1 (DUSP1), a regulator of ERK activity, was also in this list, again suggesting cross talk between NF-κB and AP1 signaling cascades. At other genes, DNA-bound RELA might interfere with the progression of RNA polymerases, thereby reducing transcriptional output. Though the mechanism and functional importance of RELA-dependent down-regulation of gene expression remain largely speculative at this time, our studies highlight a mode of gene regulation by this transcription factor that has been largely overlooked.

## Methods

### Cell lines and materials

BJAB cells were cultured in RPMI 1640 medium supplemented with 10% FBS (HyClone), Penicillin-Streptomycin-Glutamine (Invitrogen), and 2-mercaptoethanol. For activation, cells were exposed to 50 ng/ml PMA and 2 μM ionomycin (Sigma-Aldrich) for 1 and 4 h. To inhibit ERK signaling, cells were pretreated with 0.33 nM PD0325901 (Selleckchem) for 1 h before P+I stimulation. To generate dnIκBα-inducible clones, BJAB cells were transfected with pcDNA6/TR (Invitrogen) to express Tet repressor (TetR), and stable clones were selected in 15 μg/ml blasticidin (Invitrogen) for 6 d. Stable single clones with the highest levels of TetR expression were subsequently transfected with full-length dnIκBα (S32A and S36A) cloned into pcDNA4/TO. Stable clones were selected in the presence of both blasticidin and 600 μg/ml zeocin (Invitrogen) for 6 d. dnIκBα expression was induced with 1 μg/ml Tet (Invitrogen) for 24 h before P+I treatment. All cell lines were maintained at 37°C with 5% CO2.

The following antibodies were used for ChIP, ChIP-Seq, or western blot: RelA (sc-372, Santa Cruz); Pol II (Covance Cat # MMS-126R); hnRNP (sc-32301, Santa Cruz); IκBα (sc-371, Santa Cruz); β-actin (sc-47778, Santa Cruz). Horseradish peroxidase–coupled goat anti-mouse IgG and goat anti-rabbit IgG (Santa Cruz) were used for immunoblotting.

### Immunoblotting

Proteins were separated by electrophoresis through 10% SDS-PAGE and electrophoretically transferred to nitrocellulose membrane (Millipore). After blocking with 5% dried milk in Tris-HCl-buffered saline/0.05% Tween (TBST) for 1 h, membranes were incubated with primary antibodies overnight, washed in TBST, and incubated for 1 h with horseradish peroxidase–coupled secondary antibodies (Santa Cruz). Proteins were detected by using the enhanced chemiluminescence (ECL) systems (Pierce) and Syngene Imaging System.

### ChIP and ChIP-Seq

ChIP experiments were performed as described by [51]. Briefly, cells were washed twice with PBS and then were fixed at room temperature with either 1% formaldehyde in PBS for 10 min (for Pol II ChIP) or 1.5 mM EGS (Pierce Cat # 21565) for 30 min followed by 1% formaldehyde (Sigma-Aldrich) at room temperature for 15 min in PBS (RELA ChIP). Reactions were quenched by adding glycine to a final concentration of 0.125 M, and cells were washed twice with cold PBS. Nuclei were isolated and lysed in buffer containing 50 mM Hepes-KOH, pH 7.5; 150 mM NaCl; 1 mM EDTA; 1% Triton X-100; 0.1% sodium deoxycholate; 0.1% SDS; and protease inhibitors. The crosslinked chromatin was subjected to fragmentation by sonication (Branson Sonicator). ChIP was performed with 2.5 μg anti-RelA antibody (Santa Cruz) and 2 μg anti-Pol II antibody (Covance) prebound to 50 μl Protein A or G Dynabeads (Invitrogen). Sonicated chromatin was added to antibody-bound beads and incubated at 4°C overnight. Beads were collected by centrifugation, washed, and incubated at 65°C for 4 h in elution buffer (50 mM Tris-HCl, pH 7.5; 10 mM EDTA; 1% SDS) to reverse cross-linking. ChIP DNA was purified by phenol-chloroform extraction followed by ethanol precipitation. For qPCR quantitation of ChIP, the signal from gene-specific amplicon was compared to an amplicon from the *H19* locus according to the equation RE = 2^- (C^_T_^(target gene)-C^_T_^(H19)^. Primer sequences are listed in Table 1.

**Table 1:** qRT-PCR primers sequence used in Fig 3A and S3E Fig.

Primer name	Sequence
*IL4I1* F	TTGAAAGGCACACGCTCT
*IL4I1* R	GCTGAGATAGAAGAAGCCATCC
*NFKB1* F	CACAAGGCAGCAAATAGACG
*NFKB1* R	GAGTTAGCAGTGAGGCACCA
*MAPK6* F	GACTGAGCCACACAAACCTT
*MAPK6* R	GGATGGGAGAGTGCTTCTTCT
*NFKBID* F	ACACGCTCCTTCACCTGTTT
*NFKBID* R	GGTCTTGCCCTTATGCTCAC
*TNFAIP3* F	TGTCCTCAGTTTCGGGAGAT
*TNFAIP3* R	GTCACCGTTCGTTTTCAGC
*RND1* F	GATAATGTCCGTCCACTCTGC
*RND1* R	GATTTCTGTCCTCCACTTCTTGA
*GAPDH* F	CACCCACTCCTCCACCTTT
*GAPDH* R	TTCCTCTTGTGCTCTTGCTG
*ZNF267* F	CTTCCGTATAATCGCCTGCT
*ZNF267* R	GGGAACACAGTGGTGGACTT
*HES1* F	AAGTGTGCTGGGGAAGTACC
*HES1* R	TTGATCTGGGTCATGCAGTT
*IRF1* F	ACCGAGCAATCCAAACACTT
*IRF1* R	AGCTCTACAACAGCCTGATTTC
*NFKBIA* F	GCAGGTTGTTCTGGAAGTTG
*NFKBIA* R	CTGGGGTTTTTCCCTCTCTT
*H19* F	CCCATCTTGCTGACCTCAC
*H19* R	AGACCTGGGACGTTTCTGTG

*NFKB1*, *IL4I1*, *MAPK6*, *RND1*, *TNFAIP3*, *NFKBID*, and *GAPDH* primers were used for testing the effect of the ERK inhibitor PD0325901 on NF-κB target genes’ expression, shown in Fig 3A. *ZNF267*, *HES1*, *IRF1*, *NFKBIA*, and *H19* primers were used for verifying the RELA recruitment to the target genes shown in S3E Fig.

Abbreviations: ERK, extracellular signal–regulated kinase; NF-κB, nuclear factor kappa B; qRT-PCR, quantitative real-time PCR.

For sequencing, adapters were ligated to the precipitated DNA fragments or the input DNA to construct a sequencing library according to the manufacturer’s protocol (Illumina, San Diego, CA, United States). Adapters with a T overhang were ligated to the DNA fragments and size selected (approximately 200–350 bases) on a 4.5% agarose gel. Eighteen cycles of PCR amplification were performed to enrich for fragments with an adaptor on both ends. These samples were bound to an Illumina single-read Flowcell, followed by cluster generation on the Illumina Cluster Station and sequencing with Illumina Genome Analyzer (GA-II). Two biological replicate ChIP-Seq experiments were carried out with each antibody. ChIP-Seq data are available on the GEO website (http://www.ncbi.nlm.nih.gov/geo/) (Accession number GSE117259).

### ChIP analysis

Bowtie2 software [52] was used to map quality-filtered reads from demultiplexed FASTQ files to human genome assembly GRCh37/hg19 with the default options. RELA ChIP-Seq peaks were called using standard parameters in MACS 2.1.0 [53] with input as the control and activated samples as the treatment. Peaks were called at an FDR ≤ 0.05. Peak annotation and motif finding were carried out with HOMER (http://homer.ucsd.edu/homer/). The HOMER program annotatePeaks.pl was used to annotate peaks with default parameters (promoter regions were defined from −1 kb to +100 bp). In our analysis, most intergenic peaks were located within 50 kb of TSSs. Two biological replicate experiments were carried out, and peaks with peak score ≥ 100 that were common to both replicates were used for all further analysis. All samples were normalized to 10 million reads for visualization. The programs findMotifsGenome.pl and findMotifs.pl were used to identify transcription factor binding motifs within peaks or promoter regions (−400 to +100 bp from TSSs). The program findGO.pl was used to assess the enrichment of various categories of gene function (GO).

### Total RNA purification and RT-PCR

Total RNA was extracted (2 × 10^6^ cells) using the Qiagen RNeasy Mini Kit (Qiagen). cDNA was synthesized with the SuperScript First Strand Synthesis System (Invitrogen Life Technologies). RT-PCR was performed in duplicates using the ABI PRISM 7000 (Applied Biosystems, Carlsbad, CA, US). Expression of *NFKB1*, *IL4I1*, *MAPK6*, *RND1*, *TNFAIP3*, and *NFKBID* was normalized to *GAPDH* mRNA on the same PCR plate. Relative expression (RE) of individual genes was calculated by the equation RE = 2^- (C^_T_^(target gene)-C^_T_^(GAPDH))^. Primer sequences are listed in Table 1.

### RNA-Seq sample preparation, sequencing, and data analysis

Total RNA purified from BJAB and transfected derivatives was used for bar-coded library preparation and sequencing at the Johns Hopkins Deep Sequencing & Microarray Core.

Two independent dnIκBα-inducible single-cell clones were treated with Tet for 24 h or not, prior to activation with P+I for different times. This experiment was performed twice for each clone, and RNA was prepared for sequencing.

RSEM [54] was used to align RNA-Seq reads to the human genome and to quantify transcript abundance. EBSeq [55] was used to compare the aligned reads from multiple conditions to find differentially expressed genes using a cutoff of FDR ≤ 0.05. *k*-means analysis of RNA expression data was carried out in MATLAB using normalized read counts, with correlation as the distance metric, the number of times to repeat clustering set to 5, and other parameters set to default. All samples were normalized to 1 million aligned reads for visualization. Integrative analysis of gene expression in relation to ChIP-Seq data was done by ngs.plot (https://github.com/shenlab-sinai/ngsplot). RNA-Seq data are available on the GEO website (http://www.ncbi.nlm.nih.gov/geo/) (Accession number GSE117259).

### RNA-Seq heatmap

Heatmaps of gene expression in S1C Fig (left), S1D Fig (left), S2C Fig (left), S3A Fig (left), and S4A Fig were generated using the package “gplots” in R program (https://CRAN.R-project.org/package=gplots) by log2-transformed normalized read counts after adding a pseudocount of 1. Colors represent standardized gene expression for which each gene is standardized across samples to have zero mean and unit standard deviation. The row color bar marks the cluster membership of each gene from the previous *k*-means clustering results. In addition, hierarchical clustering was applied based on the standardized gene expression. The results are shown in S1C (right) and S1D (right) Fig, S2C Fig (right), and S3A Fig (right) as heatmaps in which the rows are reordered by the hierarchical clustering results, and the row color bar represents the *k*-means clustering results.

### Silhouette calculation

The silhouette for each gene in which the expression is up-regulated ≥2 fold (1,021 genes in Fig 1B) in BJAB cells is based on correlation as the distance metric [56]. The silhouette value ranges from −1 to 1, where a larger value means that the gene is better matched to its own cluster than the neighboring clusters. As a baseline, we permuted cluster memberships and calculated the silhouette, which is shown in S1B Fig as the random group.

### ChIA-PET

RNA Pol II ChIA-PET was performed as previously described [41,42]. A total of 10^9^ BJAB cells were treated with 1.5 mM EGS (Pierce Cat # 21565) for 30 min, followed by 1% formaldehyde at room temperature for 15 min and then neutralized using 0.2 M glycine. Chromatin was sheared by sonication, and anti-Pol II monoclonal antibody 8WG16 (Covance, MMS-126R) was used to enrich Pol II–bound fragments. A portion of ChIP DNA was eluted from antibody-coated beads for quantitation using Picogreen fluorimetry and for enrichment analysis using qPCR. Two biological replicate ChIP samples were used for ChIA-PET library construction [42].

ChIA-PET data analysis was carried out as described by [43] (https://github.com/GuoliangLi-HZAU/ChIA-PET_Tool). Final peak calling was done at FDR ≤ 0.05. After filtering for paired-end tag (PET) clusters ≥ 2 counts and FDR ≤ 0.05, approximately 6,000 loops per replicate were obtained. The overlap between two replicates was 80%; data analysis was carried out from 1 replicate. ChIA-PET data are available on the GEO website (http://www.ncbi.nlm.nih.gov/geo/) (Accession number GSE117259).

All RNA-Seq, ChIP-Seq, and ChIA-PET data were visualized by preparing custom tracks for the WashU EpiGenome Browser [57,58]. All analyses were performed on Biowulf or Helix computer clusters at NIH.

### Evolutionary conservation analyses

Evolutionary conservation of NF-κB binding sites was calculated using a method similar to Iwanaszko and colleagues [50]. Homologous genes were obtained from NCBI HomoloGene (https://www.ncbi.nlm.nih.gov/homologene) for human, chimpanzee, rhesus, cattle, dog, mouse, and rat, followed by identification of promoter sequence (upstream 1,000 bp to TSS) of each gene for each species using R package biomaRt [59] based on the Ensembl database [60]. Promoter sequences of the other species (i.e., chimpanzee, rhesus, cattle, dog, mouse, and rat) were compared to the promoter sequence of human using R package msa [61]. We used R package TFBSTools [62] to identify conserved NF-κB binding sites between human and the other species. Four NF-κB family motifs (i.e., NFKB1: MA0105.2, NFKB2: MA0778.1, REL: MA0101.1, and RELA: MA0107.1) obtained from JASPAR [63] were used for the analysis. In accordance with Iwanaszko and colleagues [50], the threshold of the motif mapping score was set to 80%, and the conservation cutoff was set to 40%, based on a window size of 51 bp. To obtain the percentage of conserved NF-κB binding sites for each gene, the total number of NF-κB motif sites in the human genome was obtained based on the 4 NF-κB family motifs. The number of conserved NF-κB motif sites was calculated by comparing human and every other species, respectively. The percentage of conserved NF-κB binding sites is calculated as the ratio between the conserved NF-κB motif sites and the total number of NF-κB motif sites for each gene.

### Statistical analyses

In Fig 5B and 5C, data are represented as the mean with the SEM. Comparisons between two groups at each time point were assessed by a 1-way ANOVA Kruskal-Wallis test. A *p*-value of ≤0.05 was considered statistically significant.

## Supporting information

S1 Fig Kinetics of nuclear RELA induction and RNA-Seq analysis in activated BJAB cells and RELA ChIP-Seq summary. (A) Nuclear extracts from BJAB cells treated with P+I for the indicated times (h) were fractionated by SDS-PAGE and transferred to nitrocellulose membranes, and the membrane was probed with anti-RELA or anti-hnRNP (loading control) antibodies. Proteins were detected by HRP-coupled antibody. A representative western blot of 3 experiments is shown. (B) A total of 1,021 genes whose expression changed ≥2-fold in P+I-treated BJAB cells were categorized into 6 groups by *k*-means clustering (Fig 1B, upper). Silhouette (left) and elbow (right) analyses were used to corroborate *k*-means clustering. Silhouette calculation showed that 6 patterns show distinct distance compared to the random control. Underlying data for silhouette calculation are provided in S1E Data. Elbow analysis was consistent with the use of 5–7 clusters to visualize patterns of inducible gene expression. (C) Gene expression patterns determined by *k*-means clustering (Fig 1B, upper) were visualized in the form of a heatmap (left) after normalization of 2 replicate experiments by EBSeq. Numbers to the left of the heatmap identify the groups in Fig 1B (upper). The letter “A” after a pattern number indicates genes activated by P+I. Hierarchical clustering was applied to the same 1,021 up-regulated genes (right). Similar numbers of patterns were visualized by this method seen with *k*-means clustering (labeled A-F to the right of the heatmap). The color key represents normalized gene expression (see the Methods section). RNA-Seq data are available on the GEO website (http://www.ncbi.nlm.nih.gov/geo/) (Accession number GSE117259). (D) Heatmap representation of 900 genes whose expression was reduced ≥2-fold in response to activation were categorized by *k*-means clustering (left) or by hierarchical clustering (right) after normalization of 2 replicate experiments by EBSeq. Numbers to the left of the *k*-means heatmap correspond to patterns depicted in Fig 1B (lower). The letter “R” after a pattern number indicates genes repressed by P+I. A–F to the right of the hierarchical clustering heatmap correspond to possible grouping of coregulated genes by this method. (E) GO (findGO.pl from HOMER) analysis of coregulated genes that fell in patterns (1–6) identified by *k*-means clustering of 1,021 up-regulated genes in activated BJAB cells (Fig 1B, upper). The letter “A” after a pattern number indicates genes activated by P+I. The top 10 biological processes for each pattern are shown. (F) Scatterplots depicting correlation between 2 biological replicate RELA ChIP-Seqs for the indicated times. Peaks were called for each replicate using MACS2. After peak annotation by HOMER, RELA peaks with peak score ≥ 100 that were common to both replicates were identified and used in further analysis. RELA ChIP-Seq data are available on the GEO website (http://www.ncbi.nlm.nih.gov/geo/) (Accession number GSE117259). (G) Motif analysis of sequences underlying such shared peaks was carried out using HOMER (http://homer.ucsd.edu/homer/). Numbers of RELA peaks at each time point are indicated in parentheses, and the top 4 motifs for each time point are shown. ChIP-Seq, chromatin immunoprecipitation and sequencing; GEO, Gene Expression Omnibus; GO, Gene Ontology; hnRNP, heterogeneous ribonucleoprotein particle; HRP, horseradish peroxidase; P+I, phorbol 12-myristate 13-acetate and ionomycin; RNA-Seq, RNA sequencing.
(PDF)

S2 Fig Gene expression in dnIκBα-expressing BJAB clones and identification of RELA target genes. Two clones of BJAB cells were generated in which dnIκBα could be induced by Tet treatment. (A) Basal gene expression (in the absence of P+I treatment) in each clone, in the absence or presence of Tet (-Tet or +Tet), was compared to basal gene expression in untransfected BJAB cells using EBSeq with an FDR threshold ≤ 0.05. Numbers of genes in each pattern are shown in the table. The largest numbers of genes were expressed comparably in all these cell lines (pattern 1) or unchanged in the presence or absence of Tet in each clone (pattern 4). We identified approximately 2,700 genes that were changed at baseline in both dnIκBα-inducible clones compared to parental BJAB cells (overlap between pattern 4 genes in clone 1 and 2 with FDR ≤ 0.05 compared to parental BJAB cells). (B) Differentially expressed genes that were common to both clones were further compared to parental BJAB cells after filtering (FDR ≤ 0.05) by fold change. Graphs show down- or up-regulated genes, respectively, sorted by fold change. The majority of differentially expressed genes were changed less than 2-fold. Underlying data for this figure are provided in S1F Data. (C) Heatmap representation of 304 direct RELA target genes identified by combining results of RNA-seq analysis in the presence or absence of dnIκBα with RELA ChIP-Seq (Fig 2C, right). Gene expression patterns were identified using *k*-means clustering (left and Fig 2C) or hierarchical clustering (right) after normalization of 2 replicate experiments by EBSeq. Each column shows the level of expression in the absence of dnIκBα (3 lanes labeled -Tet) or the presence of dnIκBα (3 lanes labeled +Tet). The activation time course is indicated. Numbers to the left of the *k*-means heatmap correspond to patterns shown in Fig 2C (right). The letters “Ad” after a pattern number indicate direct target genes that were activated by RELA binding. Each column is the averaged expression from 2 biological replicates. (D) Motif analysis of the promoter region (−400 to +100 bp) of the 304 direct RELA target genes (left) or under the RELA peaks (right) was carried out using HOMER. (E) *k*-means clustering was used to categorize the 304 direct RELA target genes into 6 patterns of inducible expression (Fig 2C, right). The most prominent patterns consisted of genes that were continuously up-regulated with activation (patterns 2Ad and 4Ad) or those that were transiently up-regulated with activation (pattern 3Ad). De novo motif analysis of sequences with RELA peaks was carried out using HOMER for each of these patterns as indicated. Pattern 3Ad revealed only one motif that was identified as not “possibly false positive.” The letters “Ad” after a pattern number indicate direct target genes that were activated by RELA binding. (F) GO analysis of coregulated RELA target genes (304) in each pattern identified by *k*-means clustering (Fig 2C, right). The letters “Ad” after a pattern number indicate direct target genes that were activated by RELA binding. The top 10 biological processes for each pattern are shown. (G) RNA tracks showing complete time courses for RELA target genes shown in Fig 2D. ChIP-Seq, chromatin immunoprecipitation and sequencing; dnIκBα, dominant negative NFKB inhibitor alpha; GO, Gene Ontology; P+I, phorbol 12-myristate 13-acetate and ionomycin; RNA-Seq, RNA sequencing; Tet, tetracycline.
(PDF)

S3 Fig Promoter motif analysis of indirect RELA target genes. Genes whose induction was reduced by dnIκBα expression but which did not bind RELA were considered to be indirect targets of RELA (see text). (A) Heatmap representation of 502 indirect RELA target genes identified by combining results of RNA-seq analysis in the presence or absence of dnIκBα with RELA ChIP-Seq (Fig 2C, left). Gene expression patterns were identified using *k*-means clustering (left and Fig 2C) or hierarchical clustering (right). Each column shows the level of expression in the absence of dnIκBα (3 lanes labeled -Tet) or the presence of dnIκBα (3 lanes labeled +Tet). Activation time course is indicated. Numbers to the left of the *k*-means heatmap correspond to patterns shown in Fig 2C (left). The letters “Ai” after a pattern number indicate target genes that were indirectly activated by RELA. Each column is the averaged expression from 2 biological replicate experiments. (B) Motif analysis of the promoter regions (−400 to +100 bp) of 502 indirect target genes was carried out using HOMER. The consensus sequence for MYC was the most enriched motif. (C) Browser tracks showing that the *MYC* gene is a RELA target in BJAB cells. (D) Genes encoding transcription factors were identified from RELA-activated (left) or RELA-repressed target genes (Figs 2 and 4). Databases used to identify genes that encode transcription factors were from a vertebrate transcription factor database (http://jaspar.genereg.net/downloads/), a human transcription factor list (http://www.tfcheckpoint.org/index.php/browse), and 2 GO groups (GO:0043565—sequence-specific DNA binding and GO:0003700—transcription factor activity, sequence-specific DNA binding). (E) RELA recruitment to identified target genes (*ZNF267*, *HES1*, and *IRF1*) was evaluated in activated BJAB cells by anti-RELA ChIP followed by qPCR (top 2 rows). *NFKBIA* gene was used as the positive control and H19 as the negative control. Results shown are the average of 2 independent ChIP experiments. Cells pretreated with Tet (+Tet) or not were activated for 1 h with P+I followed by ChIP and qPCR analysis. dnIκBα expression (+Tet) reduced RELA recruitment to all tested genes (blue bars). RelA ChIP was carried out in 1 dnIκBα-inducible clone. Error bars represent the standard error of the mean between experiments. Underlying data for this figure are provided in S1G Data. (F) Promoter regions of the top 78 indirect targets that were changed ≥2-fold in the absence of Tet and whose expression was reduced by dnIκBα in both clones (FDR ≤ 0.05) were analyzed using HOMER to identify putative transcription factor binding sites. The table shows transcription factor motifs that are present in the promoters of at least 15 (20%) of the 78 genes whose RNA levels were decreased by dnIκBα. (G) GO analysis of coregulated indirect RELA target genes in each pattern (1–6Ai) identified by *k*-means clustering (Fig 2C, left). The letters “Ai” after a pattern number indicate target genes that were indirectly activated by RELA. The top 10 biological processes for each pattern are shown. (H) RNA tracks showing complete time courses for direct RELA target genes shown in Fig 3B. ChIP-Seq, chromatin immunoprecipitation and sequencing; dnIκBα, dominant negative NFKB inhibitor alpha; FDR, false discovery rate; GO, Gene Ontology; P+I, phorbol 12-myristate 13-acetate and ionomycin; qPCR, quantitative PCR; RNA-Seq, RNA sequencing; Tet, tetracycline.
(PDF)

S4 Fig Characterization of RELA binding and motif analysis of genes whose RNA levels were increased by dnIκBα expression. Such genes were considered to be repressed by inducible NF-κB in BJAB cells. Analogous to the discussion of inducibly activated genes, repressed genes that bound RELA were likely to be affected directly by RELA, whereas those that did not were considered to be indirectly affected (see text). (A) Heatmap representation of 85 direct RELA-repressed target genes (left) and 178 indirect RELA-repressed target genes (right) identified by combining results of RNA-seq analysis in the presence or absence of dnIκBα with RELA ChIP-Seq (Fig 4A). Gene expression patterns were identified using *k*-means clustering. Each column shows the level of expression in the absence of dnIκBα (3 lanes labeled -Tet) or the presence of dnIκBα (3 lanes labeled +Tet). The activation time course is indicated. Numbers to the left of the *k*-means heatmap correspond to patterns shown in Fig 4A. The letters “Rd” after a pattern number indicate target genes that were repressed by RELA binding, and the letters “Ri” after a pattern number indicate target genes that were repressed by RELA indirectly. Each column is the averaged expression from 2 biological replicate experiments. (B) RNA tracks showing complete time courses for RELA-repressed target genes shown in Fig 4B. (C) Motif analysis of DNA sequences under RELA peaks and promoter regions (−400 to +100 bp) of genes whose expression was increased by dnIκBα (FDR ≤ 0.05). AP1 and NF-κB motifs were prominent under RELA peaks; no significantly enriched motifs were identified in the promoters of these genes. (D) ngs.plot demonstrating the distribution of RELA binding near up-regulated target genes (130, from Fig 2B) and down-regulated target genes (53, from Fig 4A) whose inducible expression was changed ≥2-fold in the absence of Tet. Most RELA peaks were located at promoter regions of up-regulated target genes, while RELA peaks were distributed throughout the gene of down-regulated target genes. (E) Amongst genes whose inducible expression was increased by dnIκBα expression but did not bind RELA (genes indirectly down-regulated by NF-κB activation), 58 genes were identified whose inducible activity changed ≥2-fold (FDR ≤ 0.05). Promoter regions (−400 to +100 bp) of these genes were scanned for transcription factor binding motifs using HOMER. The table shows motifs that were present in more than 12 (out of 58) promoters. (F) Comparison of promoter motifs in genes that were indirectly up- (78) or down- (57) regulated by RELA. A total of 77 transcription factor motifs were found in ≥20% of indirectly up-regulated genes (blue circle), and 95 motifs were found in ≥20% of indirectly down-regulated genes (pink circle). The majority of the motifs were common to both gene subsets, presumably reflecting context-specific use of the same factors to up- or down-regulate gene expression (left). Motifs unique to genes that are (indirectly) activated by RELA are shown in the middle table, and those unique to genes that are (indirectly) repressed by RELA are shown in the table on the right. Four out of the 5 unique motifs associated with up-regulated genes correspond to the recognition site of bHLH proteins (CANNTG). Several different transcription factor motifs are associated with down-regulated genes (right table). (G) RNA tracks of *SOX8* and *PPARG* showing these 2 genes are up-regulated by dnIκBα. (H) GO analysis of coregulated RELA-repressed direct target genes in each pattern (1–6Rd) identified by *k*-means clustering (Fig 4A, right). The letters “Rd” after a pattern number indicate target genes that were repressed by RELA binding. The top 10 biological processes for each pattern are shown. (I) GO analysis of coregulated RELA target repressed indirect genes in each pattern (1–6Ri) identified by *k*-means clustering (Fig 4A, left). The letters “Ri” after a pattern number indicate target genes that were indirectly repressed by RELA. The top 10 biological processes for each pattern are shown. AP1, activator protein 1; bHLH, basic helix-loop-helix; ChIP-Seq, chromatin immunoprecipitation and sequencing; dnIκBα, dominant negative NFKB inhibitor alpha; FDR, false discovery rate; GO, Gene Ontology; NF-κB, nuclear factor kappa B; PPARG, peroxisome proliferator–activated receptor gamma; RNA-Seq, RNA sequencing; SOX8, SRY-box 8; Tet, tetracycline.
(PDF)

S5 Fig Alternative analyses of the NF-κB transcriptome. (A) RNA-Seq data from 2 biological replicates in each Tet-inducible clone were first used to define a set of genes whose expression did not change in response to cell activation and dnIκBα expression in both clones. We identified approximately 8,000 genes whose expression did not change with P+I treatment and dnIκBα expression. Of these, approximately 600 showed inducible RELA binding (green circle). (B) HOMER analysis of sequences underlying RELA peaks of these 600 genes identified authentic κB and AP1 motifs. No motifs were enriched at the promoters of these genes (−400 to +100 bp). (C) Examples of inducible RELA binding to genes that are unaffected by P+I treatment and dnIκBα expression. The top 6 lines show RNA-Seq tracks at different time points of P+I treatment and in the presence (+Tet) or absence (-Tet) of dnIκBα. The bottom 4 lines (including input track) show RELA ChIP-Seq tracks at different activation time points. (D) The remaining genes, whose expression changed in response to activation in both clones, were visualized by *k*-means clustering with correlation parameter in the absence (blue lines) or presence (red lines) of dnIκBα. Patterns were combined based on similar trends to generate 4 groups, which are indicated by colored boxes denoting the following characteristics: I (red boxes) = genes down-regulated by dnIκBα; II (purple boxes) = genes up-regulated by dnIκBα; III (yellow boxes) = genes up-regulated by P+I but unaffected by dnIκBα; and IV (blue boxes) = genes down-regulated by P+I but unaffected by dnIκBα. (E) RELA binding and promoter characteristics of genes in each group. Green circles denote RELA-binding genes identified by ChIP-Seq; gene numbers in each category are indicated. HOMER analysis was used to identify sequence motifs underlying RELA peaks (middle panel) or in the promoters (−400 to +100 bp relative to TSSs) of genes that did not bind RELA (right panel). (F) Representative examples of RNA expression and RELA binding to genes from groups III and IV. Numbers on the top line show genomic coordinates in hg19; genomic organization and transcription orientation are noted below the tracks. The *y* axes correspond to normalized reads per million for RNA and normalized reads per 10 million for ChIP-Seq. AP1, activator protein 1; ChIP-Seq, chromatin immunoprecipitation and sequencing; dnIκBα, dominant negative NFKB inhibitor alpha; NF-κB, nuclear factor kappa B; P+I, phorbol 12-myristate 13-acetate and ionomycin; RNA-Seq, RNA sequencing; Tet, tetracycline; TSS, transcription start site.
(PDF)

S6 Fig Analysis of Pol II ChIP-Seq and Pol II ChIA-PET. (A) Scatterplots depicting correlation between 2 replicates of Pol II ChIP-Seq for the indicated times. Further analyses were restricted to Pol II peaks with peak score ≥ 100 that were present in both biological replicates. Pol II ChIP-Seq data are available on the GEO website (http://www.ncbi.nlm.nih.gov/geo/) (Accession number GSE117259). (B) Pol II binding (0 h) to direct and indirect RELA target genes that are induced ≥2-fold by P+I in activated cells. The total number of genes in each category is noted in parentheses. (C) Pol II loading at 130 direct (induced ≥2-fold) RELA target genes as identified in Fig 2 shows recruited Pol II binding (left) after normalizing to gene length between annotated TSSs and TTSs. Tracks corresponding to different activation times are color-coded as indicated. Fifty out of 130 genes that have the pre-Pol II binding (S6B Fig) also show recruited Pol II binding (right). (D) Browser tracks of genes showing inducible Pol II recruitment in response to cell activation. The top 2 tracks show RNA-Seq tracks in the presence or absence of tetracycline-induced dnIκBα at 1 h. The center track shows the RELA ChIP-Seq track in BJAB cells at 1 h. The bottom tracks show Pol II ChIP-Seq in BJAB cells activated for the indicated times. (E) Pol II loading at 78 “indirect” (induced ≥2-fold) target genes as identified in Fig 2 is shown after normalizing to gene length between annotated TSSs and TTSs. Tracks corresponding to different activation times are color-coded as indicated. (F) RNA expression at baseline (in the absence of P+I) for genes in different ChIA-PET categories from Fig 5A. Genes with single-gene-based (Category III) or multiple-gene-based (Category IV) loops have higher RNA levels compared to genes that bind Pol II but do not display looping interactions (Category II). Underlying data for this figure are provided in S1H Data. Only the genes expressed in BJAB cells were used for statistical calculation. Statistical significance was tested by 1-way ANOVA analysis (*p* < 0.001). ChIA-PET data are available on the GEO website (http://www.ncbi.nlm.nih.gov/geo/) (Accession number GSE117259). (G) Comparison of looping interactions in BJAB cells (this study) and previous published studies in K562 cells (http://vizhub.wustl.edu/hubSample/hg19/K562POL2.gz). (H) GO analysis of genes in ChIA-PET Category IV (multilooped configuration) in BJAB cells. Top GO classifications included genes involved in essential metabolic pathways. (I) Representative examples of multilooped interactions in BJAB cells. (J) RNA tracks showing complete time courses for RELA direct target genes with prebound Pol II loop (left) and indirect target with prebound Pol II loop (right) shown in Fig 5D. (K) Conservation analyses showed early-response genes with prebound loops from pattern 3Ad in Fig 5B (22) have higher conservation evolution than late-response genes (40) with prebound loops from patterns 2Ad and 4Ad in Fig 5B. Underlying data for this figure are provided in S1I Data. ChIA-PET, chromatin interaction analysis by paired-end tag sequencing; ChIP-Seq, chromatin immunoprecipitation and sequencing; dnIκBα, dominant negative NFKB inhibitor alpha; GEO, Gene Expression Omnibus; GO, Gene Ontology; P+I, phorbol 12-myristate 13-acetate and ionomycin; Pol II, RNA polymerase II; RNA-Seq, RNA sequencing; TSS, transcription start site; TTS, transcription termination site.
(PDF)

S1 Table Putative RELA target genes based on inducible transcription and RELA binding. Transcriptional response of BJAB cells to P+I was assessed by RNA-Seq after 1 and 4 h of activation. Differentially expressed genes were identified by EBSeq [55] (Fig 1A). RELA binding over the same time course was assessed by ChIP-Seq. RELA-bound regions with peak score ≥ 100 that were reproduced in biological replicates were used for all analyses. RELA binding was ascribed to a specific gene according to HOMER using the default parameters (http://homer.ucsd.edu/homer/). For most of these genes, RELA bound at the promoter, within the gene body, or within 50 kb of the transcription start site. RELA-binding genes that were up-regulated ≥2-fold at either 1 or 4 h are shown in A. RELA-binding genes that were down-regulated ≥2-fold at either 1 or 4 h are shown in B. ChIP-Seq, chromatin immunoprecipitation and sequencing; P+I, phorbol 12-myristate 13-acetate and ionomycin; RNA-Seq, RNA sequencing.
(PDF)

S2 Table RELA-activated direct target genes. A total of 304 direct RELA target genes that were identified in this study based on inducible activation in response to P+I, RELA binding by ChIP-Seq, and down-regulation by dnIκBα. Genes marked in red were changed more than 2-fold in the absence of tetracycline. Genes marked in green are newly identified target genes not previously noted in a cumulative list culled from multiple databases (http://www.bu.edu/nf-kb/gene-resources/target-genes/) (https://www.yumpu.com/en/document/view/8327926/the-nfkb-target-gene-sets-are-listed-below-broad-institute) [24,27]. ChIP-Seq, chromatin immunoprecipitation and sequencing; dnIκBα, dominant negative NFKB inhibitor alpha; P+I, phorbol 12-myristate 13-acetate and ionomycin.
(PDF)

S3 Table Genes whose expression is targeted indirectly by RELA via RELA-induced transcription factors. List of genes whose expression was reduced by dnIκBα but which did not bind RELA. We refer to these genes as “indirect” RELA targets (see text). Genes in the list were changed in expression ≥2-fold in response to P+I treatment in the absence of tetracycline. Genes marked in red contain the bHLH protein binding motif (CANNTG) in their promoters (−400 to +100 bp). This motif—which is the recognition site of MYC, MITF, AHR, and NPAS2—is enriched in indirectly up-regulated genes (see text and S4 Fig). AHR, aryl hydrocarbon receptor; bHLH, basic helix-loop-helix; dnIκBα, dominant negative NFKB inhibitor alpha; MITF, microphthalmia-associated transcription factor; NPAS2, neuronal PAS domain protein 2; P+I, phorbol 12-myristate 13-acetate and ionomycin.
(PDF)

S4 Table RELA-suppressed target genes. Eighty-five RELA-binding genes whose inducible expression was up-regulated by dnIκBα expression. Genes marked in red were affected ≥2-fold in response to P+I treatment in the absence of tetracycline. Genes marked in green have not been previously noted in databases of NF-κB target genes. dnIκBα, dominant negative NFKB inhibitor alpha; NF-κB, nuclear factor kappa B; P+I, phorbol 12-myristate 13-acetate and ionomycin.
(PDF)

S5 Table Genes whose expression is repressed indirectly by RELA via RELA-induced transcription factors. List of genes whose expression was increased by dnIκBα but which did not bind RELA. We refer to these genes as “indirect” RELA targets (see text). Genes in the lists were changed in expression ≥2-fold in response to P+I treatment in the absence of tetracycline. dnIκBα, dominant negative NFKB inhibitor alpha; P+I, phorbol 12-myristate 13-acetate and ionomycin.
(PDF)

S6 Table Looping status of NF-κB target genes in unactivated cells. (A) Fifty out of 130 direct NF-κB target genes have Pol II binding in unactivated cells. (B) Pol II–associated loops in unactivated cells of direct RELA target genes (130 genes, column 1 and 2) and indirect RELA target genes (78 genes, column 3 and 4) that are induced more than 2-fold in response to cell activation. Genes with preformed Pol II–associated loops are in the first column of each set. NF-κB, nuclear factor kappa B; Pol II, RNA polymerase II.
(PDF)

S1 Data Data underlying Figs: 3A, 3C, 5B, 5C, S1B, S2B, S3E, S6F and S6K. (XLSX)

## Abbreviations

AP1: activator protein 1
BAFF: B cell activating factor
BAFF-R: BAFF receptor
BCR: B cell receptor
ChIA-PET: chromatin interaction analysis by paired-end tag sequencing
ChIP: chromatin immunoprecipitation
ChIP-Seq: chromatin immunoprecipitation and sequencing
dnIκBα: dominant negative IκBα
DUSP1: dual-specificity phosphatase 1
ECL: enhanced chemiluminescence
ERK: extracellular signal–regulated kinase
FDR: false discovery rate
FoxM1: forkhead box M1
GO: Gene Ontology
H3S10P: serine phosphorylated histone H3
HES1: hes family bHLH transcription factor 1
IκB: NFKB inhibitor
IKK: inhibitor of NF-κB kinase
IL-1β: interleukin 1 beta
IRF: interferon regulatory factor
KLF: kruppel-like factor
LPS: lipopolysaccharide
MAPK: mitogen-activated protein kinase
NFAT: nuclear factor of activated T cell
NF-κB: nuclear factor kappa B
NFKB1: nuclear factor kappa B subunit 1
NFKB2: nuclear factor kappa B subunit 2
P+I: PMA and ionomycin
PET: paired-end tag
PMA: phorbol 12-myristate 13-acetate
Pol II: RNA polymerase II
qPCR: quantitative PCR
qRT-PCR: quantitative real-time PCR
RE: relative expression
RNA-Seq: RNA sequencing
SOX: SRY-box
STAT: signal transducer and activator of transcription
TBST: Tris-HCl-buffered saline/0.05% Tween
Tet: tetracycline
TetR: Tet repressor
TLR4: Toll-like receptor 4
TNFα: tumor necrosis factor alpha
TSS: transcription start site
TTS: transcription termination site
UTR: untranslated region
YY1: Yin Yang 1
ZNF: zinc finger protein
