# Supplementary material for: Transcriptional outcomes and kinetic patterning of gene expression in response to NF-κB activation
Source: PLoS Biol. 2018 Sep 10;16(9):e2006347. doi: 10.1371/journal.pbio.2006347 (PMC6147668; doi:10.1371/journal.pbio.2006347)
Supplement: S1 Fig — (A) Nuclear extracts from BJAB cells treated with P+I for the indicated times (h) were fractionated by SDS-PAGE and transferred to nitrocellulose membranes, and the membrane was probed with anti-RELA or anti-hnRNP (loading control) antibodies. Proteins were detected by HRP-coupled antibody. A representative western blot of 3 experiments is shown. (B) A total of 1,021 genes whose expression changed ≥2-fold in P+I-treated BJAB cells were categorized into 6 groups by k-means clustering (Fig 1B, upper). Silhouette (left) and elbow (right) analyses were used to corroborate k-means clustering. Silhouette calculation showed that 6 patterns show distinct distance compared to the random control. Underlying data for silhouette calculation are provided in S1E Data. Elbow analysis was consistent with the use of 5–7 clusters to visualize patterns of inducible gene expression. (C) Gene expression patterns determined by k-means clustering (Fig 1B, upper) were visualized in the form of a heatmap (left) after normalization of 2 replicate experiments by EBSeq. Numbers to the left of the heatmap identify the groups in Fig 1B (upper). The letter “A” after a pattern number indicates genes activated by P+I. Hierarchical clustering was applied to the same 1,021 up-regulated genes (right). Similar numbers of patterns were visualized by this method seen with k-means clustering (labeled A-F to the right of the heatmap). The color key represents normalized gene expression (see the Methods section). RNA-Seq data are available on the GEO website (http://www.ncbi.nlm.nih.gov/geo/) (Accession number GSE117259). (D) Heatmap representation of 900 genes whose expression was reduced ≥2-fold in response to activation were categorized by k-means clustering (left) or by hierarchical clustering (right) after normalization of 2 replicate experiments by EBSeq. Numbers to the left of the k-means heatmap correspond to patterns depicted in Fig 1B (lower). The letter “R” after a pattern number indicates genes [file pbio.2006347.s001.pdf]

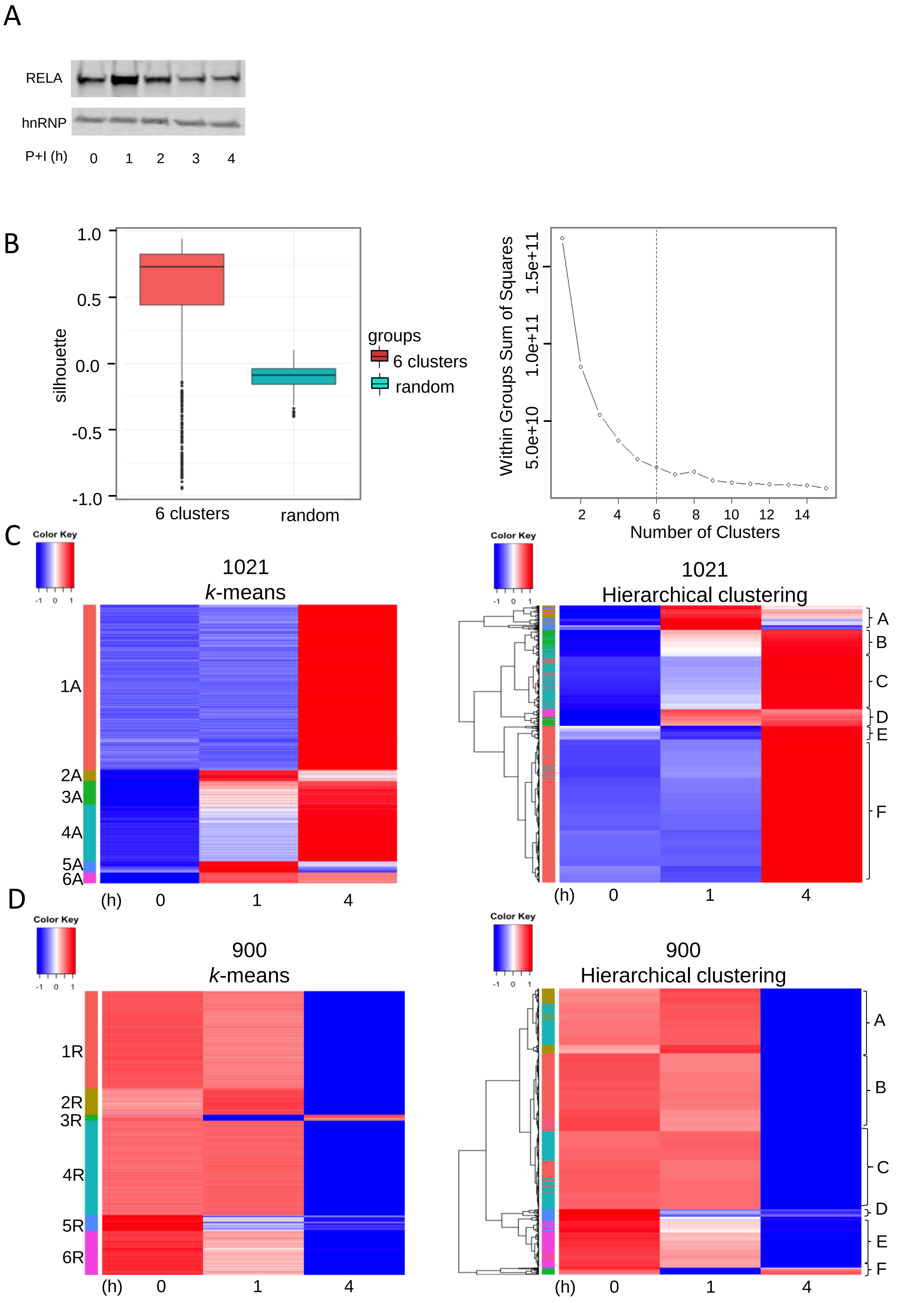
A
RELA
hnRNP
P+I (h) 0 1 2 3 4
B
silhouette
1.0
0.5
0.0
-0.5
-1.0
6 clusters
random
groups
6 clusters
random
Within Groups Sum of Squares
1.5e+11
1.0e+11
5.0e+10
2 4 6 8 10 12 14
Number of Clusters
C
Color Key
-1 0 1
1021
k-means
1A
2A
3A
4A
5A
6A
(h) 0 1 4
1021
Hierarchical clustering
A
B
C
D
E
F
(h) 0 1 4
D
900
k-means
1R
2R
3R
4R
5R
6R
(h) 0 1 4
900
Hierarchical clustering
A
B
C
D
E
F
(h) 0 1 4

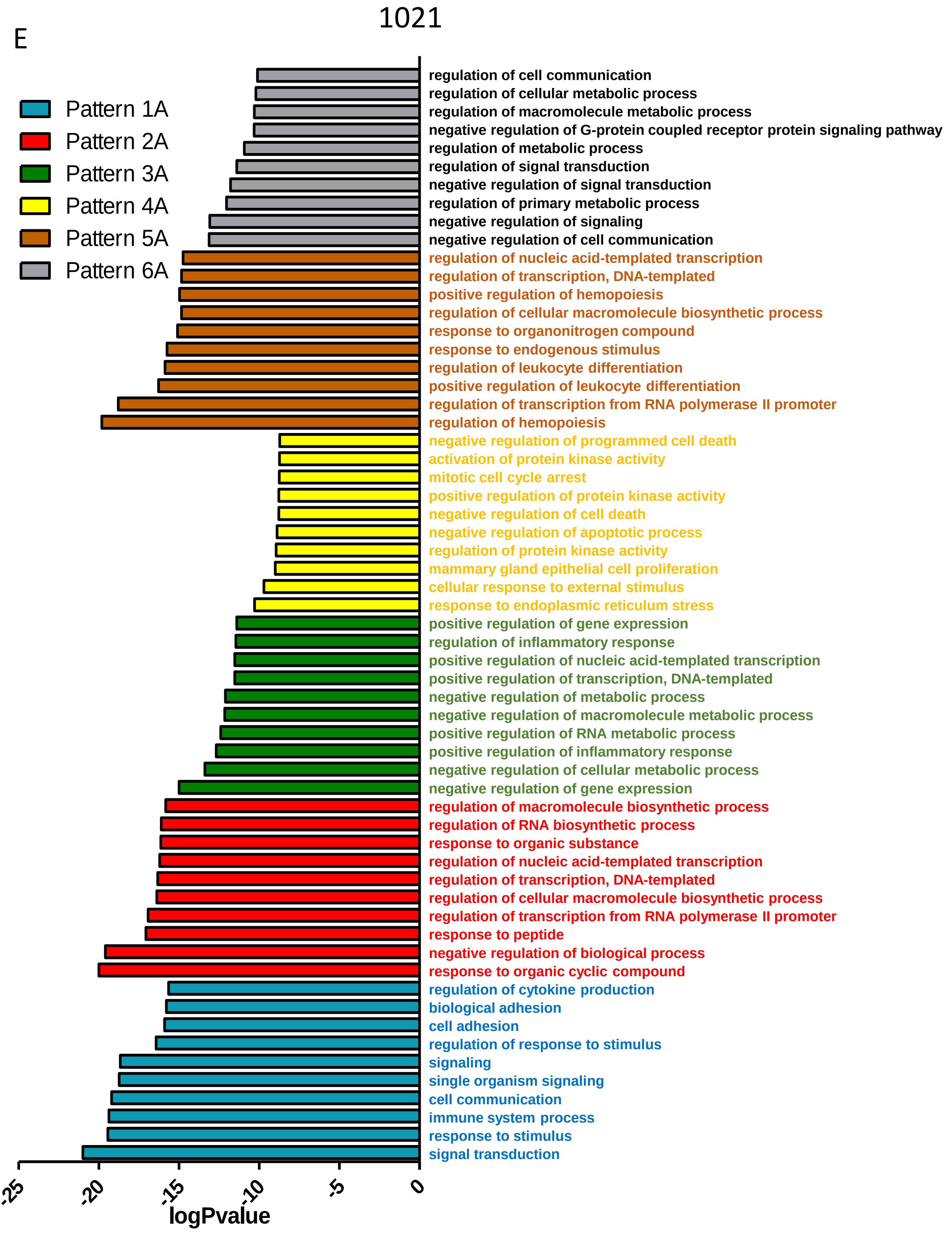
E
1021
Pattern 1A
Pattern 2A
Pattern 3A
Pattern 4A
Pattern 5A
Pattern 6A
regulation of cell communication
regulation of cellular metabolic process
regulation of macromolecule metabolic process
negative regulation of G-protein coupled receptor protein signaling pathway
regulation of metabolic process
regulation of signal transduction
negative regulation of signal transduction
regulation of primary metabolic process
negative regulation of signaling
negative regulation of cell communication
regulation of nucleic acid-templated transcription
regulation of transcription, DNA-templated
positive regulation of hemopoiesis
regulation of cellular macromolecule biosynthetic process
response to organonitrogen compound
response to endogenous stimulus
regulation of leukocyte differentiation
positive regulation of leukocyte differentiation
regulation of transcription from RNA polymerase II promoter
regulation of hemopoiesis
negative regulation of programmed cell death
activation of protein kinase activity
mitotic cell cycle arrest
positive regulation of protein kinase activity
negative regulation of cell death
negative regulation of apoptotic process
regulation of protein kinase activity
mammary gland epithelial cell proliferation
cellular response to external stimulus
response to endoplasmic reticulum stress
positive regulation of gene expression
regulation of inflammatory response
positive regulation of nucleic acid-templated transcription
positive regulation of transcription, DNA-templated
negative regulation of metabolic process
negative regulation of macromolecule metabolic process
positive regulation of RNA metabolic process
positive regulation of inflammatory response
negative regulation of cellular metabolic process
negative regulation of gene expression
regulation of macromolecule biosynthetic process
regulation of RNA biosynthetic process
response to organic substance
regulation of nucleic acid-templated transcription
regulation of transcription, DNA-templated
regulation of cellular macromolecule biosynthetic process
regulation of transcription from RNA polymerase II promoter
response to peptide
negative regulation of biological process
response to organic cyclic compound
regulation of cytokine production
biological adhesion
cell adhesion
regulation of response to stimulus
signaling
single organism signaling
cell communication
immune system process
response to stimulus
signal transduction
-25
-20
-15
-10
-5
0
logPvalue

F

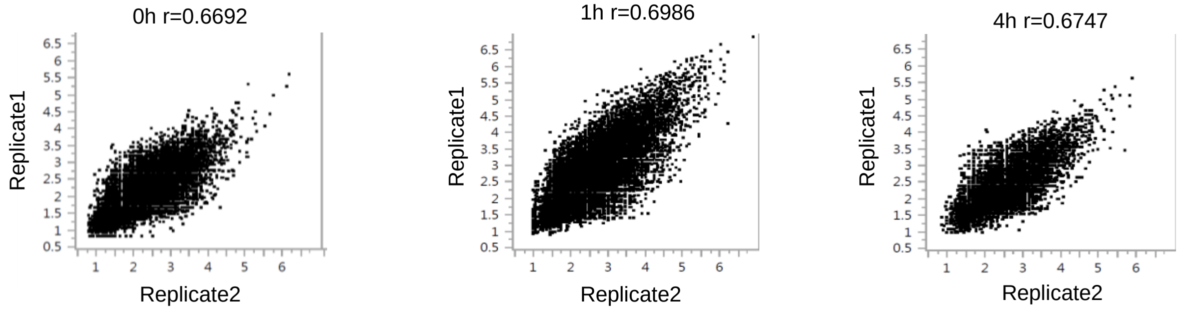

G

0h (345)

Consensus sequence	P-value	Motif Name
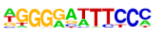	1e-24	NFkB-p65
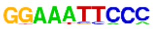	1e-22	NFkB-p65-Rel
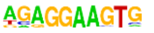	1e-16	PU.1(ETS)
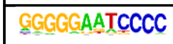	1e-11	NFkB-p50,p52

1h (2847)

Consensus sequence	P-value	Motif Name
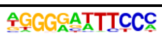	1e-682	NFkB-p65
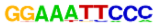	1e-384	NFkB-p65-Rel
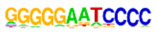	1e-343	NFkB-p50,p52
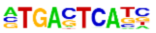	1e-317	AP.1

4h (593)

Consensus sequence	P-value	Motif Name
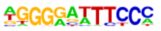	1e-147	NFkB-p65
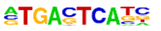	1e-142	AP.1
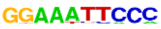	1e-100	NFkB-p65-Rel
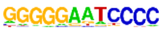	1e-90	NFkB-p50,p52
