# Supplementary material for: Transcriptional outcomes and kinetic patterning of gene expression in response to NF-κB activation
Source: PLoS Biol. 2018 Sep 10;16(9):e2006347. doi: 10.1371/journal.pbio.2006347 (PMC6147668; doi:10.1371/journal.pbio.2006347)
Supplement: S2 Fig — Two clones of BJAB cells were generated in which dnIκBα could be induced by Tet treatment. (A) Basal gene expression (in the absence of P+I treatment) in each clone, in the absence or presence of Tet (-Tet or +Tet), was compared to basal gene expression in untransfected BJAB cells using EBSeq with an FDR threshold ≤ 0.05. Numbers of genes in each pattern are shown in the table. The largest numbers of genes were expressed comparably in all these cell lines (pattern 1) or unchanged in the presence or absence of Tet in each clone (pattern 4). We identified approximately 2,700 genes that were changed at baseline in both dnIκBα-inducible clones compared to parental BJAB cells (overlap between pattern 4 genes in clone 1 and 2 with FDR ≤ 0.05 compared to parental BJAB cells). (B) Differentially expressed genes that were common to both clones were further compared to parental BJAB cells after filtering (FDR ≤ 0.05) by fold change. Graphs show down- or up-regulated genes, respectively, sorted by fold change. The majority of differentially expressed genes were changed less than 2-fold. Underlying data for this figure are provided in S1F Data. (C) Heatmap representation of 304 direct RELA target genes identified by combining results of RNA-seq analysis in the presence or absence of dnIκBα with RELA ChIP-Seq (Fig 2C, right). Gene expression patterns were identified using k-means clustering (left and Fig 2C) or hierarchical clustering (right) after normalization of 2 replicate experiments by EBSeq. Each column shows the level of expression in the absence of dnIκBα (3 lanes labeled -Tet) or the presence of dnIκBα (3 lanes labeled +Tet). The activation time course is indicated. Numbers to the left of the k-means heatmap correspond to patterns shown in Fig 2C (right). The letters “Ad” after a pattern number indicate direct target genes that were activated by RELA binding. Each column is the averaged expression from 2 biological replicates. (D) Motif analysis of the promoter region [file pbio.2006347.s002.pdf]

A

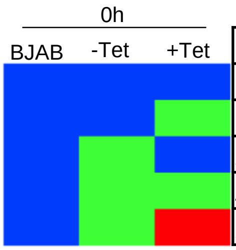

Pattern	Clone1 (normalized reads>10)	Clone2 (normalized reads>10)	Clone1(fdr<0.05)	Clone2(fdr<0.05)
1	9517	10357	0	0
2	53	64	41	43
3	38	33	26	18
4	7701	5583	6329	4424
5	163	66	159	65

B

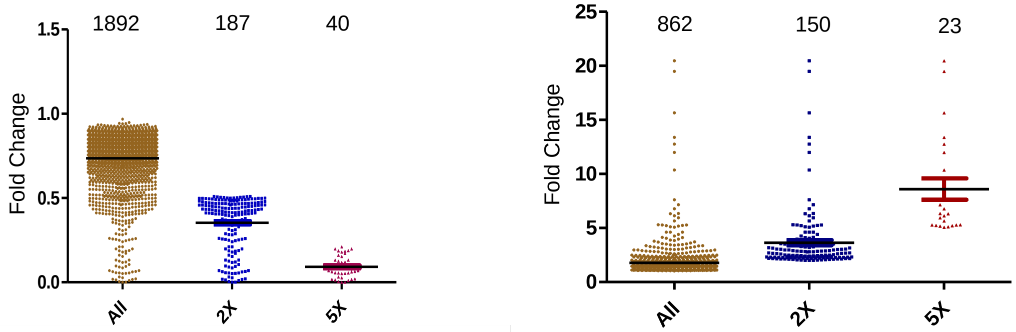

C

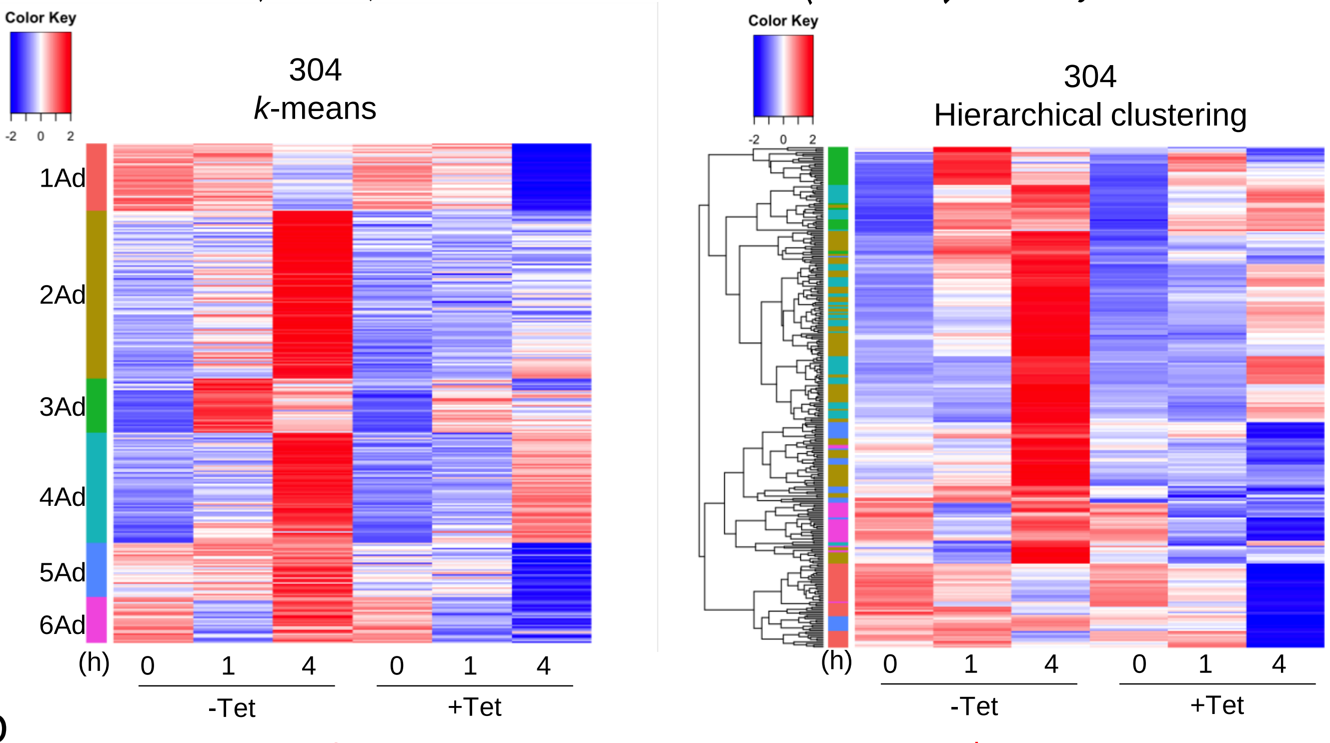

D

RELA binding, dnIkBα↓(304) -promoters

Consensus sequence	P-value	Motif Name
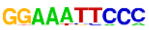	1e-13	NFkB-p65-Rel
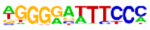	1e-10	NFkB-p65
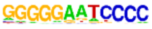	1e-6	NFkB-p50,p52

RELA binding, dnIkBα↓(304) -under peaks

Consensus sequence	P-value	Motif Name
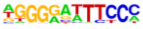	1e-129	NFkB-p65
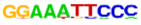	1e-68	NFkB-p65-Rel
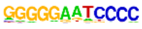	1e-61	NFkB-p50,p52

E

Under peaks (304)

Cluster number	Consensus sequence	P-value	Motif Name	% of Targets Sequences with Motif
Patterns 2Ad,4Ad	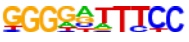	1e-69	REL	33.73%
	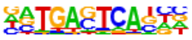	1e-27	JunB(bZIP)	28.99%
	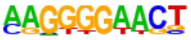	1e-18	Sfpi1	10.65%
Pattern 3Ad	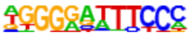	1e-16	NFkB-p65	42.42%
	bZIP motif	NA	NA	NA

F

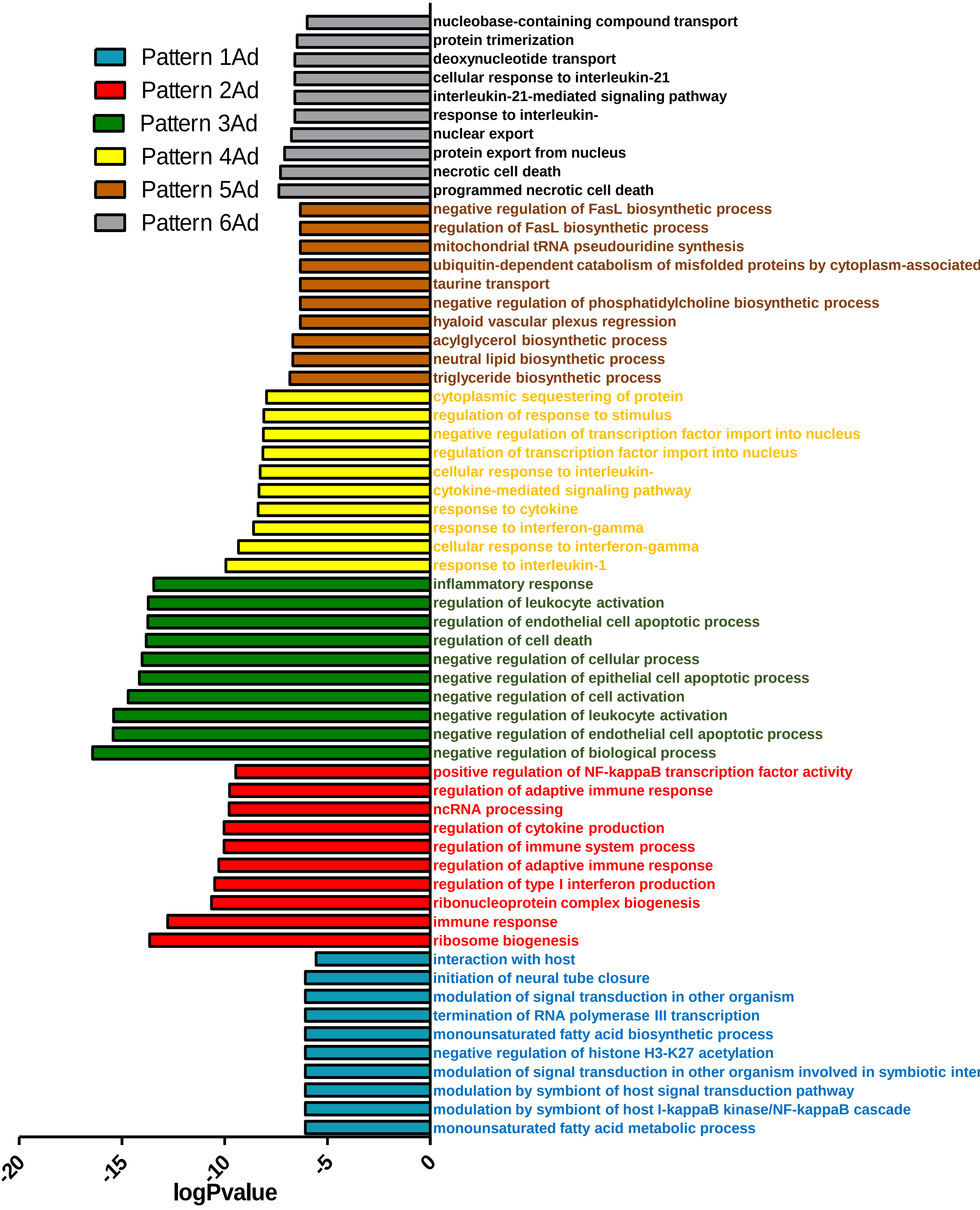
Pattern 1Ad
Pattern 2Ad
Pattern 3Ad
Pattern 4Ad
Pattern 5Ad
Pattern 6Ad
nucleobase-containing compound transport
protein trimerization
deoxynucleotide transport
cellular response to interleukin-21
interleukin-21-mediated signaling pathway
response to interleukin-
nuclear export
protein export from nucleus
necrotic cell death
programmed necrotic cell death
negative regulation of FasL biosynthetic process
regulation of FasL biosynthetic process
mitochondrial tRNA pseudouridine synthesis
ubiquitin-dependent catabolism of misfolded proteins by cytoplasm-associated
taurine transport
negative regulation of phosphatidylcholine biosynthetic process
hyaloid vascular plexus regression
acylglycerol biosynthetic process
neutral lipid biosynthetic process
triglyceride biosynthetic process
cytoplasmic sequestering of protein
regulation of response to stimulus
negative regulation of transcription factor import into nucleus
regulation of transcription factor import into nucleus
cellular response to interleukin-
cytokine-mediated signaling pathway
response to cytokine
response to interferon-gamma
cellular response to interferon-gamma
response to interleukin-1
inflammatory response
regulation of leukocyte activation
regulation of endothelial cell apoptotic process
regulation of cell death
negative regulation of cellular process
negative regulation of epithelial cell apoptotic process
negative regulation of cell activation
negative regulation of leukocyte activation
negative regulation of endothelial cell apoptotic process
negative regulation of biological process
positive regulation of NF-kappaB transcription factor activity
regulation of adaptive immune response
ncRNA processing
regulation of cytokine production
regulation of immune system process
regulation of adaptive immune response
regulation of type I interferon production
ribonucleoprotein complex biogenesis
immune response
ribosome biogenesis
interaction with host
initiation of neural tube closure
modulation of signal transduction in other organism
termination of RNA polymerase III transcription
monounsaturated fatty acid biosynthetic process
negative regulation of histone H3-K27 acetylation
modulation of signal transduction in other organism involved in symbiotic inter
modulation by symbiont of host signal transduction pathway
modulation by symbiont of host I-kappaB kinase/NF-kappaB cascade
monounsaturated fatty acid metabolic process
-20
-15
-10
-5
0
logPvalue

G

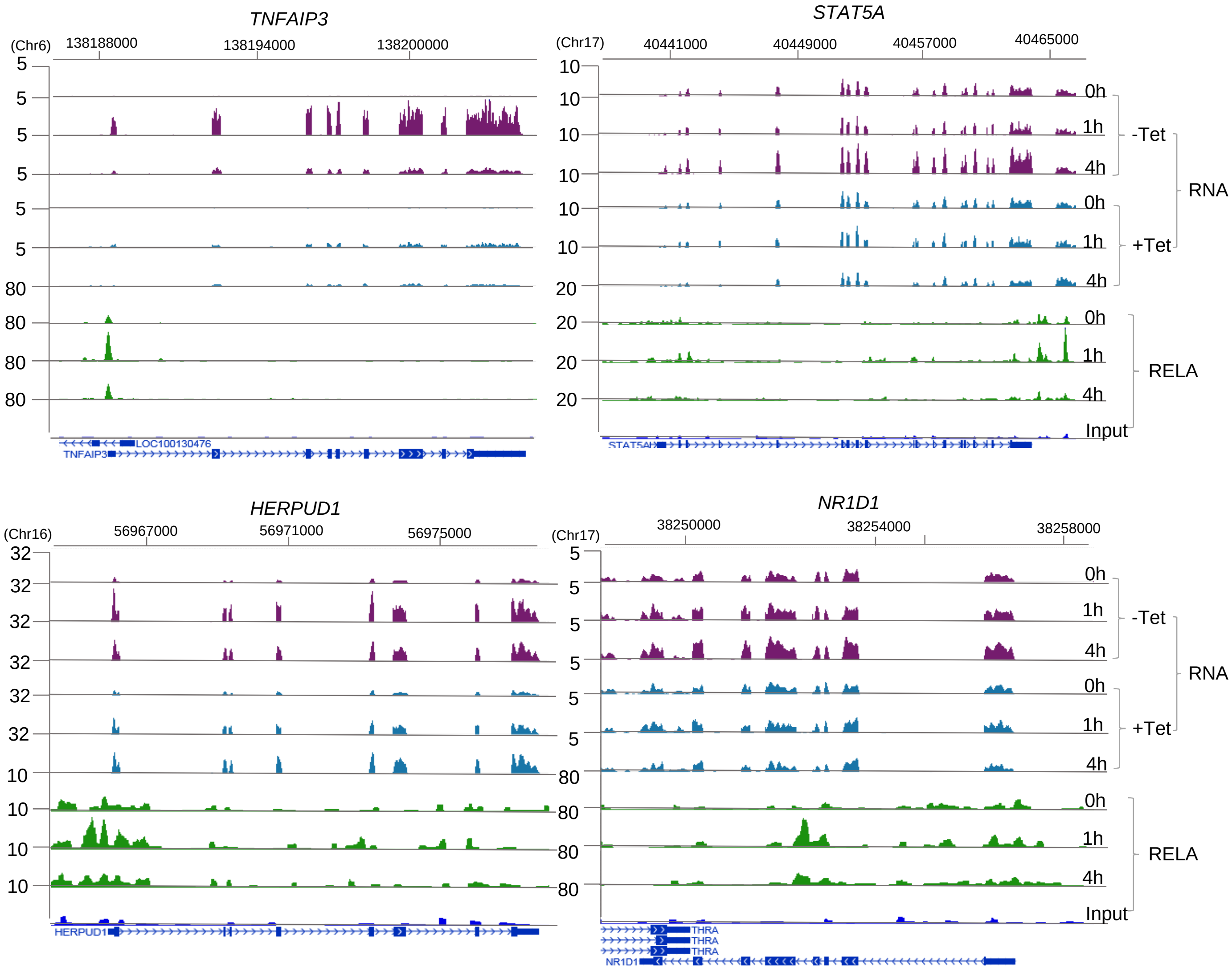
TNFAIP3
(Chr6)
138188000
138194000
138200000
LOC100130476
TNFAIP3
STAT5A
(Chr17)
40441000
40449000
40457000
40465000
STAT5A
HERPUD1
(Chr16)
56967000
56971000
56975000
HERPUD1
NR1D1
(Chr17)
38250000
38254000
38258000
THRA
THRA
THRA
NR1D1
0h
1h
4h
-Tet
+Tet
RNA
RELA
Input
