# Supplementary material for: Transcriptional outcomes and kinetic patterning of gene expression in response to NF-κB activation
Source: PLoS Biol. 2018 Sep 10;16(9):e2006347. doi: 10.1371/journal.pbio.2006347 (PMC6147668; doi:10.1371/journal.pbio.2006347)
Supplement: S3 Fig — Genes whose induction was reduced by dnIκBα expression but which did not bind RELA were considered to be indirect targets of RELA (see text). (A) Heatmap representation of 502 indirect RELA target genes identified by combining results of RNA-seq analysis in the presence or absence of dnIκBα with RELA ChIP-Seq (Fig 2C, left). Gene expression patterns were identified using k-means clustering (left and Fig 2C) or hierarchical clustering (right). Each column shows the level of expression in the absence of dnIκBα (3 lanes labeled -Tet) or the presence of dnIκBα (3 lanes labeled +Tet). Activation time course is indicated. Numbers to the left of the k-means heatmap correspond to patterns shown in Fig 2C (left). The letters “Ai” after a pattern number indicate target genes that were indirectly activated by RELA. Each column is the averaged expression from 2 biological replicate experiments. (B) Motif analysis of the promoter regions (−400 to +100 bp) of 502 indirect target genes was carried out using HOMER. The consensus sequence for MYC was the most enriched motif. (C) Browser tracks showing that the MYC gene is a RELA target in BJAB cells. (D) Genes encoding transcription factors were identified from RELA-activated (left) or RELA-repressed target genes (Figs 2 and 4). Databases used to identify genes that encode transcription factors were from a vertebrate transcription factor database (http://jaspar.genereg.net/downloads/), a human transcription factor list (http://www.tfcheckpoint.org/index.php/browse), and 2 GO groups (GO:0043565—sequence-specific DNA binding and GO:0003700—transcription factor activity, sequence-specific DNA binding). (E) RELA recruitment to identified target genes (ZNF267, HES1, and IRF1) was evaluated in activated BJAB cells by anti-RELA ChIP followed by qPCR (top 2 rows). NFKBIA gene was used as the positive control and H19 as the negative control. Results shown are the average of 2 independent ChIP experiments. Cells pretreated with Tet (+Tet) or [file pbio.2006347.s003.pdf]

A

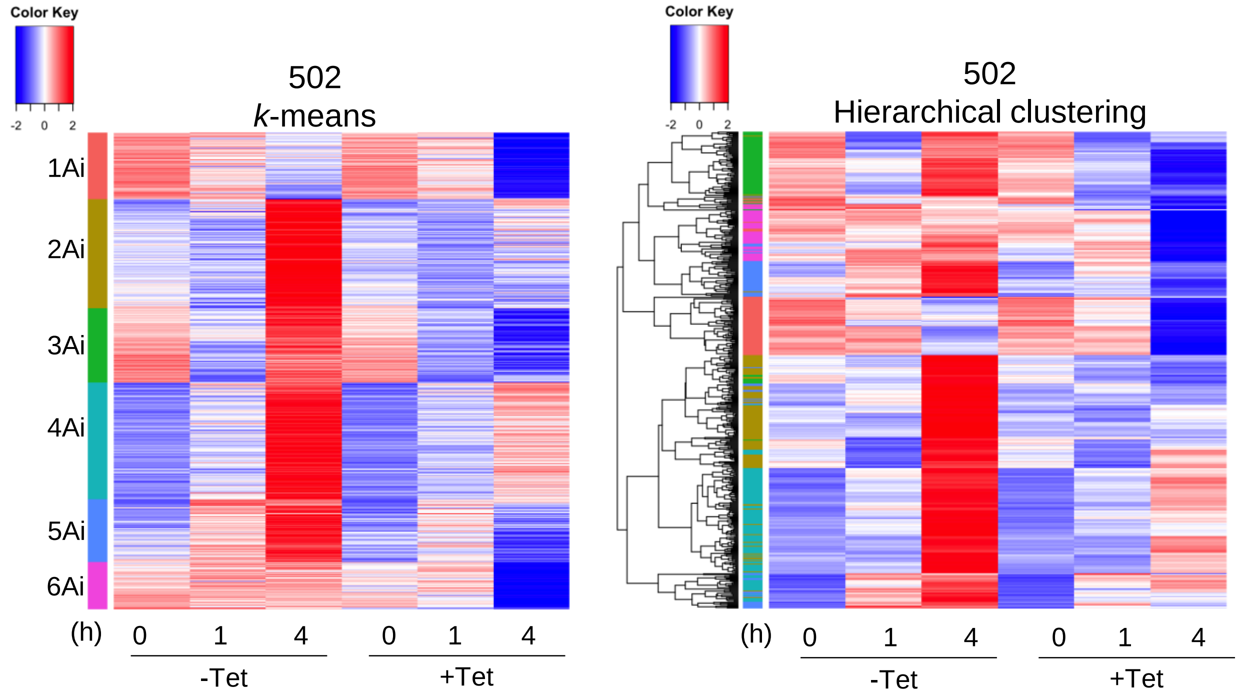

B

dnIkBα↓, no RELA binding(502)

Consensus sequence	P-value	Motif Name
CACGTG (logo)	1e-11	c-Myc

C

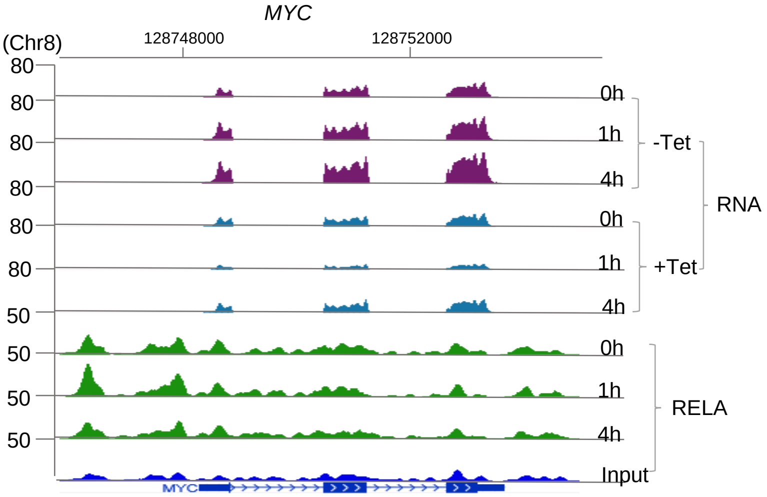

D

TF from 304

RUNX3	NCOA3	ELMSAN1	RELB
SPEN	SIN3A	STAT5A	BTG2
PRMT1	TP53	SNAPC4	NFKB1
STAT6	TAF4B	GPBP1	JUNB
TCF20	IRF1	GABPB1	KLF10
ZBTB2	ZBTB1	REL	HES1
DDN	POU2AF1	ZNF267	NFKBIZ
POU2F2	NR1D1	NFE2L2	
KDM2B	MYC	NFKB2	
ACTN4	UBTF	ZBTB10	

TF from 85

FOSB	BACH2	STAT3	MEF2C
FOS	BTG2	ZNF395	EGR1
NR4A3	DDIT3	ARID3A	SETX
NR4A1	IER2	MEF2B	AFF1
KLF6	RCOR1	TCF4	NCOA2
ZNF165	HBP1	TSC22D1	

E

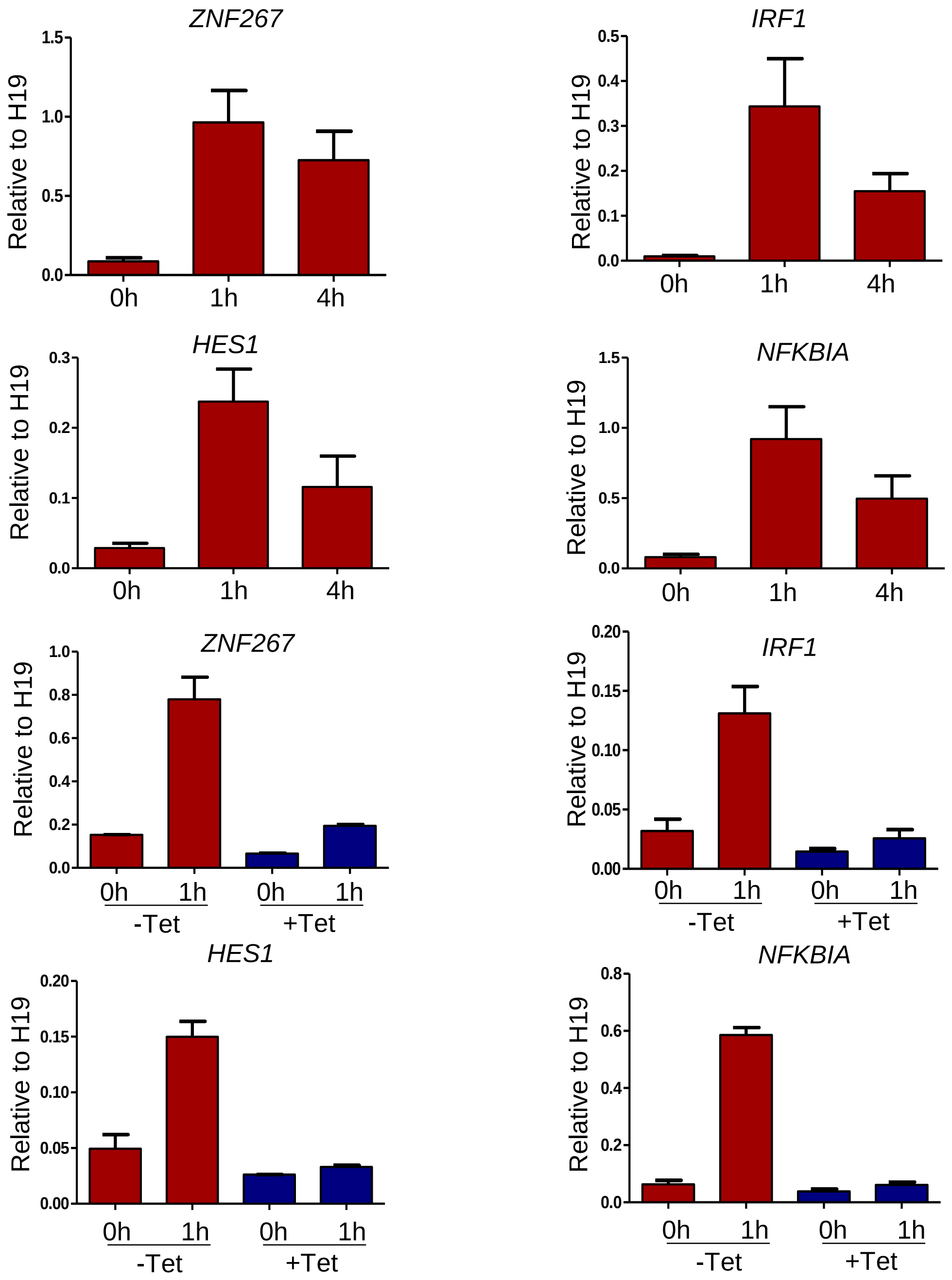

F

Motifs present in > 20% of promoters

Pitx1	Ptf1a	BMAL1	MITF	Isl1	n-Myc
KLF14	CRX	Nkx2.5	KLF3	Eomes	Arnt:Ahr
SCL	Erra	PU.1-IRF	Nkx3.1	c-Myc	ZFX
KLF5	Smad4	Nkx2.2	COUP-TFII	E2A	Elk4
AR-halfsite	Znf263	ETV1	Foxo1	Elk1	TATA-Box
THRb	Smad2	E2A	PR	AP-2gamma	KLF10
Smad3	NPAS	HIF-1b	ETS1	BMYB	Etv2
Maz	Egr1	E2F6	Meis1	AMYB	AP-2alpha
Nanog	Fli1	ERG	NF1-halfsite	Klf9	Ap4
Tgif2	ZNF416	Sp1	GABPA	E2F4	Rbpj1
Tbx5	Bapx1	ZNF467	Olig2	EHF	NPAS2
Tgif1	ZNF711	Ascl1	Nkx6.1	ELF1	GATA3
Nkx2.1	HEB	MYB	NFY	GSC	

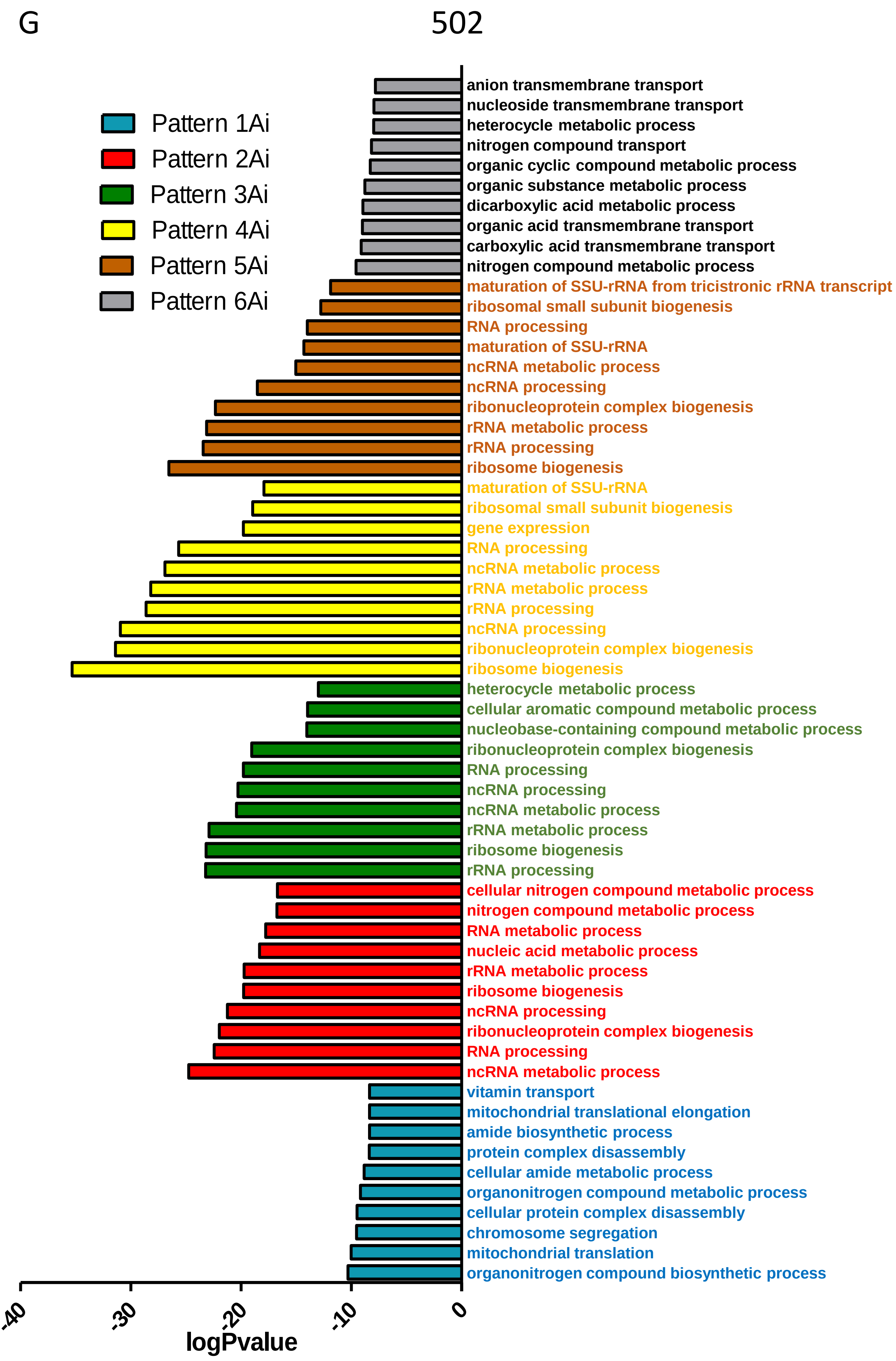
G
502
Pattern 1Ai
Pattern 2Ai
Pattern 3Ai
Pattern 4Ai
Pattern 5Ai
Pattern 6Ai
anion transmembrane transport
nucleoside transmembrane transport
heterocycle metabolic process
nitrogen compound transport
organic cyclic compound metabolic process
organic substance metabolic process
dicarboxylic acid metabolic process
organic acid transmembrane transport
carboxylic acid transmembrane transport
nitrogen compound metabolic process
maturation of SSU-rRNA from tricistronic rRNA transcript
ribosomal small subunit biogenesis
RNA processing
maturation of SSU-rRNA
ncRNA metabolic process
ncRNA processing
ribonucleoprotein complex biogenesis
rRNA metabolic process
rRNA processing
ribosome biogenesis
maturation of SSU-rRNA
ribosomal small subunit biogenesis
gene expression
RNA processing
ncRNA metabolic process
rRNA metabolic process
rRNA processing
ncRNA processing
ribonucleoprotein complex biogenesis
ribosome biogenesis
heterocycle metabolic process
cellular aromatic compound metabolic process
nucleobase-containing compound metabolic process
ribonucleoprotein complex biogenesis
RNA processing
ncRNA processing
ncRNA metabolic process
rRNA metabolic process
ribosome biogenesis
rRNA processing
cellular nitrogen compound metabolic process
nitrogen compound metabolic process
RNA metabolic process
nucleic acid metabolic process
rRNA metabolic process
ribosome biogenesis
ncRNA processing
ribonucleoprotein complex biogenesis
RNA processing
ncRNA metabolic process
vitamin transport
mitochondrial translational elongation
amide biosynthetic process
protein complex disassembly
cellular amide metabolic process
organonitrogen compound metabolic process
cellular protein complex disassembly
chromosome segregation
mitochondrial translation
organonitrogen compound biosynthetic process
-40
-30
-20
-10
0
logPvalue

H

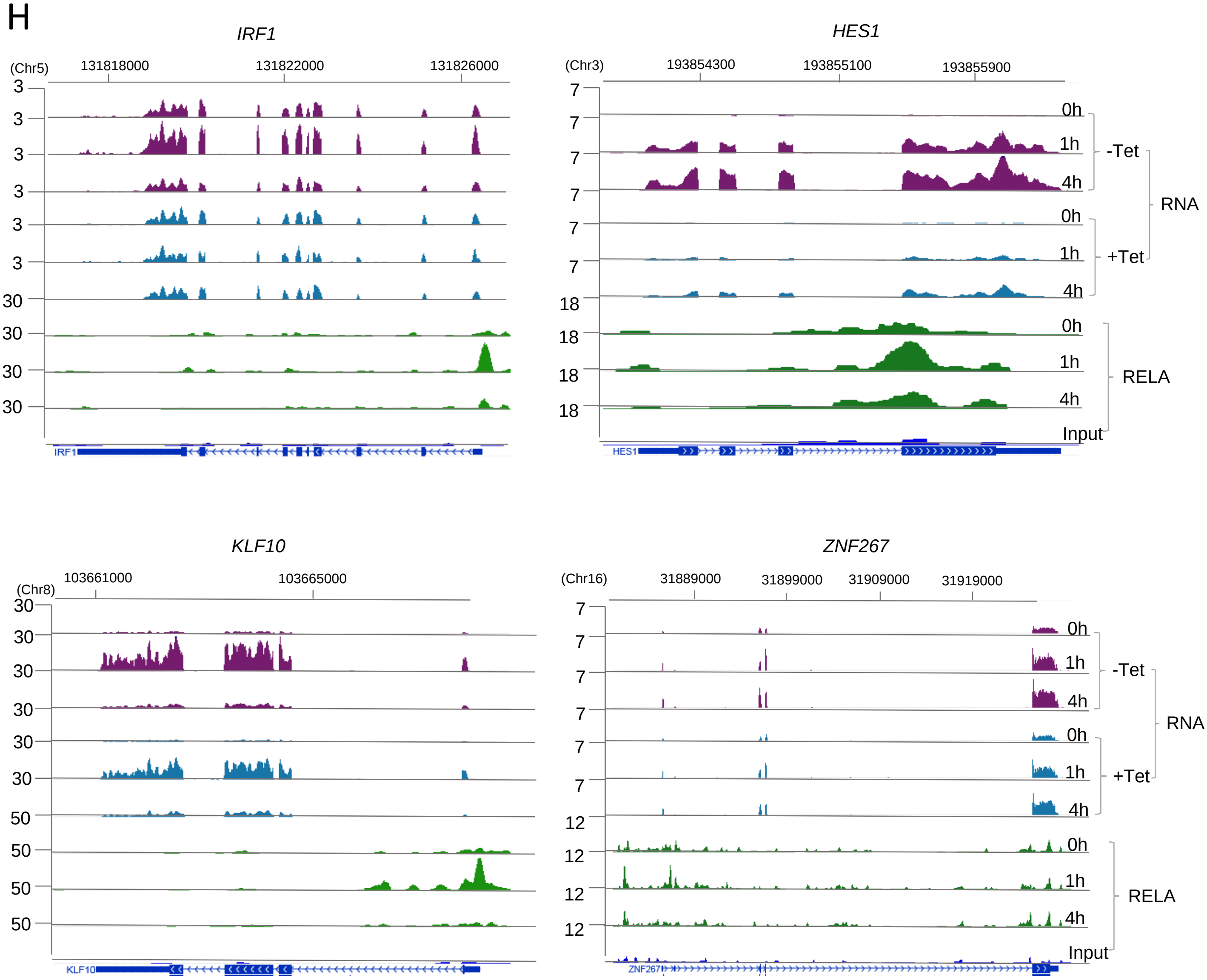

IRF1
(Chr5)
131818000
131822000
131826000
IRF1
HES1
(Chr3)
193854300
193855100
193855900
0h
1h
4h
-Tet
RNA
+Tet
RELA
Input
HES1
KLF10
(Chr8)
103661000
103665000
KLF10
ZNF267
(Chr16)
31889000
31899000
31909000
31919000
ZNF267
