# Supplementary material for: Transcriptional outcomes and kinetic patterning of gene expression in response to NF-κB activation
Source: PLoS Biol. 2018 Sep 10;16(9):e2006347. doi: 10.1371/journal.pbio.2006347 (PMC6147668; doi:10.1371/journal.pbio.2006347)
Supplement: S4 Fig — Such genes were considered to be repressed by inducible NF-κB in BJAB cells. Analogous to the discussion of inducibly activated genes, repressed genes that bound RELA were likely to be affected directly by RELA, whereas those that did not were considered to be indirectly affected (see text). (A) Heatmap representation of 85 direct RELA-repressed target genes (left) and 178 indirect RELA-repressed target genes (right) identified by combining results of RNA-seq analysis in the presence or absence of dnIκBα with RELA ChIP-Seq (Fig 4A). Gene expression patterns were identified using k-means clustering. Each column shows the level of expression in the absence of dnIκBα (3 lanes labeled -Tet) or the presence of dnIκBα (3 lanes labeled +Tet). The activation time course is indicated. Numbers to the left of the k-means heatmap correspond to patterns shown in Fig 4A. The letters “Rd” after a pattern number indicate target genes that were repressed by RELA binding, and the letters “Ri” after a pattern number indicate target genes that were repressed by RELA indirectly. Each column is the averaged expression from 2 biological replicate experiments. (B) RNA tracks showing complete time courses for RELA-repressed target genes shown in Fig 4B. (C) Motif analysis of DNA sequences under RELA peaks and promoter regions (−400 to +100 bp) of genes whose expression was increased by dnIκBα (FDR ≤ 0.05). AP1 and NF-κB motifs were prominent under RELA peaks; no significantly enriched motifs were identified in the promoters of these genes. (D) ngs.plot demonstrating the distribution of RELA binding near up-regulated target genes (130, from Fig 2B) and down-regulated target genes (53, from Fig 4A) whose inducible expression was changed ≥2-fold in the absence of Tet. Most RELA peaks were located at promoter regions of up-regulated target genes, while RELA peaks were distributed throughout the gene of down-regulated target genes. (E) Amongst genes whose inducible expression was increased by dn [file pbio.2006347.s004.pdf]

A

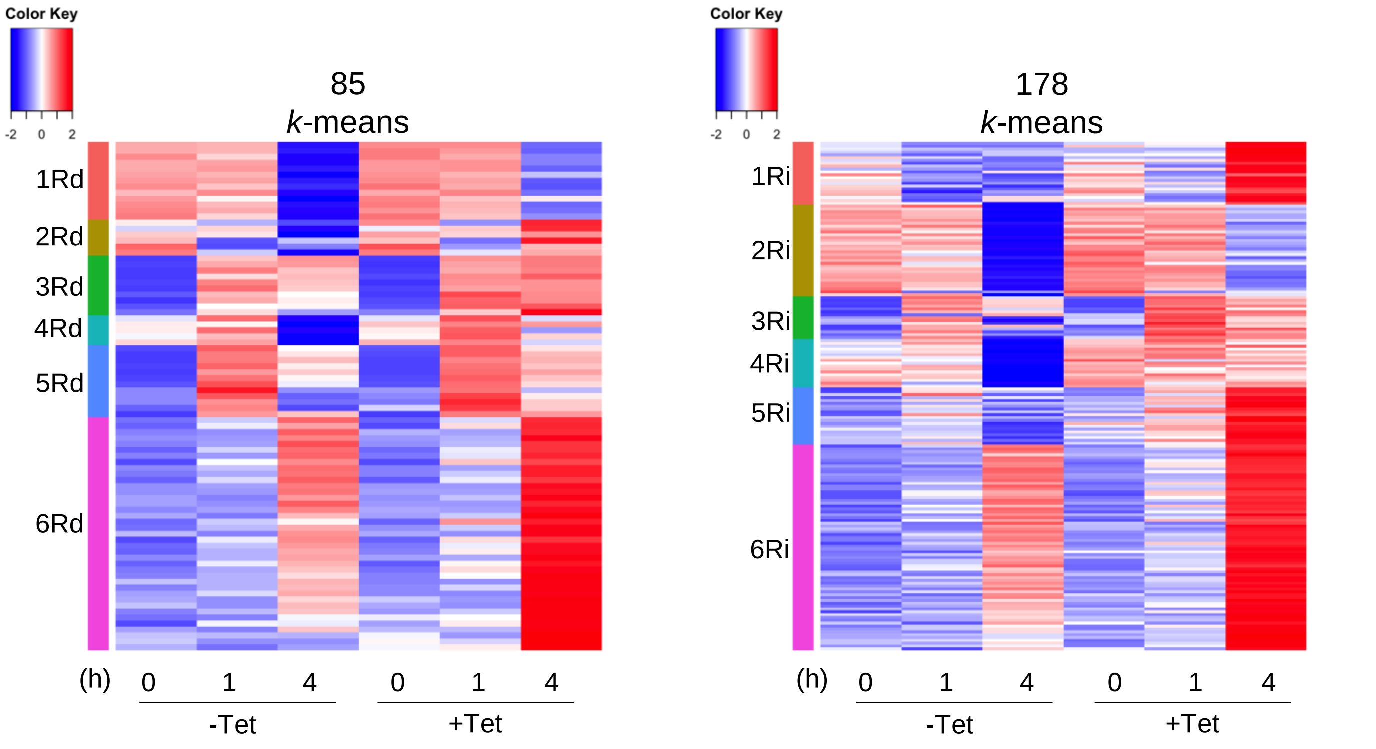

Color Key
-2 0 2
85
k-means
1Rd
2Rd
3Rd
4Rd
5Rd
6Rd
(h) 0 1 4 0 1 4
-Tet
+Tet
Color Key
-2 0 2
178
k-means
1Ri
2Ri
3Ri
4Ri
5Ri
6Ri
(h) 0 1 4 0 1 4
-Tet
+Tet

B

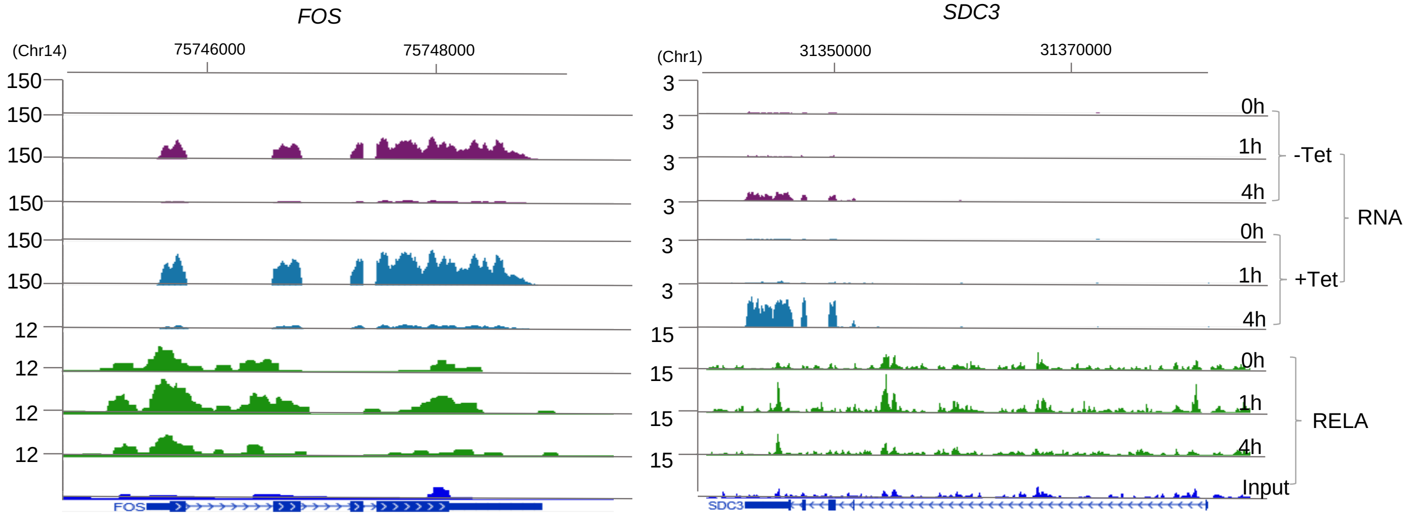

FOS
(Chr14)
75746000
75748000
150
150
150
150
150
150
12
12
12
12
FOS
SDC3
(Chr1)
31350000
31370000
3
3
3
3
3
3
15
15
15
15
0h
1h
4h
-Tet
0h
1h
4h
+Tet
RNA
0h
1h
4h
Input
RELA
SDC3

C

dnIkBα↑ , RELA peaks (85)

Consensus sequence	P-value	Motif Name
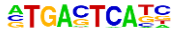	1e-17	AP.1
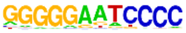	1e-9	NFkB-p50,p52
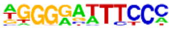	1e-9	NFkB-p65

Promoter regions: no significant motif

D

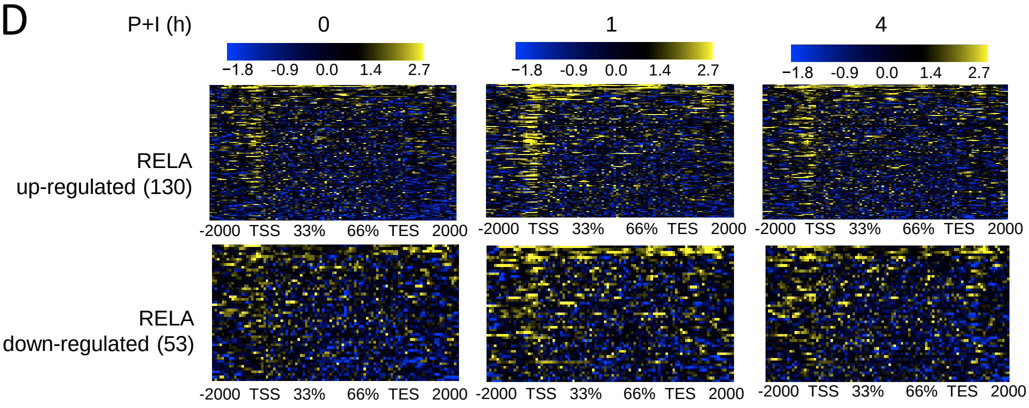

E

Motif present in >20% of promoters

KLF14	ERG	ZFX	MYB	NFY	FOXA1	NFAT
Maz	AR-halfsite	AP-2alpha	PR	Elk4	SPDEF	STAT4
SCL	CRX	Tgif1	Nkx6.1	Atoh1	Myf5	Lhx1
KLF5	Nkx2.5	Egr1	AMYB	Tcf12	c-Myc	Sox4
Pitx1	ETV1	BMYB	RXR	PPARE	E2A	Sox9
Nkx2.1	KLF3	BMAL1	PU.1-IRF	NPAS	Elk1	
THRb	Ptf1a	Ascl1	ETS1	E2F6	ELF1	
Znf263	AP-2gamma	KLF10	Klf9	GABPA	TATA-Box	
Nanog	Sox10	Ap4	MyoG	Isl1	Etv2	
Bapx1	HEB	Sox3	E2F4	EHF	Rbpj1	
ZNF711	Nkx3.1	Erra	EBF1	Tcf21	Klf4	
Nkx2.2	COUP-TFII	Smad4	Sox6	Atf1	Lhx3	
Smad3	Foxo1	Fli1	Smad2	MyoD	ZNF416	
Tgif2	Sp1	E2A	Meis1	Olig2	GSC	
Tbx5	ZNF467	HIF-1b	NF1-halfsite	Eomes	ELF5	

F

>20 percent have motif on promoters

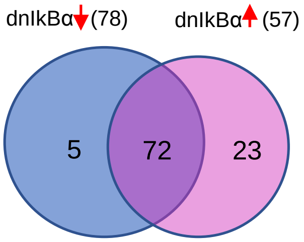

5

MITF
n-Myc
Arnt:Ahr
NPAS2
GATA3

23

Sox10	Atoh1	FOXA1	NFAT
Sox3	Tcf12	SPDEF	STAT4
RXR	PPARE	Myf5	Lhx1
MyoG	Tcf21	Klf4	Sox4
EBF1	Atf1	Lhx3	Sox9
Sox6	MyoD	ELF5	

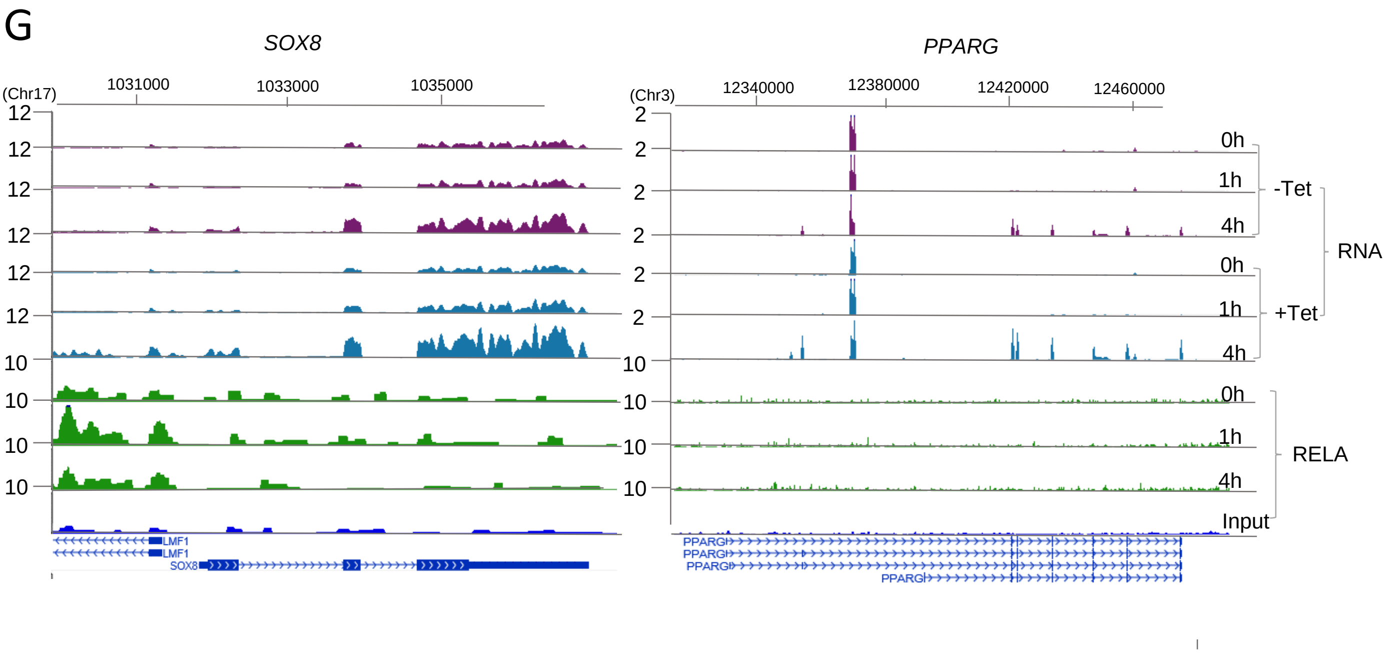
G
SOX8
PPARG
(Chr17)
1031000
1033000
1035000
(Chr3)
12340000
12380000
12420000
12460000
12
10
2
0h
1h
4h
-Tet
+Tet
RNA
RELA
Input
LMF1
SOX8
PPARG

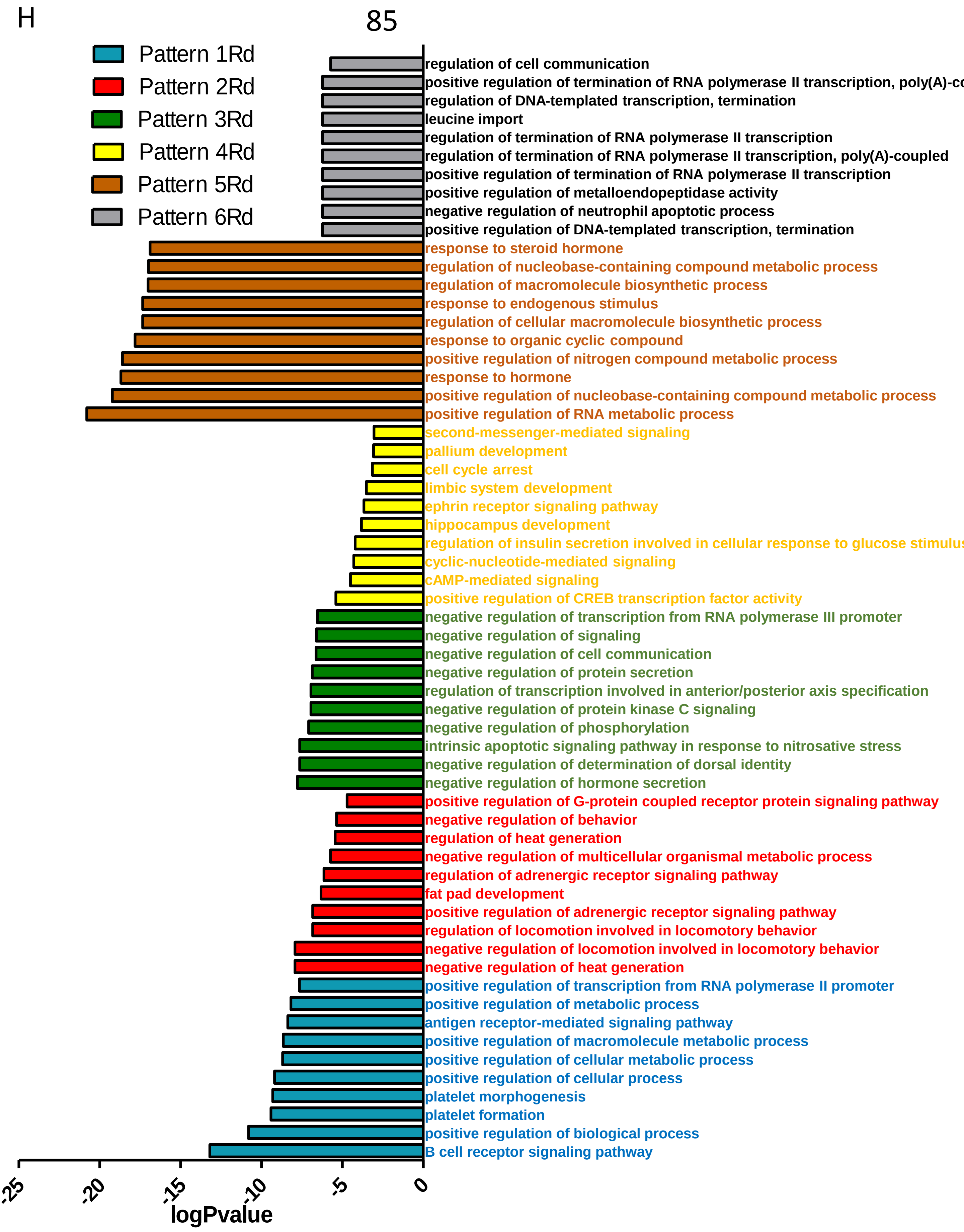

H
85
Pattern 1Rd
Pattern 2Rd
Pattern 3Rd
Pattern 4Rd
Pattern 5Rd
Pattern 6Rd
regulation of cell communication
positive regulation of termination of RNA polymerase II transcription, poly(A)-co
regulation of DNA-templated transcription, termination
leucine import
regulation of termination of RNA polymerase II transcription
regulation of termination of RNA polymerase II transcription, poly(A)-coupled
positive regulation of termination of RNA polymerase II transcription
positive regulation of metalloendopeptidase activity
negative regulation of neutrophil apoptotic process
positive regulation of DNA-templated transcription, termination
response to steroid hormone
regulation of nucleobase-containing compound metabolic process
regulation of macromolecule biosynthetic process
response to endogenous stimulus
regulation of cellular macromolecule biosynthetic process
response to organic cyclic compound
positive regulation of nitrogen compound metabolic process
response to hormone
positive regulation of nucleobase-containing compound metabolic process
positive regulation of RNA metabolic process
second-messenger-mediated signaling
pallium development
cell cycle arrest
limbic system development
ephrin receptor signaling pathway
hippocampus development
regulation of insulin secretion involved in cellular response to glucose stimulus
cyclic-nucleotide-mediated signaling
cAMP-mediated signaling
positive regulation of CREB transcription factor activity
negative regulation of transcription from RNA polymerase III promoter
negative regulation of signaling
negative regulation of cell communication
negative regulation of protein secretion
regulation of transcription involved in anterior/posterior axis specification
negative regulation of protein kinase C signaling
negative regulation of phosphorylation
intrinsic apoptotic signaling pathway in response to nitrosative stress
negative regulation of determination of dorsal identity
negative regulation of hormone secretion
positive regulation of G-protein coupled receptor protein signaling pathway
negative regulation of behavior
regulation of heat generation
negative regulation of multicellular organismal metabolic process
regulation of adrenergic receptor signaling pathway
fat pad development
positive regulation of adrenergic receptor signaling pathway
regulation of locomotion involved in locomotory behavior
negative regulation of locomotion involved in locomotory behavior
negative regulation of heat generation
positive regulation of transcription from RNA polymerase II promoter
positive regulation of metabolic process
antigen receptor-mediated signaling pathway
positive regulation of macromolecule metabolic process
positive regulation of cellular metabolic process
positive regulation of cellular process
platelet morphogenesis
platelet formation
positive regulation of biological process
B cell receptor signaling pathway
-25
-20
-15
-10
-5
0
logPvalue

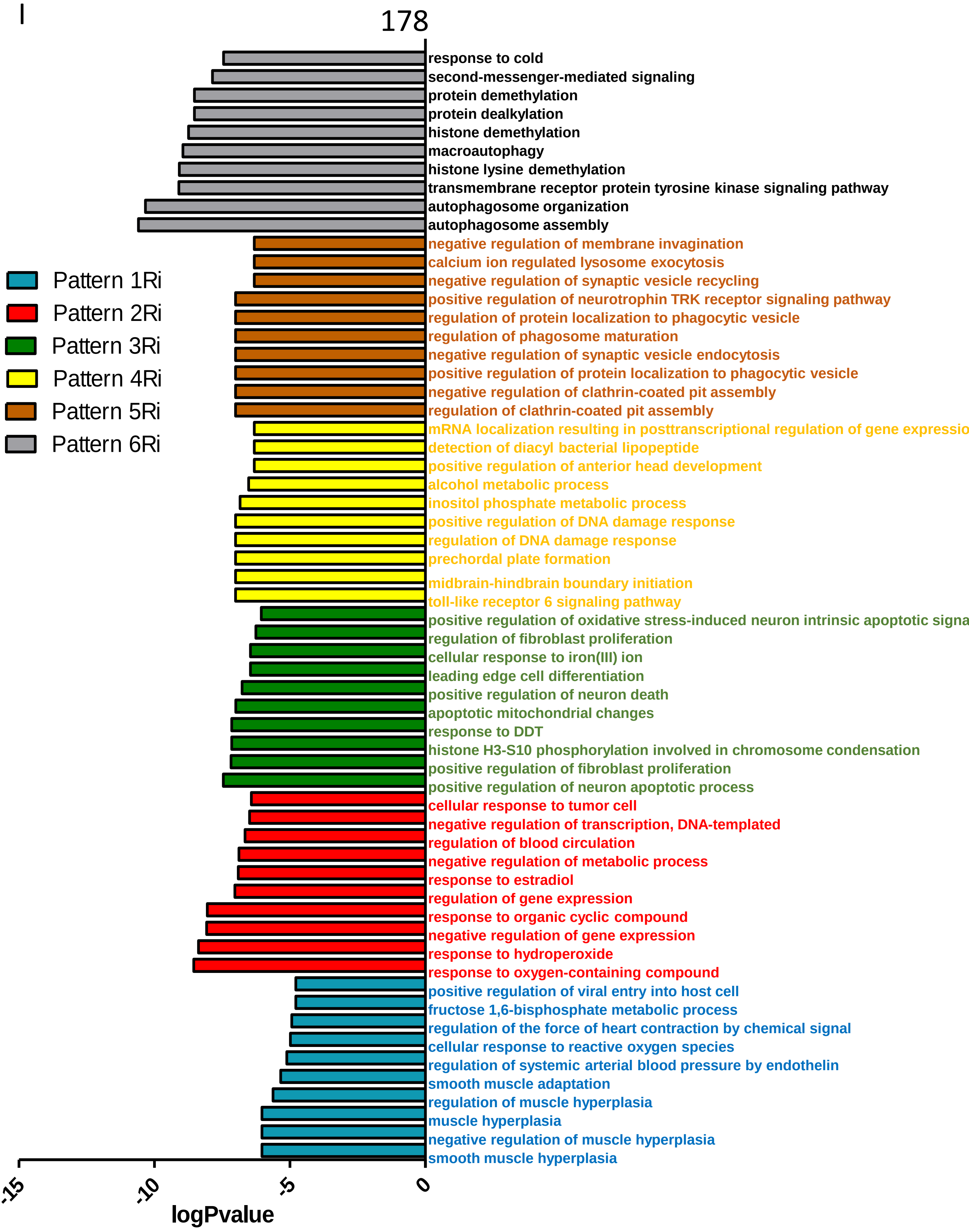
I
178
response to cold
second-messenger-mediated signaling
protein demethylation
protein dealkylation
histone demethylation
macroautophagy
histone lysine demethylation
transmembrane receptor protein tyrosine kinase signaling pathway
autophagosome organization
autophagosome assembly
negative regulation of membrane invagination
calcium ion regulated lysosome exocytosis
negative regulation of synaptic vesicle recycling
positive regulation of neurotrophin TRK receptor signaling pathway
regulation of protein localization to phagocytic vesicle
regulation of phagosome maturation
negative regulation of synaptic vesicle endocytosis
positive regulation of protein localization to phagocytic vesicle
negative regulation of clathrin-coated pit assembly
regulation of clathrin-coated pit assembly
mRNA localization resulting in posttranscriptional regulation of gene expressio
detection of diacyl bacterial lipopeptide
positive regulation of anterior head development
alcohol metabolic process
inositol phosphate metabolic process
positive regulation of DNA damage response
regulation of DNA damage response
prechordal plate formation
midbrain-hindbrain boundary initiation
toll-like receptor 6 signaling pathway
positive regulation of oxidative stress-induced neuron intrinsic apoptotic signa
regulation of fibroblast proliferation
cellular response to iron(III) ion
leading edge cell differentiation
positive regulation of neuron death
apoptotic mitochondrial changes
response to DDT
histone H3-S10 phosphorylation involved in chromosome condensation
positive regulation of fibroblast proliferation
positive regulation of neuron apoptotic process
cellular response to tumor cell
negative regulation of transcription, DNA-templated
regulation of blood circulation
negative regulation of metabolic process
response to estradiol
regulation of gene expression
response to organic cyclic compound
negative regulation of gene expression
response to hydroperoxide
response to oxygen-containing compound
positive regulation of viral entry into host cell
fructose 1,6-bisphosphate metabolic process
regulation of the force of heart contraction by chemical signal
cellular response to reactive oxygen species
regulation of systemic arterial blood pressure by endothelin
smooth muscle adaptation
regulation of muscle hyperplasia
muscle hyperplasia
negative regulation of muscle hyperplasia
smooth muscle hyperplasia
Pattern 1Ri
Pattern 2Ri
Pattern 3Ri
Pattern 4Ri
Pattern 5Ri
Pattern 6Ri
-15
-10
-5
0
logPvalue
