# Supplementary material for: Transcriptional outcomes and kinetic patterning of gene expression in response to NF-κB activation
Source: PLoS Biol. 2018 Sep 10;16(9):e2006347. doi: 10.1371/journal.pbio.2006347 (PMC6147668; doi:10.1371/journal.pbio.2006347)

A

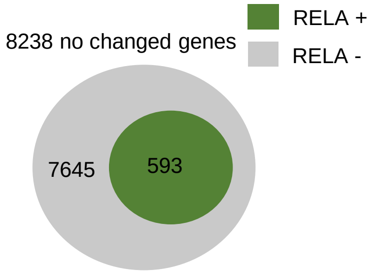

B

Under 593 RELA peaks

Consensus sequence	P-value	Motif Name
AGGGGATTTCCC	1e-89	NFkB-p65
GGAAATTCCC	1e-52	NFkB-p65-Rel
ATGACTCATC	1e-38	AP.1
GGGGGAATCCCC	1e-27	NFkB-p50,p52

Promoter regions: no significant motif

C

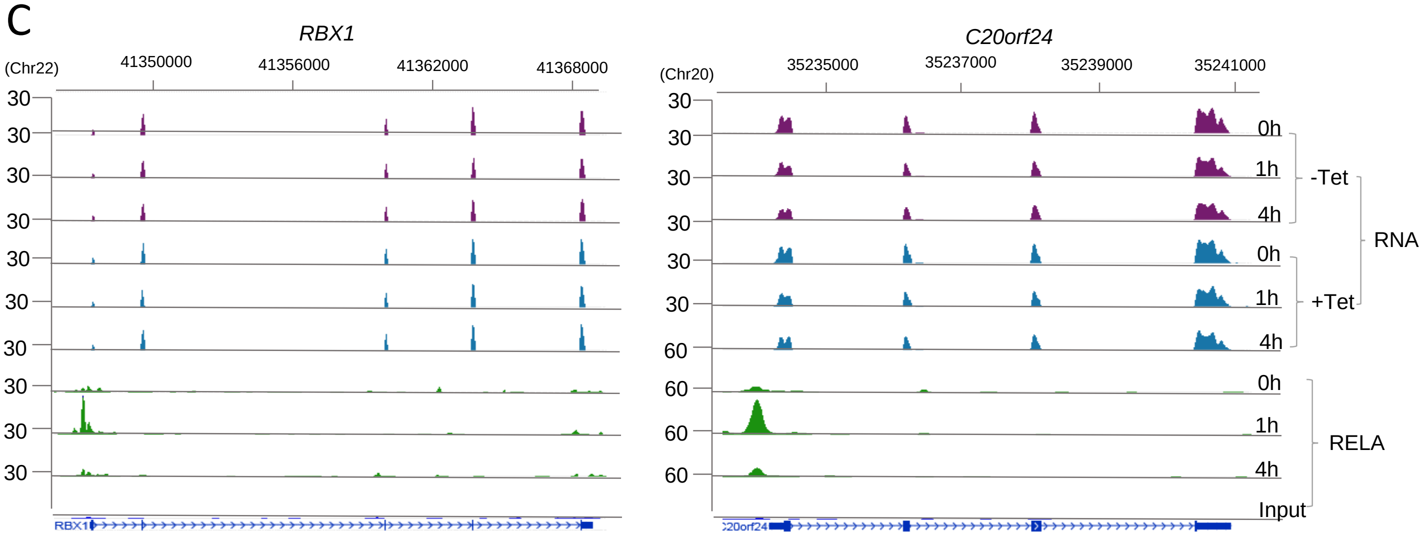

D

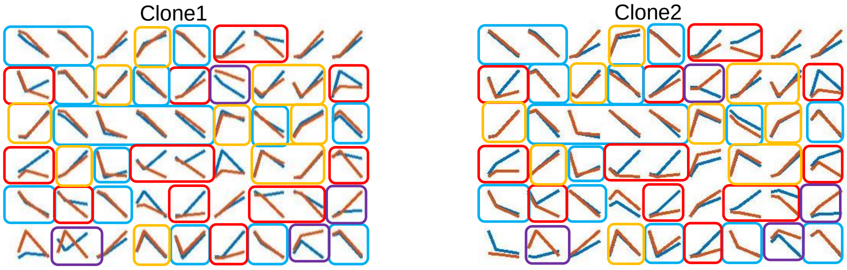

E

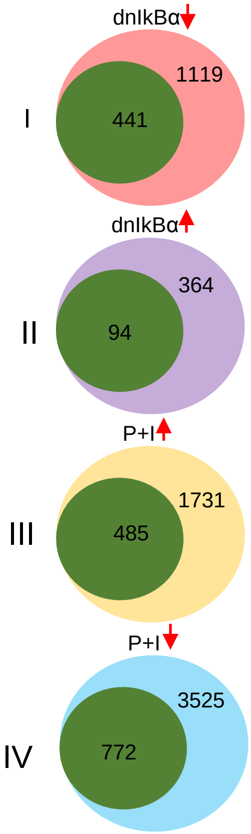

RELA peaks

Consensus sequence	P-value	Motif Name
AGGGGATTTCCC	1e-101	NFkB-p65
GGAAATTCCC	1e-69	NFkB-p65-Rel
GGGGGAATCCCC	1e-52	NFkB-p50,p52
ATGACTCATC	1e-43	AP.1

RELA negative promoters

Consensus sequence	P-value	Motif Name
GCCACGTGG	1e-11	c-Myc

Consensus sequence	P-value	Motif Name
ATGACTCATC	1e-45	AP.1
GGAAATTCCC	1e-43	NFkB-p65-Rel
AGGGGATTTCCC	1e-31	NFkB-p65
GGGGGAATCCCC	1e-28	NFkB-p50,p52

No significant motif

Consensus sequence	P-value	Motif Name
AGGGGATTTCCC	1e-53	NFkB-p65
ATGACTCATC	1e-43	AP.1
GGAAATTCCC	1e-28	NFkB-p65-Rel
GGGGGAATCCCC	1e-25	NFkB-p50,p52

Consensus sequence	P-value	Motif Name
AACCGGAAGT	1e-19	ETS
AGCCATTTCG	1e-10	YY1

Consensus sequence	P-value	Motif Name
AGGGGATTTCCC	1e-124	NFkB-p65
GGAAATTCCC	1e-73	NFkB-p65-Rel
GGGGGAATCCCC	1e-52	NFkB-p50,p52
ATGACTCATC	1e-37	AP.1

Consensus sequence	P-value	Motif Name
ACTTTCACTTTC	1e-4	ISRE(IRF)

F

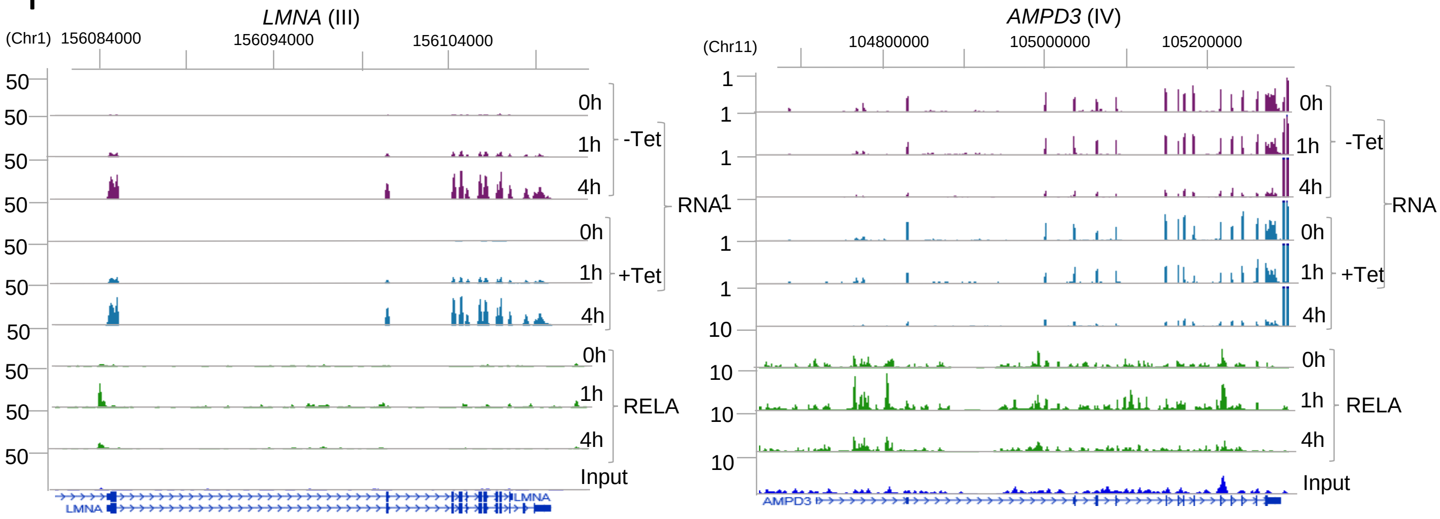

Supplement: S5 Fig — (A) RNA-Seq data from 2 biological replicates in each Tet-inducible clone were first used to define a set of genes whose expression did not change in response to cell activation and dnIκBα expression in both clones. We identified approximately 8,000 genes whose expression did not change with P+I treatment and dnIκBα expression. Of these, approximately 600 showed inducible RELA binding (green circle). (B) HOMER analysis of sequences underlying RELA peaks of these 600 genes identified authentic κB and AP1 motifs. No motifs were enriched at the promoters of these genes (−400 to +100 bp). (C) Examples of inducible RELA binding to genes that are unaffected by P+I treatment and dnIκBα expression. The top 6 lines show RNA-Seq tracks at different time points of P+I treatment and in the presence (+Tet) or absence (-Tet) of dnIκBα. The bottom 4 lines (including input track) show RELA ChIP-Seq tracks at different activation time points. (D) The remaining genes, whose expression changed in response to activation in both clones, were visualized by k-means clustering with correlation parameter in the absence (blue lines) or presence (red lines) of dnIκBα. Patterns were combined based on similar trends to generate 4 groups, which are indicated by colored boxes denoting the following characteristics: I (red boxes) = genes down-regulated by dnIκBα; II (purple boxes) = genes up-regulated by dnIκBα; III (yellow boxes) = genes up-regulated by P+I but unaffected by dnIκBα; and IV (blue boxes) = genes down-regulated by P+I but unaffected by dnIκBα. (E) RELA binding and promoter characteristics of genes in each group. Green circles denote RELA-binding genes identified by ChIP-Seq; gene numbers in each category are indicated. HOMER analysis was used to identify sequence motifs underlying RELA peaks (middle panel) or in the promoters (−400 to +100 bp relative to TSSs) of genes that did not bind RELA (right panel). (F) Representative examples of RNA expression and RELA binding to genes from [file pbio.2006347.s005.pdf]
