# Supplementary figures and images for: Transcriptional outcomes and kinetic patterning of gene expression in response to NF-κB activation
Source: PLoS Biol. 2018 Sep 10;16(9):e2006347. doi: 10.1371/journal.pbio.2006347 (PMC6147668; doi:10.1371/journal.pbio.2006347)

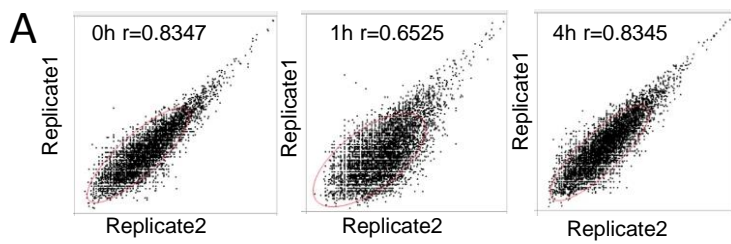

**B**

Polymerase II binding

|                             |    |
|-----------------------------|----|
| NF- $\kappa$ B target genes | 0h |
| Direct(130)                 | 50 |
| Indirect(78)                | 13 |

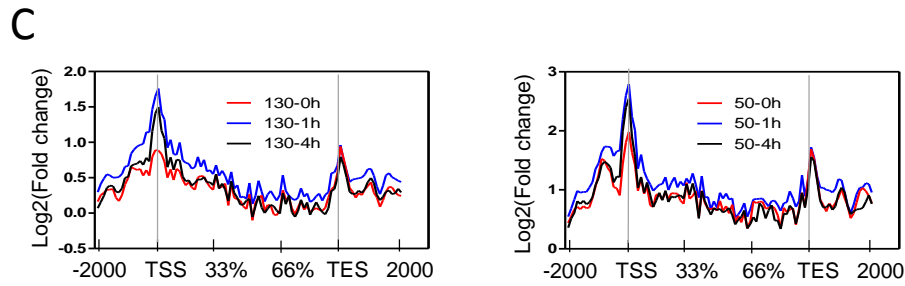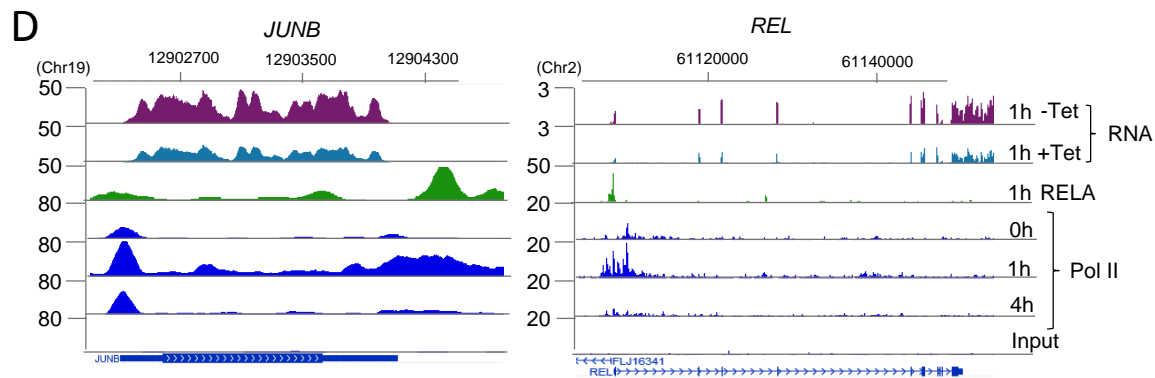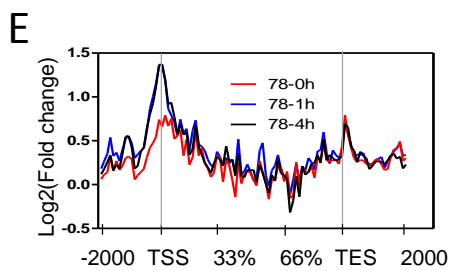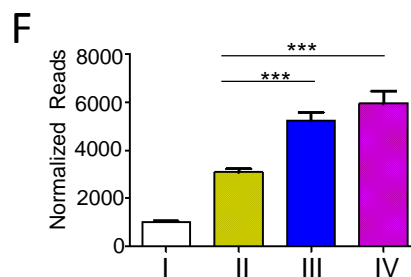

G

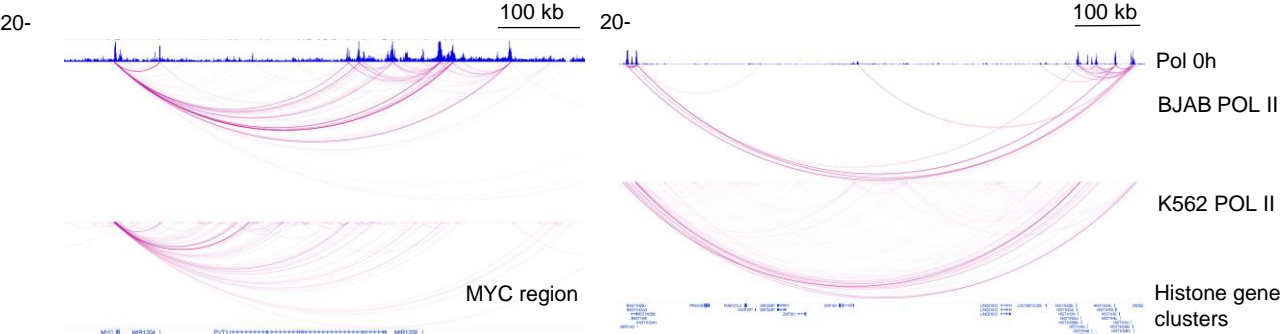

H

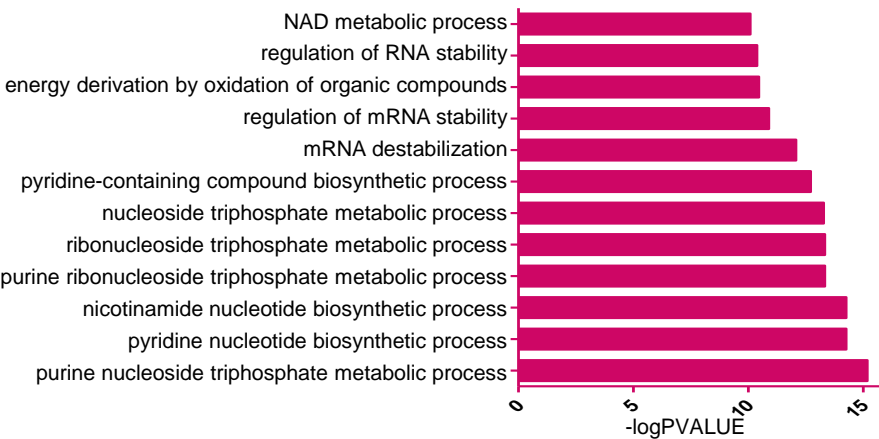

I

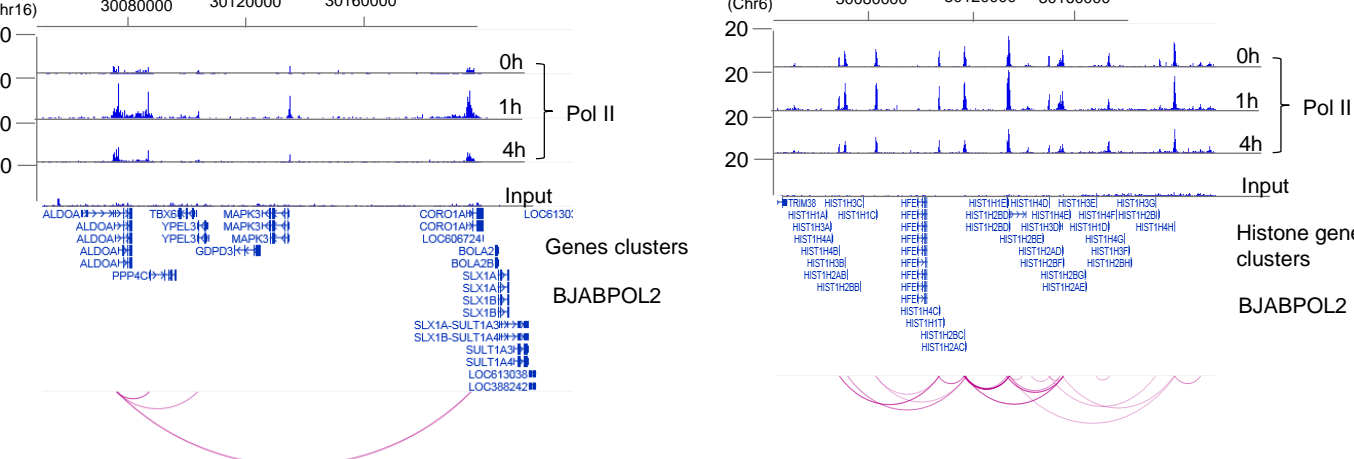

J

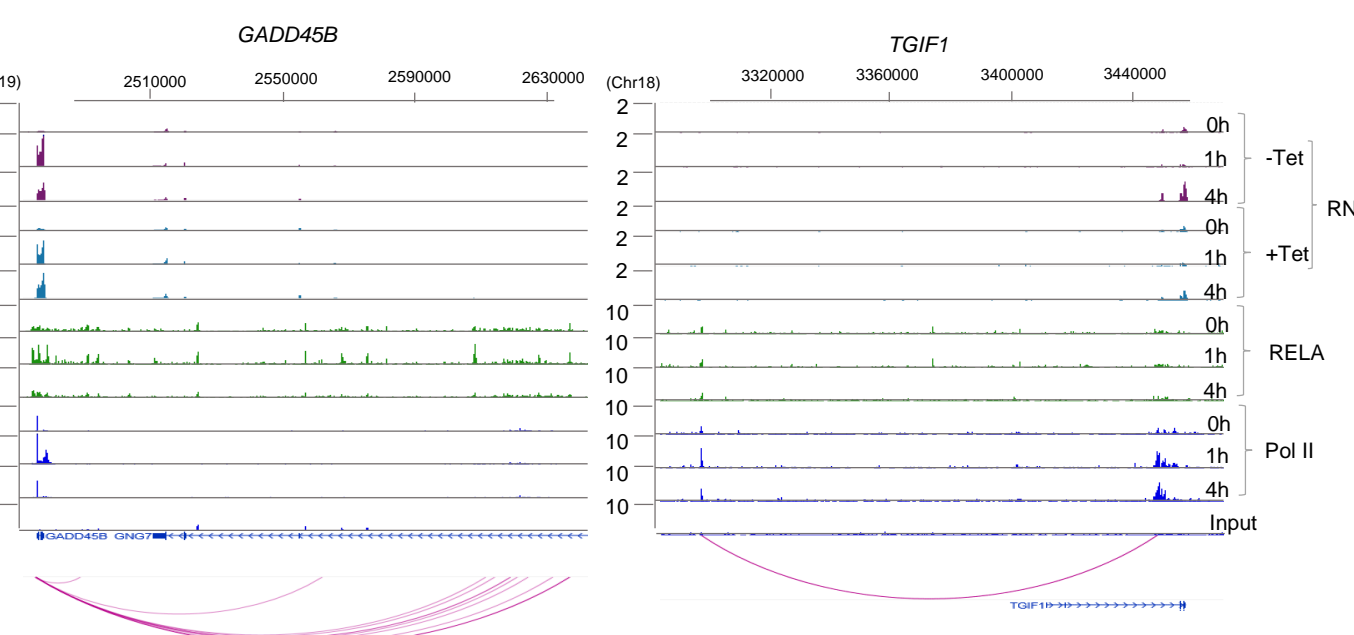

K

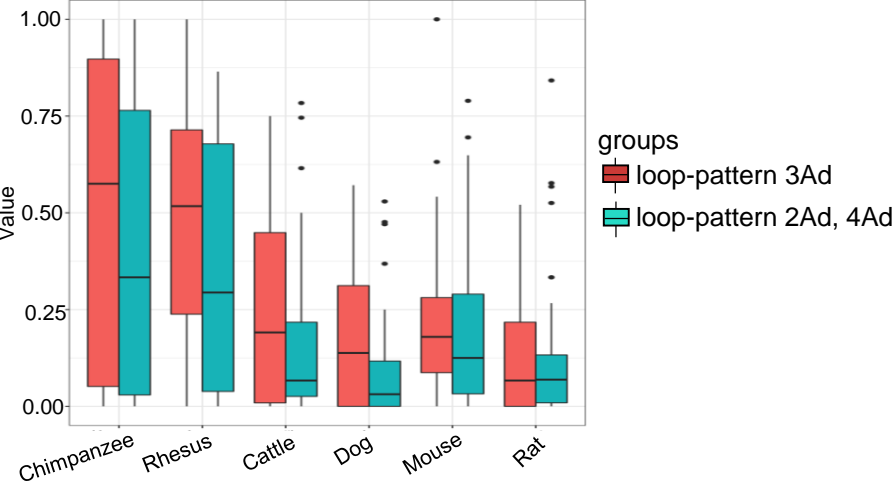

Supplement: S6 Fig — (A) Scatterplots depicting correlation between 2 replicates of Pol II ChIP-Seq for the indicated times. Further analyses were restricted to Pol II peaks with peak score ≥ 100 that were present in both biological replicates. Pol II ChIP-Seq data are available on the GEO website (http://www.ncbi.nlm.nih.gov/geo/) (Accession number GSE117259). (B) Pol II binding (0 h) to direct and indirect RELA target genes that are induced ≥2-fold by P+I in activated cells. The total number of genes in each category is noted in parentheses. (C) Pol II loading at 130 direct (induced ≥2-fold) RELA target genes as identified in Fig 2 shows recruited Pol II binding (left) after normalizing to gene length between annotated TSSs and TTSs. Tracks corresponding to different activation times are color-coded as indicated. Fifty out of 130 genes that have the pre-Pol II binding (S6B Fig) also show recruited Pol II binding (right). (D) Browser tracks of genes showing inducible Pol II recruitment in response to cell activation. The top 2 tracks show RNA-Seq tracks in the presence or absence of tetracycline-induced dnIκBα at 1 h. The center track shows the RELA ChIP-Seq track in BJAB cells at 1 h. The bottom tracks show Pol II ChIP-Seq in BJAB cells activated for the indicated times. (E) Pol II loading at 78 “indirect” (induced ≥2-fold) target genes as identified in Fig 2 is shown after normalizing to gene length between annotated TSSs and TTSs. Tracks corresponding to different activation times are color-coded as indicated. (F) RNA expression at baseline (in the absence of P+I) for genes in different ChIA-PET categories from Fig 5A. Genes with single-gene-based (Category III) or multiple-gene-based (Category IV) loops have higher RNA levels compared to genes that bind Pol II but do not display looping interactions (Category II). Underlying data for this figure are provided in S1H Data. Only the genes expressed in BJAB cells were used for statistical calculation. Statistical significance was teste [file pbio.2006347.s006.pdf]
