# Supplementary material for: Transcriptional outcomes and kinetic patterning of gene expression in response to NF-κB activation
Source: PLoS Biol. 2018 Sep 10;16(9):e2006347. doi: 10.1371/journal.pbio.2006347 (PMC6147668; doi:10.1371/journal.pbio.2006347)
Supplement: S1 Table — Transcriptional response of BJAB cells to P+I was assessed by RNA-Seq after 1 and 4 h of activation. Differentially expressed genes were identified by EBSeq [55] (Fig 1A). RELA binding over the same time course was assessed by ChIP-Seq. RELA-bound regions with peak score ≥ 100 that were reproduced in biological replicates were used for all analyses. RELA binding was ascribed to a specific gene according to HOMER using the default parameters (http://homer.ucsd.edu/homer/). For most of these genes, RELA bound at the promoter, within the gene body, or within 50 kb of the transcription start site. RELA-binding genes that were up-regulated ≥2-fold at either 1 or 4 h are shown in A. RELA-binding genes that were down-regulated ≥2-fold at either 1 or 4 h are shown in B. ChIP-Seq, chromatin immunoprecipitation and sequencing; P+I, phorbol 12-myristate 13-acetate and ionomycin; RNA-Seq, RNA sequencing. (PDF) [file pbio.2006347.s007.pdf]

A

CCL3L1	ZNF165	ZMIZ1-AS1	CTD-2541M15.1	SLCO2B1	NPIPB3	KATNBL1	PACSIN2	CSTB	DMWD	SLC12A8	CTC-250I14.6	NPHP4
SNX9	MAK	DFNB31	SEMA7A	METRNL	LINC00963	ZNF8	SLC7A1	THAP4	DYNC2H1	RP1-228H13.5	RP11-642P15.1	MDM4
FOSB	LIF	APOBEC3B	CRYM	ABTB2	NDRG4	MYBPC1	MYO1C	SLC16A6	ANKRD28	KIF9	IL2RB	CDKN2B-AS1
LINC01010	NEDD4L	IGF2R	CTD-2184D3.5	GALNT2	RFX2	ZBTB43	GABPB1	OTUD4	RAB7A	LBH	ARHGAP44	TOB1
ZNF365	RGS1	NAALADL2-AS2	RP11-574H6.1	OR7E47P	BTBD3	SLC35E4	SCIMP	C16orf72	WEE1	ITGB3	ZNFX1	TSC22D2
PPY	RRN3P1	ZFP36L1	PHACTR1	MYEOV	TRIM36	MAML2	BIRC7	IRF2BP2	SLC1A5	ZSWIM6	WARS	RP11-481J2.2
SLC22A1	MIR155HG	FAM102A	NT5DC4	TTLL10	RBKS	TRIB1	RHOG	INSIG1	KSR1	USP12	ATP2B1	GLA
EGR3	BHLHE41	NPIPB11	PLEKHA7	LINC-PINT	C16orf46	JAM2	LINC00936	WDR66	RASL11A	LINC00355	RP11-458D21.1	CCL4L1
CHRNA1	TRPV3	LUCAT1	RP11-672A2.6	ZFP69B	SACS	RIN3	RAB11FIP1	ITPKB-IT1	FGFRL1	ATF5	NBL1	
ANKRD30B	ZBTB32	BHLHE40	TNS1	RHOBTB3	GAS7	ITGAD	RAB30	ABR	SPPL2A	RP5-864K19.4	RP11-638I2.8	
MTSS1	MYADM	S100A2	RP11-540A21.2	B3GNT7	KLHL21	DNAAF2	CD70	ELMSAN1	TMEM2	UBE2B	CDYL2	
CORO6	GJA3	LINC00152	RP11-465B22.3	ZNF554	PPL	ID1	TSNARE1	TNIP2	ZNF778	KIAA1199	RNF103-CHMP3	
SLAMF7	PBX4	IL36RN	LINC00511	LINC01060	CARS	SQLE	TCF7	ASIC3	NRROS	HSP90B1	SUN1	
LINC00702	ELL2	IL36B	AC003092.1	WDFY1	NEURL1	NOTCH2NL	LASP1	CLTC	SLC9A1	RP11-116N8.4	NSMAF	
BACH2	LRRC32	WNT9A	CABLES1	SETX	DUSP22	PYGO1	BACH1	GPBP1	CD97	CTD-2516F10.2	ZCCHC14	
CD163	APCDD1L-AS1	CD109	CTD-2325P2.3	TIPARP	MATN1	SERPINA9	CLEC17A	GFOD1	TMEM178B	KCP	BIN2	
LY9	LDLRAD4	CTD-2532D12.4	TVP23A	RILPL2	AMZ2P1	NOTCH2	LRCH1	FAM222A	WDR86	ADORA2A-AS1	C19orf26	
HES1	DDIT3	TENM4	HRK	SDC3	RRAGC	UBALD2	CCDC64	BAIAP3	RP3-330M21.5	CTNNA1	ZSWIM4	
AHNAK	IL6R	RP11-20J15.2	RP3-468K18.5	KIF25-AS1	MAP1LC3B	EPB41L2	SUB1	TNFRSF14	ITK	RP4-781K5.2	FNDC3B	
GDF15	C5AR1	SLC30A10	NAALADL2	FNIP1	UBTD2	C7orf72	GARS	AP5Z1	MATN1-AS1	LAMP3	RHEB	

Supplementary Table 1

B

PTPRS	SNX29P2	NCOA2	KREMEN2	ALDH3B2	FADS2	PLEKHG1	ATXN1	ABI3
ACE	RNF135	RXRA	HDAC7	JADE2	CTSZ	RP4-798A10.7	CD79B	ELL3
PSTPIP1	PLCG2	VAV1	MAML3	BZRAP1-AS1	PARP10	AC009005.2	FOXI1	RP11-111A22.1
EHBP1L1	MAP3K7CL	BTN3A2	SIPA1L3	ASB13	CCDC106	RP11-255H23.2	NEURL2	RP11-571M6.15
NARFL	KAZALD1	TP53I11	IGLL5	PRIM1	BCL11A	CTA-126B4.7	SLC28A3	CTD-2545G14.7
NEIL1	SYVN1	ASB2	PROC	UBTF	DBP	OSBPL10	SPATA12	
TREX1	CUEDC1	TMCC2	SH2D3C	RP11-386G11.5	RP11-16K12.1	C2ORF15	PLEKHA2	
COASY	FAM213B	TTC9	PVRIG	BANK1	RP11-111M22.2	USP2	RP11-296A16.1	
NTHL1	PIK3C2B	PTK2B	PDGFD	HSH2D	PCDHGB2	SH2B2	PCDHGC4	
FGD2	MTMR12	MEF2B	PIK3CD	RP11-640M9.1	RHOBTB2	PMEPA1	IFNG-AS1	
ZNF395	DLG2	CBLN2	EPB41L1	RAB11FIP4	HHEX	CORO2B	WNK2	
C21orf33	PPP1R18	MEF2C	KCNN3	STX10	ELOVL6	ADAMTSL2	LPP-AS2	
ZNF860	SORL1	TTC23	PLXNC1	HECW2	ARHGEF3	CCDC102A	TOB1-AS1	
IQSEC1	ABHD8	CNR1	DIXDC1	EPB41L4A	ZBTB14	CALHM3	PCDHGA10	
TCF4	LRRK1	TRIM8	SYK	DHRS13	Z95704.4	MGAT3	PTPN6	
RAC2	VAV3	GRAP	TSPAN18	MAMSTR	AC010761.9	ABCG1	CDC42BPB	
DTX1	PILRA	TBX10	ZBTB12	HSD17B6	NRM	ARHGAP23	PCDHGA12	
ZBTB7B	KBTBD3	PLEKHF2	EHD3	GALNT6	TMEM116	C1orf220	SH3PXD2A	
LMTK3	SYPL1	CCDC85C	KIAA0040	PIK3R2	AC096559.1	SIT1	LRP2	
CPNE5	CSPG4	NFIA	TSC22D1	SYNPO	NCKAP5	DOK7	CUX2	

Supplementary Table 1
