# Supplementary material for: Transcriptional outcomes and kinetic patterning of gene expression in response to NF-κB activation
Source: PLoS Biol. 2018 Sep 10;16(9):e2006347. doi: 10.1371/journal.pbio.2006347 (PMC6147668; doi:10.1371/journal.pbio.2006347)
Supplement: S2 Table — A total of 304 direct RELA target genes that were identified in this study based on inducible activation in response to P+I, RELA binding by ChIP-Seq, and down-regulation by dnIκBα. Genes marked in red were changed more than 2-fold in the absence of tetracycline. Genes marked in green are newly identified target genes not previously noted in a cumulative list culled from multiple databases (http://www.bu.edu/nf-kb/gene-resources/target-genes/) (https://www.yumpu.com/en/document/view/8327926/the-nfkb-target-gene-sets-are-listed-below-broad-institute) [24,27]. ChIP-Seq, chromatin immunoprecipitation and sequencing; dnIκBα, dominant negative NFKB inhibitor alpha; P+I, phorbol 12-myristate 13-acetate and ionomycin. (PDF) [file pbio.2006347.s008.pdf]

CCL3L1	SLC7A1	CCL4	STAT5A	IL2RG	MAPKAPK5	RCC2	TAPBP
SLC22A1	IRF2BP2	NFKBIZ	GCLC	SLC25A13	ARHGAP25	POLR3K	ORAI1
CCL4L1	DUSP22	NFKBID	CFLAR	MAML2	NXT1	PARP14	LPXN
SNX9	GAS7	CD83	MYC	PABPC4	ACTN4	LMNB2	IRF1
IL36RN	GABPB1	CACNA1E	SOD2	TRAF4	PRCC	SMPD4	SYNJ2
IL36B	LRCH1	SGK1	IFIH1	ZBTB1	BAZ1A	RQCD1	TAF4B
LIF	CSTB	EBI3		KIAA0247	TIPIN	SPATA13	HLA-B
MIR155HG	CBX6	TNFAIP3		NUP188	CCDC86	NUP62	THOC1
HES1	SERPINA9	PPP1R15A		CRCP	ATP1A3	DDX42	HLA-A
UTP3	TNIP2	PLAU		DEXI	PDSS1	VOPP1	BLM
NR1D1	RP11-481J2.2	IER3		GPX4	EMC1	TIMM13	NUP153
POU2AF1	GPBP1	DDX21		PTRH2	QTRTD1	GUCD1	NINJ1
PPAN	ZNF106	JUNB		RIOK1	NLGN4X	MOB3A	PPIF
MAK16	ARHGAP24	PIM1		LINC00877	DDN	ZBTB2	SLC6A6
CLEC17A	CTB-58E17.1	NFKB1		TNFRSF14	TCF20	SCD	SLC39A11
CIITA	SNAPC4	MAP2K3		IGF2BP3	SLC39A1	SLC25A19	TRAF3
UBTF	ZFP36L2	MAP3K8		CTD-2130O13.1	GNL3	FCRL3	SH2B3
CBLN2	ELMSAN1	BTG2		CHD4	LETM1	SH3BP2	POU2F2
KIF25-AS1	STK10	PIM3		GTF3C4	ASXL1	PGAM5	WDR4
NAALADL2	PLXNA1	GADD45B		PES1	MAN2B1	SCO2	PRMT1
RILPL2	NOTCH2	EMP3		ABCF1	STAT6	KDM2B	SPEN
PLEKHA7	IL27RA	NFKBIA		TOR3A	SLC35F2	MFNG	RUNX3
RP11-672A2.6	KLHL18	KLF10		SLC39A6	XPO6	XRCC2	SCARB1
NAALADL2-AS2	PDXK	ZC3H12A		N4BP3	RASAL2	TTLL12	ARHGAP17
APOBEC3B		DOT1L		TNPO2	ENTPD1	LARP1	TBC1D14
PHACTR1		RGS16		ARHGEF2	GRSF1	PISD	SIN3A
DNAAF2		NFKBIE		NOC3L	PUS1	AMER1	TAP1
SACS		MAPK6		STX6	NOLC1	ICAM2	TMEM97
ABTB2		RELB		REPIN1	SLC35E1	KREMEN2	TNIP1
OTUD4		IL10		TRIP13	TBL3	C10orf128	TRAF2
MYO1C		HERPUD1		LTV1	SNX29	IL21R	PPARGC1B
JAM2		ZBTB10		NLN	JAK3	CD3EAP	TP53
UBTD2		NFKB2		TTC27	METTL21A	BLOC1S4	
UBALD2		IL4I1		SYNGR2	LPCAT1	SURF6	
IGF2R		TRAF1		TRIT1	KIAA1217	POLR3H	
LRRC32		IER5		IFRD2	ESPL1	ASB2	
MYEOV		RND1		DDX31	RNF126	ITPRIPL1	
KLHL21		NFE2L2		ILF3	MRPL14	DTX1	
CD97		CD80		PKM	ZC3H7B	CYB561A3	
NEDD4L		REL		MRPL36	DNPH1	GALNT14	
RHOBTB3		CYLD		LSAMP	ATG16L2	MTFP1	
GALNT2		FLNA		NOP14	ERAP1	RAPGEF5	
ITPKC		OPTN		ISG20L2	HNRNPL		
SSTR2		CHST11		FKBP11	ZNF664		
TMEM178B		EFHD2		NDUFAF4	C17orf89		
RHOG		PPP1R15B		ATAD3B	ZZZ3		
WDFY1		NFKBIB		ADO	DGAT2		
CD70		IFNGR2		MRTO4	SLC17A9		
ZNF267		ICAM1		NCOA3	EPB41		
ECE1		TNFRSF10B		WDR74	LIMD2		

Supplementary Table 2
