# Supplementary material for: Transcriptional outcomes and kinetic patterning of gene expression in response to NF-κB activation
Source: PLoS Biol. 2018 Sep 10;16(9):e2006347. doi: 10.1371/journal.pbio.2006347 (PMC6147668; doi:10.1371/journal.pbio.2006347)
Supplement: S3 Table — List of genes whose expression was reduced by dnIκBα but which did not bind RELA. We refer to these genes as “indirect” RELA targets (see text). Genes in the list were changed in expression ≥2-fold in response to P+I treatment in the absence of tetracycline. Genes marked in red contain the bHLH protein binding motif (CANNTG) in their promoters (−400 to +100 bp). This motif—which is the recognition site of MYC, MITF, AHR, and NPAS2—is enriched in indirectly up-regulated genes (see text and S4 Fig). AHR, aryl hydrocarbon receptor; bHLH, basic helix-loop-helix; dnIκBα, dominant negative NFKB inhibitor alpha; MITF, microphthalmia-associated transcription factor; NPAS2, neuronal PAS domain protein 2; P+I, phorbol 12-myristate 13-acetate and ionomycin. (PDF) [file pbio.2006347.s009.pdf]

IL2	RP11-157D23.2
AC019172.2	IL8
MIR146A	CCL3L3
BARX1	RP11-733O18.1
SLC12A7	CCL4L2
BCL2A1	TFEC
LINC00158	PCLO
KIF26B	CNIH2
SERPINB9	C12orf79
EML5	PPP4R4
GPR3	SPRY1
TMCC3	NRG4
SLC19A2	RP11-141M3.5
TGIF1	SIRPA
DENND5A	AC093734.1
POLR1C	CCND1
PLA2G4C	RRP12
NT5DC3	MAFK
SRXN1	TNFRSF10A
MSMO1	FAM57A
ABLIM1	FGF2
DNTTIP2	RAB3IP
NIP7	SRFBP1
ZNF324	RP11-861A13.4
NBPF10	B4GALT5
GNL2	NAMPTL
NAMPT	C4orf32
MFSD2A	LTA
URB2	AEN
PDP2	TRMT10C
GADD45A	DIEXF
FASN	TXNRD1
TXLNG	RCL1
NAF1	BUB1B
USP31	PELO
PNO1	RP11-203B7.2
TET3	CCL5
HOXC4	HMGCS1
CCL22	
GTPBP4	

Supplementary Table 3
