# Supplementary material for: Transcriptional outcomes and kinetic patterning of gene expression in response to NF-κB activation
Source: PLoS Biol. 2018 Sep 10;16(9):e2006347. doi: 10.1371/journal.pbio.2006347 (PMC6147668; doi:10.1371/journal.pbio.2006347)
Supplement: S4 Table — Eighty-five RELA-binding genes whose inducible expression was up-regulated by dnIκBα expression. Genes marked in red were affected ≥2-fold in response to P+I treatment in the absence of tetracycline. Genes marked in green have not been previously noted in databases of NF-κB target genes. dnIκBα, dominant negative NFKB inhibitor alpha; NF-κB, nuclear factor kappa B; P+I, phorbol 12-myristate 13-acetate and ionomycin. (PDF) [file pbio.2006347.s010.pdf]

FOSB	FOS	LAMA5	MIDN
ZNF165	NR4A3	ARHGAP44	SETDB2
MYADM	NR4A1	RCOR1	HECA
GDF15	KLF6	TOB1	STAT3
BACH2	IL1RN	CALM1	CASC5
SDC3	DUSP1	HBP1	UBE2D2
SETX	ZFP36	BIN2	NCOA7
LINC00963	GADD45B	CAMK2B	EGR1
ZNF554	BTG2	ATF7IP	
DDIT3	IER2	RAB11FIP3	
ZBTB43	SLC3A2	GDI2	
TMEM2	AFF1	CENPU	
RAB11FIP1	PNRC1	HMGN2	
FAM102A	NAB2	DIAPH1	
CTNNA1	ITPKB	DBNDD1	
PPL	SMARCA2	GNG7	
PLCL2	CYTH4	PCDH1	
ZSWIM4		EPHA5	
BAIAP3		MCTP2	
NPIPB11		CAB39L	
FAM53C		GRAMD4	
KLHL24		ZNF395	
SLC16A6		PLCG2	
ARAP1		CCDC88A	
ARID3A			
MEF2B			
ARRDC3			
TCF4			
NCOA2			
TSC22D1			
ZNF763			
KCNN3			
MEF2C			
SNX29P2			
PLEKHA2			
PTPN6			

Supplementary Table 4
