# Supplementary material for: Transcriptional outcomes and kinetic patterning of gene expression in response to NF-κB activation
Source: PLoS Biol. 2018 Sep 10;16(9):e2006347. doi: 10.1371/journal.pbio.2006347 (PMC6147668; doi:10.1371/journal.pbio.2006347)
Supplement: S5 Table — List of genes whose expression was increased by dnIκBα but which did not bind RELA. We refer to these genes as “indirect” RELA targets (see text). Genes in the lists were changed in expression ≥2-fold in response to P+I treatment in the absence of tetracycline. dnIκBα, dominant negative NFKB inhibitor alpha; P+I, phorbol 12-myristate 13-acetate and ionomycin. (PDF) [file pbio.2006347.s011.pdf]

CDH17	TCP11L2	PIK3CG
PPARG	PPP1R10	SLFN5
ANKRD24	SH2D2A	PAG1
MUC2	RNF103	ZNF423
LINGO1	BSDC1	TP53INP1
BAIAP2	DOCK11	CMPK2
JUN	CREBRF	PLEKHA5
VGF	RAPGEF2	NUGGC
LINC00659	PELI1	DAAM1
TSC22D3	RAB43	ETS1
EIF2AK3	JOSD1	LRMP
FAM46A	UBC	EVI2B
CEP170B	PBXIP1	TBC1D10C
MCL1	SMG1P1	BMF
KLK1	KANK1	TXNIP
RNF19A	TLR6	HOXC4
TP53INP2	CBX4	CTC-435M10.3
MXD1	ATP2A3	
FLCN	CD38	
ENO2	LEPREL2	

Supplementary Table 5
