# Supplementary material for: Transcriptional outcomes and kinetic patterning of gene expression in response to NF-κB activation
Source: PLoS Biol. 2018 Sep 10;16(9):e2006347. doi: 10.1371/journal.pbio.2006347 (PMC6147668; doi:10.1371/journal.pbio.2006347)
Supplement: S6 Table — (A) Fifty out of 130 direct NF-κB target genes have Pol II binding in unactivated cells. (B) Pol II–associated loops in unactivated cells of direct RELA target genes (130 genes, column 1 and 2) and indirect RELA target genes (78 genes, column 3 and 4) that are induced more than 2-fold in response to cell activation. Genes with preformed Pol II–associated loops are in the first column of each set. NF-κB, nuclear factor kappa B; Pol II, RNA polymerase II. (PDF) [file pbio.2006347.s012.pdf]

A

SNX9	RGS16	OTUD4
SGK1	IER5	MYC
PPP1R15A	TRAF1	TNFRSF10B
NAALADL2-AS2	RND1	DDX21
NFKB1	NFE2L2	GABPB1
GADD45B	REL	GPBP1
TNFAIP3	ZNF267	SERPINA9
MAP3K8	PPP1R15B	ZNF106
NFKBIZ	ELMSAN1	MAK16
RHOBTB3	UBALD2	GAS7
IL10	CD70	IRF2BP2
JUNB	RHOG	CD80
PIM1	IFNGR2	POU2AF1
BTG2	SSTR2	CBX6
LRRC32	IFIH1	CFLAR
HERPUD1	ARHGAP24	NOTCH2
ZBTB10	CLEC17A	

Supplementary Table 6

B

Direct-loop	Direct- noloop	Indirect-loop	Indirect- noloop
JUNB	CCL3L1	GNL2	RP11-157D23.2
RHOBTB3	SLC22A1	DNTTIP2	IL8
FLNA	CCL4L1	ABLIM1	CCL3L3
ZC3H12A	SNX9	TXNRD1	IL2
TRAF1	CCL4	TGIF1	RP11-733O18.1
LRCH1	IL36RN	C4orf32	AC019172.2
SOD2	NFKBIZ	MSMO1	MIR146A
CBLN2	IL36B	NAMPT	CCL4L2
PPP1R15A	NFKBID	TFEC	PCLO
MAK16	LIF	GTPBP4	BARX1
DOT1L	CACNA1E	CCND1	BCL2A1
IL4I1	EBI3	ZNF324	LINC00158
RHOG	TNFAIP3	MFSD2A	CNIH2
DUSP22	MIR155HG	GADD45A	KIF26B
NFKBIE	PLAU	SLC19A2	C12orf79
SSTR2	HES1	RRP12	PPP4R4
ECE1	UTP3	HOXC4	SERPINB9
PDXK	PPAN	FASN	SPRY1
KLHL21	CLEC17A	TRMT10C	EML5
NFKB2	KIF25-AS1	HMGCS1	GPR3
ITPKC	NAALADL2	LTA	NRG4
TNFRSF10B	RILPL2	USP31	RP11-141M3.5
CSTB	NFKB1	SLC12A7	SIRPA
STAT5A	PLEKHA7	RCL1	TMCC3
MYC	RP11-672A2.6		AC093734.1
GADD45B	NAALADL2-AS2		MAFK
NR1D1	APOBEC3B		DENND5A
CD80	NFKBIA		TNFRSF10A
GCLC	PHACTR1		POLR1C
PIM3	SACS		FAM57A
STK10	OTUD4		FGF2
SGK1	UBTD2		PLA2G4C
DNAAF2	UBALD2		RAB3IP
LRRC32	MAPK6		NT5DC3
IER5	IGF2R		SRXN1
ICAM1	RELB		SRFBP1
IER3	MYEOV		RP11-861A13.4
RGS16	CD97		B4GALT5
ZFP36L2	NEDD4L		NAMPTL
EMP3	ZBTB10		NIP7
CD83	GALNT2		NBPF10
CIITA	TMEM178B		AEN
MAP3K8	WDFY1		DIEXF
IL10	ZNF267		URB2
HERPUD1	CYLD		PDP2
IRF2BP2	OPTN		TXLNG
SNAPC4	SLC7A1		NAF1
BTG2	GAS7		PNO1
NFE2L2	GABPB1		TET3
POU2AF1	EFHD2		BUB1B
MAP2K3	CBX6		PELO
KLF10	PPP1R15B		RP11-203B7.2
ABTB2	SERPINA9		CCL22
JAM2	RP11-481J2.2		CCL5
RND1	GPBP1		
REL	ZNF106		
CHST11	NFKBIB		
ARHGAP24	IFNGR2		
PIM1	CTB-58E17.1		
CD70	PLXNA1		
TNIP2	NOTCH2		
ELMSAN1	CFLAR		
UBTF	IFIH1		
DDX21	IL27RA		
MYO1C	KLHL18		

Supplementary Table 6
